# Perceptual learning of fine contrast discrimination changes neuronal tuning and population coding in macaque V4

**DOI:** 10.1038/s41467-018-06698-w

**Published:** 2018-10-12

**Authors:** Mehdi Sanayei, Xing Chen, Daniel Chicharro, Claudia Distler, Stefano Panzeri, Alexander Thiele

**Affiliations:** 1 Institute of Neuroscience, Newcastle University, Framlington Place, Newcastle upon Tyne, NE2 4HH UK; 2 Laboratory of Neural Computation, Istituto Italiano di Tecnologia, 38068 Rovereto, Italy; 3 Department of Neurobiology, Harvard Medical School, Boston, MA 02115 USA; 4 Allgemeine Zoologie und Neurobiologie, Ruhr-Universität Bochum, 44801 Bochum, Germany

## Abstract

Perceptual learning, the improvement in perceptual abilities with training, is thought to be mediated by an alteration of neuronal tuning. It remains poorly understood how tuning properties change as training progresses, whether improved stimulus tuning directly links to increased behavioural readout of sensory information, or how population coding mechanisms change with training. Here, we recorded continuously from multiple neuronal clusters in area V4 while macaque monkeys learned a fine contrast categorization task. Training increased neuronal coding abilities by shifting the steepest point of contrast response functions towards the categorization boundary. Population coding accuracy of difficult discriminations resulted largely from an increased information coding of individual channels, particularly for those channels that in early learning had larger ability for easy discriminations, but comparatively small encoding abilities for difficult discriminations. Population coding was also enhanced by specific changes in correlations. Neuronal activity became more indicative of upcoming choices with training.

## Abstract (web summary)

Perceptual learning, the improvement in perceptual abilities with training, is thought to involve changes in neuronal 'tuning'. Here, the authors show that perceptual learning works by making neurons increasingly sensitive to task-relevant differences in stimuli, and by improving population coding mechanisms.

## Introduction

Perceptual learning describes the phenomenon of improved sensory discrimination abilities that occur with training throughout life. Training-induced perceptual improvements in simple visual discrimination tasks co-occur with alterations of activity and tuning of single neurons across many subcortical and cortical areas^1–14^, whereby the extent of changes at different levels of the processing hierarchy remains debated (e.g. refs. ^3,5,13,15,16^). In addition, improved input decoding in high-level areas, possibly due to synaptic re-weighting and/or the altered correlation structure of input activity^13–16^ may be important, which requires analysis of multiple simultaneously recorded neurons, aka population activity, while learning progresses. Most prior studies have performed single-electrode recordings, comparing pre-training activity to post-training activity, or activity from trained to untrained animals or hemispheres (e.g. refs. ^9–12^). Only one study has analysed striate cortex (V1) activity from multiple chronically implanted electrodes during learning. It reported that training improves coding abilities of neuronal populations by signal enhancement, while reduction in neuronal (correlated) noise made no contribution^8^.

To address the population coding mechanisms of perceptual learning in a mid-level visual area, we recorded from chronically implanted electrodes in macaque area V4, while monkeys performed a two-alternative forced choice (2-AFC) contrast discrimination task. We used a contrast discrimination task for two reasons. First, it remains debated to what extent perceptual learning occurs in the contrast domain^17–21^. Second, the activity of most visual neurons is tuned to contrast^22–30^, thereby maximizing the number of informative channels/neurons to be included in the analysis.

Continuous recordings from chronically implanted electrodes provide insight into whether coding abilities improve homogeneously across all neurons in the population or whether they improve only within specific neuronal subpopulations (e.g. the neurons that are the most informative at the start of learning)^8–10^. Additionally, it allows examination of how perceptual learning affects the scaling of information with population size using the same sets of neurons, hence assessing the benefits of population coding over single-cell coding. Importantly, it reveals if and how changes in population codes with training depend on learning-induced changes of correlations of firing rates of different neurons^8,13,14,16^.

Improved perceptual performance was accompanied by alterations in the neuronal contrast response function (CRF), such that improved sensitivity occurred predominantly at the discrimination boundary. Training increased how much information individual neurons and neuronal populations encoded about contrast differences. The information increase was most pronounced in neurons that in early learning had higher information for easy discriminations but comparatively low information for difficult discriminations. Increases in the encoded stimulus information were accompanied by increases in the behavioural readout of these neurons, suggesting that a gain in information encoding translates directly into a gain in performance. Most of the information increases at the population level stemmed from increases in single-channel information. However, this was accompanied by a reduction in noise correlations and a change in the slope of the relationship between signal and noise correlations with learning, which also favoured the coding abilities of neuronal populations.

## Results

### Task

Two monkeys performed a 2-AFC task^17^, where they discriminated whether a test stimulus had a higher or lower contrast than a preceding sample stimulus. The sample stimulus contrast was fixed at 30%. The test stimulus contrast varied between 10% and 60% contrast in 14 steps (10, 15, 20, 25, 27, 28, 29, 31, 32, 33, 35, 40, 50 or 60% contrast). Sample and test stimuli were each presented for 512 ms, with a delay of 512–1024 ms between stimuli (Methods for details). Monkeys indicated whether the test stimulus had higher or lower contrast by making a saccade to one of the two targets appearing 512 ms after test offset (Supplementary Figure 1 for a task sketch and timeline). Both sample and test stimuli were presented in the same visual field location that covered the aggregate receptive fields (RFs) of the channels recorded (Supplementary Note 1/Supplementary Figure 5; for additional details, see Methods).

### Data set and analyses

Spiking activity was obtained from chronically implanted Utah arrays (Methods). We refer to small multi-unit neuronal clusters, recorded from a given electrode, as ‘channels’. We recorded from 29 and 20 channels in monkey 1 and monkey 2, respectively. These yielded good responses (signal-to-noise ratio [SNR] >1) on >80% of the recording days (Methods). To obtain comparable activity levels across sessions, we performed baseline activity matching between sessions for multi-unit activity (MUA) data (Methods; controls how baseline activity matching could affect results are given in Supplementary Note 10, Supplementary Figure 21-22). Additionally, we recorded stably from a few single units throughout all the recording sessions, which yielded qualitatively identical data to multi-unit analyses (Supplementary note 9, Supplementary Figure 12-20).

For all the main analyses, we used a 256 ms long analysis window, which was empirically determined to maximize the information encoded about the stimuli. In monkey 1, this window started at 30 ms after stimulus onset, and it started at 158 ms after stimulus onset in monkey 2 (see Methods for details and additional controls for justification).

### CRFs and neurometric functions

Contrast tuning was assessed by fitting a Naka–Rushton function to the single-channel response data of each session. The Naka–Rushton fit yielded: (1) the slope of the tangent to the best-fitted Naka–Rushton function at a contrast level of 30% (the sample contrast). The steeper the slope at (and around) 30% contrast, the better the channel was at discriminating between stimuli with contrasts close to the sample contrast (the categorization boundary). (2) *C*_50_, the contrast that elicited a response of half the response range. (3) The minimum and (4) maximum values of the best-fitted Naka–Rushton function.

To calculate neurometric functions and neuronal discriminability, we performed area under the receiver operating characteristic (AUROC) analyses. We also devised a novel approach to quantify neuronal stimulus discriminability (NSD), which is applicable where decisions in a 2-AFC task are based on comparison of two stimuli (e.g. test and sample stimulus) that are presented within a single trial. The approach was termed count-based estimator (COBE, described in full in Supplementary Note 11). It provides a hypothesis-free measure of neural discriminability taking into account the effects of slow activity fluctuations. Its performance is superior to traditional AUROC approaches, if neuronal activity is subject to slow excitability fluctuations, as is omnipresent in neuronal systems^31^. We present the AUROC data in the main manuscript and present the COBE data and a direct comparison of AUROC and COBE measures in Supplementary Note 12 (Supplementary Figures 24-26).

**Fig. 1:**
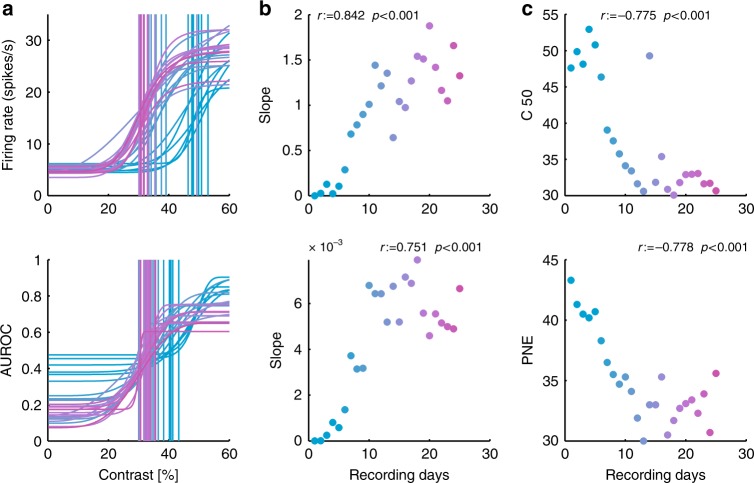
Example channel. **a** Single-channel contrast response functions and neurometric function as a function of learning (colour coded transitions red to blue). Vertical lines show location of *C*_50_ for each recording day. **b** Slope of the contrast response function and neurometric function at 30% (the sample contrast). **c** Change of the *C*_50_ and the PNE with learning

To detect changes in neurometric functions, we monitored the point of neuronal equality (PNE; Methods), which is the point where sample and test stimulus-elicited activity is identical (AUROC = 0.5). Changes in the slope of the CRF and of the neurometric function at 30% contrast, as well as changes in *C*_50_ and the PNE, of an example channel are shown in Fig. 1a–c. The CRF and neurometric function became steeper (at 30% contrast) over the course of training (Fig. 1b). Moreover, the *C*_50_ and the PNE shifted towards the value of 30% with training (Fig.1c). The example shown in Fig. 1 reflects the pattern seen across the population.

**Fig. 2:**
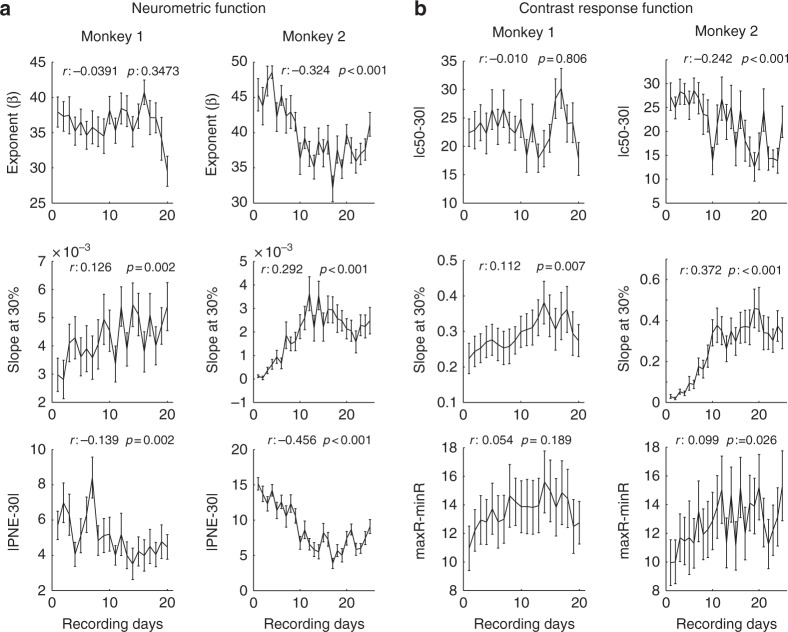
Learning-induced changes in selected parameters of the neurometric function and of the contrast response function. **a** Changes in location where the neurometric function reaches 63% of its range, the slope at 30% contrast and point of neuronal equality of the neurometric function. **b** Changes in *C*_50_ of the Naka Rushton function, its slope at 30% and rate ranges (difference between minimum and maximum measured activity). Insets show the Spearman rank correlation coefficients (*r*) and the *p* value (*p*) of the parameter of interest (dependent variable) vs. recording days (independent variable). Error bars show S.E.M. (*n* = 29 and *n* = 20 per data point for monkeys 1 and 2, respectively)

**Fig. 3:**
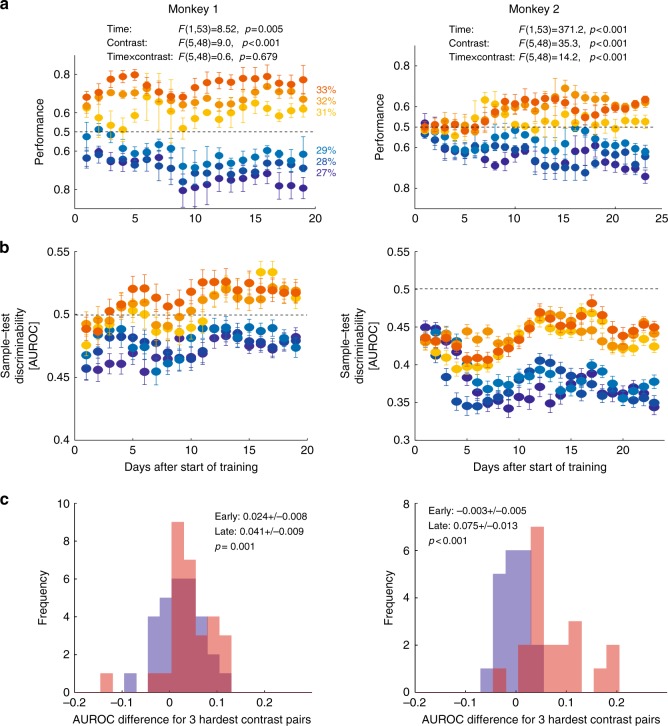
Changes in behavioural performance and neuronal discriminability with learning. **a** Behavioural performance for the six most difficult contrast discriminations. Performance for test contrasts lower than sample contrast (blueish colours) is plotted from 0.5 to 1 downwards. Performance for test contrasts higher than sample contrast (reddish colours) from 0.5 to 1 is plotted upwards. Test contrast colour assignment is given by coloured number insets. **b** Neuronal discriminability (AUROC) for sample–test contrast in the two monkeys with learning. Sample–test contrast colour assignment is given by coloured number insets in **a**. **c** Distribution of discriminability difference for the 3 most difficult sample test–contrast comparison pairs (e.g. 31% AUROC–29% AUROC, 32% AUROC–28% AUROC, 33% AUROC–27% AUROC) for the first 5 days of learning (blue) and for the last 5 days of learning (red) across all channels recorded. Darker red shades show overlap of the two distributions. Insets display the mean and S.E.M. of the two distributions. *p* Values indicate whether distributions differed significantly. Performance and discriminability for each data point is the average over 3 consecutive days, i.e. error bars in **a**, **b** denote S.E.M. of a 3-day performance (AUROC) average (thus the number of data points are the total number of recording days minus 2)

To calculate whether parameters of our fitting functions changed over time, we calculated Spearman rank correlations over averages across channels per session (*n* = 21 days for monkey 1; *n* = 25 days for monkey 2). The slopes of the CRF at 30% steepened significantly in both monkeys (Fig. 2, Spearman’s rank correlation, monkey 1: *p* = 0.007; monkey 2: *p* < 0.001). The *C*_50_ of the CRF shifted towards the sample contrast (30% contrast) in monkey 2 but not in monkey 1 (Fig. 2b, Spearman’s rank correlation, monkey 1: *p* = 0.806; monkey 2: *p* < 0.001). No consistent effects were found when analysing the difference between the minimum and maximum activity levels. Similar results were obtained for neurometric functions. The slope of the neurometric function at 30% contrast increased significantly (Fig. 2a, Spearman’s rank correlation monkey 1: *p* = 0.002; monkey 2: *p* < 0.001). Moreover, the PNE shifted towards the sample contrast (towards 30%, Spearman’s rank correlation, monkey 1: *p* = 0.002; monkey 2: *p* < 0.001). Additionally, the exponent *β* of the Weibull function became smaller in monkey 1 (*p* = 0.023) but not in monkey 2 (*p* = 0.178). None of the other parameters of the Naka–Rushton or neurometric functions changed in monkey 1. In monkey 2, the scale parameter *α* of the Weibull function significantly decreased with learning (*p* < 0.001, Spearman’s rank correlation, indicating that the function at 63% of its range became more shallow). Similarly, the exponent of the Naka–Rushton function significantly decreased with learning in monkey 2 (*p* < 0.001, Spearman’s rank correlation) but not in monkey 1. Overall, these data suggest that neurons became more sensitive at discriminating between contrasts levels that were close to the sample contrast (30%), making neurons more sensitive at the categorization boundary. These changes mirror the changes seen at the behavioural level^17^ (Fig. 3a for behavioural data).

As evident from Fig. 2, PNE across the population was >30% (the sample contrast), an effect caused by short-term adaptation. Additionally, PNE moved towards 30% with learning. This could suggest that the changes seen are caused by changes in short term adaptation. However, we can rule out that changes in PNE with training generally resulted from reduced short-term adaptation (Supplementary notes 2 and 3, Supplementary Figure 7 and 8).

The changes were equally not induced by training-induced changes in spatial attention (Supplementary note 5).

Finally, the changes in tuning were restricted to the contrast domain and did not extend to orientation or spatial frequency tuning (Supplementary note 8).

### Changes in test–sample neuronal discriminability with learning

Behavioural changes with learning for the most difficult contrasts occurred in both monkeys (Fig. 3a). A two-factor analysis of variance (ANOVA) showed that performance differed significantly as a function of contrast (factor 1) and as a function of training day (factor 2) in both monkeys. There was no interaction between the two factors in monkey 1, but there was in monkey 2 (see insets in Fig. 3a for *p* and *F* values). For this analysis, we used values obtained on individual days, not those obtained by averaging across 3 consecutive recording days. This approach ensures independence of samples, and it was applied to all statistical tests performed throughout the paper.

Learning-induced changes of neuronal discriminability was quantified using ideal observer approaches comparing sample and test contrast-evoked activity (e.g. the difference between 30% and 29% contrast), for each day and channel. We present the analysis for the AUROC data in the main text and the respective COBE data in Supplementary Note 12 (Supplementary Figure 24-26). Higher values of AUROC indicate higher discriminability (0.5 is chance, 1 is perfect discrimination). The 14 different test contrasts yielded 14 groups of AUROC values for each recording session. We focus on the 6 contrast levels that were closest to the sample contrast, namely the 3 contrasts just above (31, 32, and 33% contrast) and just below (27, 28 and 29% contrast) the sample contrast. These were the most difficult discriminations behaviourally, where clear performance changes occurred (Fig. 3a). Average AUROC for these contrasts as a function of learning are shown in Fig. 3b. These data suggest that AUROC differences (between lower and higher test contrasts) increased with learning, i.e. AUROCs on the two sides of the categorization boundary became more separated. To quantify this, we calculated AUROC differences between 27% and 33%, between 28% and 32%, and between 29% and 31% test contrast for the first 5 days and last 5 days of training. The difference distributions for these two training periods are shown in Fig. 3c. Training significantly increased the differences in both monkeys (*p* < 0.001 in each animal individually, two-sided Wilcoxon sign rank test, AUROC values were averaged separately across the early and late days for each channel, and across contrast differences, i.e. *n* = 29 channels for monkey 1 and *n* = 20 channels for monkey 2, Fig. 3c). This shows that both behavioural and neuronal discriminability improved for difficult contrast differences.

We also observed several changes for the easiest contrast differences. Behaviour changed significantly with training in both monkeys, particularly for monkey 2 (Supplementary note 4, Supplementary Figure 9). In monkey 1, no significant changes in AUROC occurred with training, whereas in monkey 2, the AUROC changed significantly (Supplementary Figure 9).

### Learning-induced changes of single trial test–test discriminability

The analyses of sample–test data showed a marked increase in the information that was available in individual channels, with training. This suggests that the encoding of test–test contrast differences should also have increased with learning, particularly for contrasts on opposite sides of the decision boundary, due to the categorical nature of the task.

**Fig. 4:**
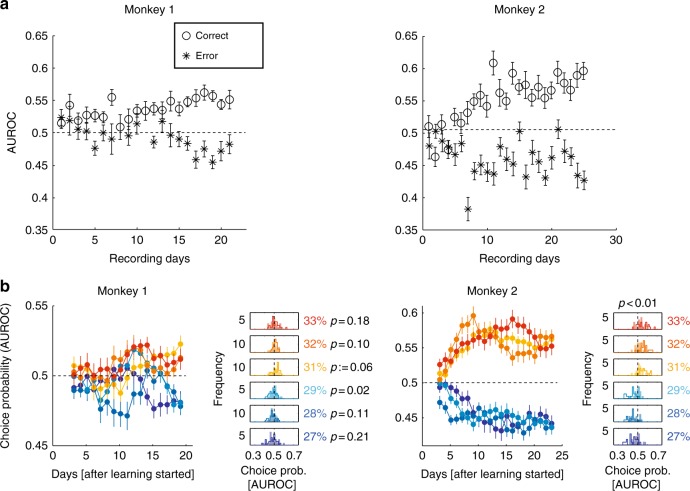
Relation of neuronal activity to choices. **a** Average test–test AUROC values for the three hardest contrast pairs as a function of learning, analysed for correct and error trials. Error bars show S.E.M.s for averages across the 3 contrast pairs and 3 consecutive learning days (note that some error bars are smaller than the symbols and thus close to invisible). **b** Choice probability as a function of learning for both monkeys for different test contrasts (colour coded and displayed as insets). Left column: Choice probability for test activity levels (separately for the three hardest contrast levels below and above sample contrast, respectively). Insets in **b** show distributions of choice probabilities for the first 5 days of learning (filled histograms) and the last 5 days of learning (outlined histograms). *p* Values for distribution differences are indicated above the top histogram for each main comparison and next to the individual histogram plots if different thereof . Data in **b** are averaged over 5 consecutive days, i.e. number of data points = recording days minus 4. Error bars denote S.E.M. (*n* = 29 and *n* = 20 per data point for monkeys 1 and 2, respectively)

We thus calculated AUROCs for the following test–test contrast pairs: 29–31%, 28–32%, and 27–33% contrast. These were calculated for the first 5 days of learning and for the last 5 days of learning, respectively. We analysed correct and error trials separately. Test–test discriminability increased significantly with training for correct trials in both monkeys (Fig. 4a, monkey 1: *p* < 0.001, monkey 2: *p* < 0.001, two-sided Wilcoxon sign rank test). For error trials, AUROC discriminability significantly decreased in both monkeys (Fig. 4a; monkey 1: *p* < 0.001, monkey 2: *p* = 0.029, two-sided Wilcoxon sign rank test), changing from AUROC values of >0.5 (or close to 0.5) to values <0.5. This suggests that during late stages of training error trials associated with test contrasts <30% yielded responses that were larger than responses on error trials associated with test contrasts >30%. Thus learning led to changes in choice probability (CP).

### CP analysis

To determine whether training affected the degree to which the monkeys’ upcoming decision was reflected in the neuronal responses, we computed CPs (see Methods for details). This was done for each channel as a function of time after training onset (Fig. 4b, with a 3-day running average). Calculations of CP required a sufficient number of incorrect as well as correct trials, hence this analysis focussed on data obtained from the six most demanding test contrast conditions. CPs closer to zero (relative to 0.5) corresponded to the selection of the ‘lower test contrast’ target, while CPs closer to one corresponded to the selection of the ‘higher test contrast’ target. If neuronal activity in our target areas became more effective in influencing the animal’s upcoming decision (or the readout of sensory information improved), then CP values for test contrasts of <30% should have decreased over the course of training, while CP values for test contrasts of >30% should have increased.

To determine whether training significantly affected the CP distributions, CPs were pooled separately across the first and last 5 days for each recording channel and for each monkey. The results are shown in Fig. 4b. A two-way ANOVA was calculated, with training period (early or late days) and test contrast as factors to determine whether learning or test contrast affected CPs. In both monkeys, a significant effect of training period was observed (monkey 1: test contrast: *F*(5,336) = 1.57, *p* = 0.142; training period: *F*(1,336) = 4.99, *p* = 0.02; interaction: *F*(5,336) = 1.29, *p* = 0.27; monkey 2: test contrast *F*(5,228) = 1.21, *p* = 0.30; training period: *F*(1,228) = 42.46, *p* < 0.001; interaction: *F*(5,228) = 4.58, *p* < 0.001).

Post hoc one-sided *t* tests were performed to compare the means of the distributions between early and late days for each of the 6 hardest contrast conditions (CPs were averaged separately across early and late recording days for each channel [*n* = 29 for monkey 1 and 20 for monkey 2]). These distributions are shown in the small subplots of Fig. 4b ( right column), along with the associated *p* values. For all test contrast conditions, CPs became more informative of the upcoming choice in both monkeys. In summary, with training, CP values became increasingly representative of the animals’ upcoming choice, and the magnitude of changes observed could be as large as 0.08(8% improvement in the performance of an ideal observer).

We next analysed whether choices on error trials were determined by sample–test activity differences, rather than by absolute levels of activity elicited by the test stimulus (note that the latter is the basis of the above described CPs). The reasoning is that the monkey potentially made ‘higher contrast’ decisions on trials when test activity exceeded sample activity, and ‘lower contrast’ decisions when test-evoked activity was lower than sample-evoked activity. Hence, we calculated the difference in responses to sample and test stimuli for each channel (conditioned upon the monkey’s choice) and calculated the AUROC on the resulting two response difference distributions. This measure is the COBE analogue for CPs. The test–sample CP approach resulted in smaller CP values for early and late training periods than the approach where sample activity was not factored out. For this analysis, there was a significant main effect of training on test–sample CP values for monkey 2, while the training effect for the sample minus test CP analysis in monkey 1 did not show a main effect but an interaction, i.e. it was dependent on the test contrast (monkey 1: test contrast: *F*(5,336) = 0.64, *p* = 0.668; training period: *F*(1,336) = 1.08, *p* = 0.299; interaction: *F*(5,336) = 2.63, *p* = 0.02; monkey 2: test contrast *F*(5,228) = 3.2, *p* = 0.008; training period: *F*(1,228) = 26.29, *p* < 0.001; interaction: *F*(5,228) = 3.06, *p* = 0.01). The discrepancy between traditional CP and test–sample CP values suggests that decisions were determined by absolute activity levels in response to the contrast of the test stimulus, rather than being determined by the difference between activity levels that were evoked by the test and sample stimuli.

### Effect of learning on information coding in different channels

**Fig. 5:**
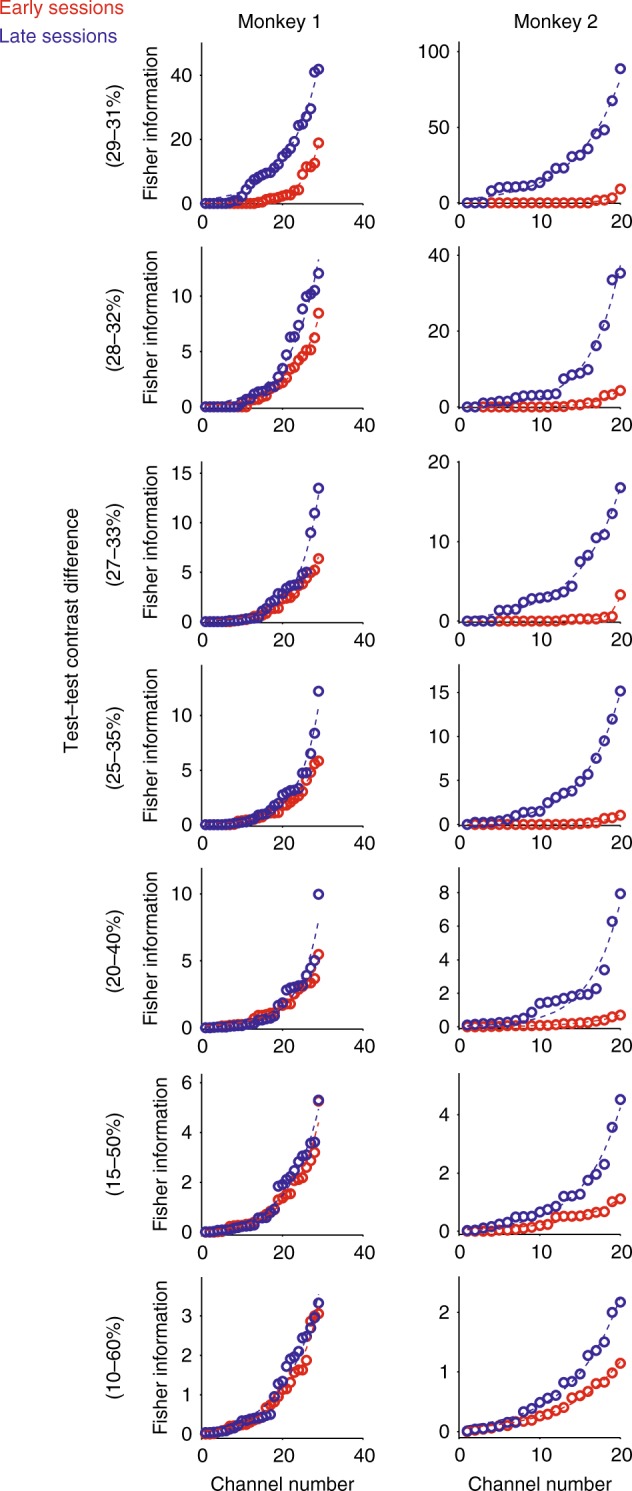
Distribution of information content across channels. Rank-ordered information content in single channels for different contrast pairs during early (red) and late (blue) periods of learning. In both monkeys, a sizeable number of channels encoded relatively little information during early or late training days. Differences between training days are most obvious for channels with comparatively high information content

To assess whether differences in contrast information coding abilities exist across channels and whether learning affects all channels equally, we performed linear Fisher information analysis, according to ref. ^32^ (Methods for details). Information was calculated for the following test–test contrast pairs: 10–60%, 15–50%, 20–40%, 25–35%, 27–33%, 28–32%, and 29–31%. For each channel, we calculated the amount of information it encoded for the different test contrast pairs during the first 5 days of learning and during the last 5 days of learning, when animals made correct decisions. Channels were ranked based on the amount of information encoded during the first 5 training days and separately based on the last 5 training days. The information content varied substantially between channels (Fig. 5). The rank-ordered data were fitted with an exponential function of the form: Pred(info) = *c* + *b* × (1 − e^(*λ*(channel number))^)^33^, which yielded excellent fits to the data. For most of the fits, the variance accounted for was >99%, and the smallest value of variance accounted for was 85.7%.

Figure 5 shows that changes in information content with learning did not occur equally across channels. The largest differences appeared at the end of the distributions, for channels with high information content, rather than simply generating an offset of the two functions. However, given that the sorting of information content was done separately for early and late training days (i.e. the identity of the channels was not preserved), the analysis does not reveal whether it was the channels with high information during early training days that underwent the greatest increases in information content.

To investigate this, we calculated the level of correlation between information content during early recording days, during late recording days and between early vs. late recording days across channels within contrast pairs and between contrast pairs (*n* = 29 channels for monkey 1 and *n* = 20 channels for monkey 2). During early training, neurons that encoded more information for pairs with the largest contrast difference also encoded information for contrast pairs with moderate contrast differences (positive correlation of information values, Supplementary note 6, and Supplementary Figure 10B) but not necessarily for small contrast differences (weaker correlations). Conversely, during late training, we found positive correlation between information values across the entire range of contrast differences (Supplementary Figure 10C). Thus, during late training, neurons that encoded more information for large contrast differences also encoded more information about small contrast differences. When comparing the information coding for large contrast differences during early periods to the coding of small contrast differences during late training, we found a positive correlation in both monkeys (Supplementary Figure 10A, for associated *p* values, see same figure). This demonstrates that channels which encode information about large contrast differences during early stages of training develop to show the largest coding abilities for small contrast differences during late training stages.

To investigate which channels benefit the most from learning in proportional terms, we focussed on the correlation between information values in the early training for each contrast difference and the proportional gain in information that is obtained with learning with the same contrast difference (the proportional information gain is defined as the difference in information between late and early training, normalized by the information encoded in early training). If information increases were proportional across all channels, we would find no correlation. If instead channels with lower information gained proportionally the most (respectively, less) during learning, then this correlation would be negative (respectively, positive if the opposite was true). We found negative correlations for all contrast pairs (Supplementary Figure 10D, for associated *p* values, see same figure). It shows that neurons with relatively small discrimination power for small contrast differences gained proportionally more discrimination power, while already selective neurons proportionally gained relatively less selectivity. Thus learning increased the number of neurons carrying some information about difficult contrast differences, thereby increasing the size of the population that could contribute to solving the task.

Changes in neuronal coding were not related to changes in coding related to motor preparation or memorizing the appropriate upcoming response given the stimulus (Supplementary note 7, Supplementary Figure 11).

### Population coding analyses

Thus far, we have analysed information content in single recording channels. We next examine how the information present at the population level changed with learning. Changes in information across the population could have been due to changes in single-channel coding (see above), but they could also be due to changes in the correlation structure (noise correlations) of simultaneously active channels. We will first examine whether noise correlations changed with training.

### Changes in noise correlations with learning

**Fig. 6:**
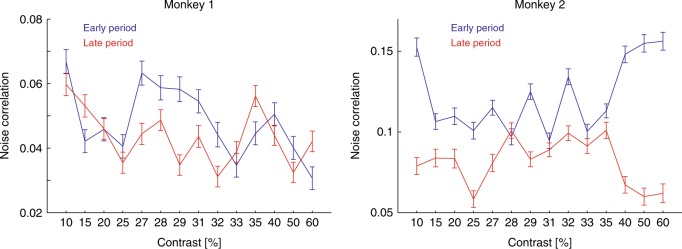
Noise correlations as a function of training period and test contrast. Left column: Average noise correlation for all pairwise comparison for different test contrasts during early experimental sessions (first 5 days, blue) and during late experimental sessions (last 5 days, red). Error bars denote S.E.M. (*n* = 29 and *n* = 20 per data point for monkeys 1 and 2, respectively)

Noise correlations were calculated for each contrast for the first 5 days of training and for the last 5 days of training, for each channel combination (see Methods for details). In both monkeys, noise correlations decreased with learning (Fig. 6; two-factor ANOVA, factor learning period: monkey 1: *F*(11364,1) = 10.38, *p* = 0.001; monkey 2: *F*(5316,1) = 276.6, *p* < 0.001), noise correlations depended on contrast (two-factor ANOVA, factor contrast: monkey 1: *F*(11364,1) = 9.24, *p* < 0.001; monkey 2: *F*(5316,13) = 4.76, *p* < 0.001), and there was a significant interaction between contrast and training (two-factor ANOVA, factor learning period×contrast *F*(11364,13) = 4.25, *p* < 0.001; monkey 2: *F*(5316,13) = 13.01 *p* < 0.001).

The training-induced changes in noise correlations were not the result of training-induced changes in attention (Supplementary note 5).

### Population information coding

Noise correlations affect coding abilities of neuronal populations^34,35^. Thus the decrease in correlations with learning might indicate that population coding abilities improved in both monkeys, not only because single-channel discriminability increased. However, the absolute size of noise correlations does not determine whether the ability of downstream neurons to decode information is affected. Rather, the sign and the magnitude of signal and noise correlations interactions determine whether noise correlations limit information coding^35–38^. Most channel pairs had same sign signal and noise correlations (a combination known to reduce information^35–38^) during early and late learning stages (monkey 1: 361/406 pairs have same sign signal and noise correlation for early days, and 362/406 for late days; monkey 2: 179/190 for early days and 184/190 for late days). Hence, learning-induced reductions in noise correlation could aid population decoding. We thus examined the amount of information as a function of the population size under conditions when we retained correlations (analysing simultaneous responses) and when we removed correlations (analysing shuffled population responses).

### Changes of information content with learning in neuronal populations

**Fig. 7:**
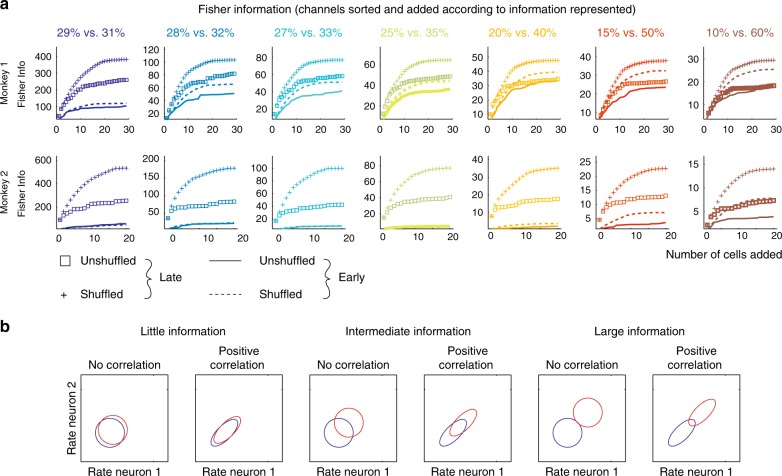
Information in populations of different sizes before and after learning. **a** Fisher information in a population of consecutively added channels for early (solid and dashed lines in each subplot) and late recording days (+ and squares in each subplot). Colour coding shows the Fisher information for the different test contrast pairs investigated/decoded. ‘Unshuffled’ indicates that the channel identity and the correlation structure was retained during calculation of Fisher information (squares and solid lines). ‘Shuffled’ indicates that trials between channels were shuffled to remove the rate correlations, while retaining channel identity (+ and dashed lines). Abscissa: number of channels that were added to the population. Ordinate: Fisher information present. Channels were added based on their rank-order single-channel information content, starting with the channel encoding the most information. **b** Cartoon that exemplifies how noise correlation reduction differentially affects population decoding abilities, depending on the amount of information represented in individual neurons. The circles/ellipses indicate the response co-variability between two hypothetical neurons. Blue circles/ellipses show correlation of responses for one potential stimulus, red circles/ellipses for a different stimulus. The further the blue and red circles/ellipses are apart the larger the information single channels represent. Two scenarios (no correlation vs. positive correlation) for three different information contents (little information, intermediate information, large information) are shown. Reduction in overlap between the two stimulus-induced response distributions is maximal when neurons have relative large information content

To investigate how changes in signal and noise correlation with learning affected the population code, we considered linear Fisher information about test contrast as a function of population size, increasing the population one channel at a time (see Methods for details). Figure 7a shows that the information content for almost all population sizes was higher during late training days than during early training days in both monkeys. The information increase (as channels were added to the population) was not linear, owing largely to the different amounts of information present in individual channels (Fig. 5), but also to the noise correlation that was present between channels (Fig. 6). This becomes apparent when comparing unshuffled and shuffled Fisher information (Fig. 7a, compare + vs. squares and solid lines vs. dashed lines). Shuffling destroyed the noise correlation and increased Fisher information in the population for late learning days for all population sizes of *n* > 3. For early training days, the difference between unshuffled and shuffled Fisher information is also present in monkey 1, but it was small in monkey 2, unless larger test contrast differences were considered (Fig. 7). The latter owes to the fact that during early periods of training, individual channels in monkey 2 did not show any sizeable information for small contrast difference, hence noise correlations cannot be detrimental to virtually non-existing information (exemplified in a cartoon in Fig. 7b).

We next compared the population information for the shuffled early days with the population information for the unshuffled late days. Figure 7a shows that the population information present in the unshuffled data of the late days is generally substantially higher than the population information in the shuffled data from the early days. Thus the single-channel information increase with learning is the key to the increase in population information, even if the reduction in noise correlation may contribute further benefits.

Figure 7a also shows that the difference in population information between shuffled and unshuffled data sets is larger during late stages than during early stages of learning. While this may seem counterintuitive, given the reduction in noise correlations with learning, it is a by-product of the fact that during early stages of learning most channels contain very little information for the difficult contrast differences, and thus noise correlations cannot be detrimental (see Fig. 7b). Phrased differently, the noise correlation reduction seen for late stages of learning may nevertheless be important, since noise correlations would have a much larger detrimental effect on population coding, had they not been reduced.

**Fig. 8:**
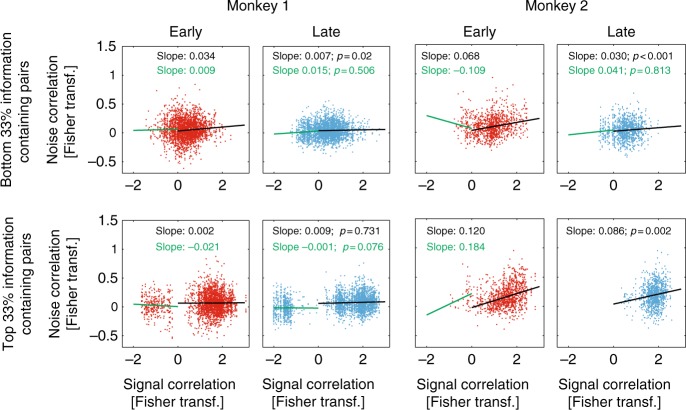
Relation between signal and noise correlation during early and late stages of learning. Top row:  Signal vs. noise correlation for channels that encode relatively little information for early (left columns for each monkey) and late (right columns for each monkey) recording sessions. Bottom row:  Signal vs. noise correlation for channels that encode large amounts of information for early (left columns for each monkey) and late (right columns for each monkey) recording sessions. Linear regressions between signal and noise correlations (Fisher transformed) were calculated separately for pairs with positive signal correlation and for pairs with negative signal correlation. Slopes for these calculations are given in each subplot (black: positive signal correlations, green: negative signal correlations). *p* Values indicate whether slopes for the late sessions were significantly different from those for the early sessions (permutation test). Red data points show signal vs. noise correlation distributions from early sessions (at the start of learning), blue data points the respective distributions from late sessions (after learning)

To investigate this possibility further, we calculated the slope between signal and noise correlations for early and late learning periods. A shallower slope enables neuronal populations to encode more information^16,39^. The slope between noise and signal correlation was calculated separately for channel pairs where both channels were part of a less sensitive population (bottom third of information coding channels) or where both channels were part of a more sensitive population (top third of information coding channels). For neuronal pairs that have positive signal correlations, the slope between signal and noise correlation was significantly decreased with training when pooled across monkeys, irrespective of information content (*p* < 0.001, two sided permutation test, see Methods). However, there were some differences between the two monkeys. If analysed individually, monkey 1 only showed significant reductions in the slope for less informative channels (positive signal correlations), while monkey 2 showed significant reductions for both channel groups (for exact *p* values and additional details, see Fig. 8 insets).

Figure 8 equally shows that channel pairs with high information content had increased signal correlations (positive or negative) after training. These results were robust with respect to which subpopulations of neurons were selected (top/bottom 20% of information content, top/bottom 33% [Fig. 8], top/bottom 50%).

While most pairs have positive signal correlation in both monkeys, some neuronal pairs show negative signal correlations (Fig. 8). For pairs with negative signal correlations, the slope between signal and noise correlation should become either shallower or more negative to improve decoding abilities in downstream decoders. However, this is not what we found. None of the slope changes for pairs with negative signal correlation were significant (two-sided permutation test, *p* > 0.1, for exact *p* values and additional details, see Fig. 8 insets).

In sum, in channel pairs with positive noise and positive signal correlations (the vast majority of channel pairs available), training generally reduced the slope of that relationship, which can improve encoding abilities of neuronal populations.

## Discussion

Training improved contrast discrimination sensitivity of macaque monkeys. Behavioural improvements were accompanied by shifts of the point of neurometric equality towards 30%, and a steepening of the slope of the neurometric function at the decision/discrimination boundary. Neurons increased their ability to represent small contrast differences. These increases occurred more strongly in neurons that at the start of training had higher information for easy discriminations but showed comparatively poor contrast sensitivity for difficult discriminations. Improved decoding at the population level was largely due to the enhanced single-channel coding abilities. However, learning also reduced noise correlations between neurons and altered the relationship between signal and noise correlations, thereby facilitating downstream decoding. Finally, we found improvements in the behavioural readout of the enhanced sensory information after learning.

Training shifted both the point of neurometric equality and the *C*_50_ of the CRF towards the contrast that formed the decision boundary (30% contrast). This was associated with an increase in the slope of the neurometric function at 30% contrast. Thus perceptual learning caused a sharpening of the tuning curve, leading to improved neuronal tuning at the decision boundary. This result is in line with previous reports, where perceptual learning of orientation differences was investigated in areas V1 or V4^9,10^ and in area V1 in cats that were trained in a contrast detection task^40^.

Switching attention between trained and untrained locations did not trigger the training-induced shift in PNE, indicating that learning-induced changes arose from long-lasting enhancements in neurons’ ability to represent stimulus contrast differences, rather than to attention-evoked or other task-evoked modulations of firing rates (see also Supplementary note 10). This is in line with data from posterior inferior temporal area, which is hierarchically close to area V4^12^. Interestingly, the results are different from similar studies in area V1^41^, where neural correlates of perceptual learning were task-dependent, and thus possibly related to selective attention^7^. However, in the V1 study^41^, decision boundaries changed regularly, whereas in our study, the decision boundary was fixed within and across recording sessions.

Learning-induced changes of noise correlations were also unaffected by spatial attention, after learning was consolidated. Thus improved spatial attention due to learning was not the main determinant of the changes observed. Notwithstanding, attention will certainly be required for task performance per se. Additionally, attention may be the initial driver which induces (or allows for) the tuning changes and correlation changes to happen. With sufficient training, these changes become self sustained, i.e. lasting properties of the network.

Training was accompanied by increases in CP, which differs from previous results. No CP changes occurred in V4 after training on a coarse orientation discrimination task^11^. In middle temporal (MT) area, modest increases in CPs were found in a motion direction categorization task^13^, while larger increases were found in a depth discrimination task^15^. The CP changes imply a tighter statistical alignment between the improved neuronal representation of sensory signals and the animal’s behavioural report or, in other words, a greater intersection between the sensory coding and its readout^42^. This tighter statistical alignment between sensory coding and its readout could have a variety of causes. Within a causal feedforward framework, it may occur due to a better readout of sensory information after learning, i.e. altered readout weights that result in increased intersection information^37,38^. This would explain the increase of behavioural performance with learning. Alternatively, the tighter statistical alignment between sensory coding and behavioural readout may have a non-causal interpretation. It is possible that the recorded neurons do not cause behaviour but are simply correlated with another V4 population that affects behaviour. However, we also found that training reduced V4 neuronal correlations, which makes the latter scenario unlikely. Finally, top–down signals could contribute to the better behavioural readout of the sensory signals. For example, V4 may receive more accurate (or stronger) decision-related feedback signals after learning. Further interventional studies are needed to determine whether the changes in CP support the feedforward^43^ or the feedback theory of CPs^44–46^.

Information coding in single neurons and neuronal populations increased significantly from early to late stages of learning. The distributions of information present in different channels were well described by an exponential function for both learning periods. Thus the representation of stimulus contrast information in the visual system is distributed in a similar manner to that of natural sounds in the primate auditory system, which also follow an exponential function^33^.

Previously, two studies on perceptual learning examined simultaneously recorded activity in pairs of medial superior temporal (MST) neurons in non-human primates^16^ or multiple neuronal ensembles in V1^8^. In MST area^16^, tuning properties did not change with learning, while noise correlations were reduced. The V1 study^8^ used a figure detection task and recorded continuously while learning occurred. They reported that neurons encoding the figure elements increased their responses, while neurons encoding the noisy background reduced their responses, thereby increasing the coding abilities of single channels and of neuronal populations. The information increase with learning in our data is similar to the information increase seen in area V1^8^. It remains unclear as to why learning does not affect tuning properties of neurons or populations in mid-level dorsal stream areas (MT, MST^15,47,48^).

The reduction in noise correlations with learning is consistent across various visual areas^8,15,47–49^, but the degree to what extent these changes benefit decoding abilities differs. Gu et al.^16^ compared the relation between signal and noise correlation of pairs of MSTd neurons recorded in two groups of macaques. One group had previously been trained in a heading discrimination task, while animals from the other group had not. Noise correlations in the trained animals were overall reduced compared to naive animals, but the slope of the regression between signal and noise correlation did not differ between the two groups, and the reduction in noise correlation itself did not benefit population coding efficiency. Yan et al.^8^ recorded continuously from neuronal ensembles in V1 and reported that a training-induced reduction in noise correlations did not benefit downstream decoders. Conversely, Ni et al.^49^ demonstrated that attention and perceptual learning reduce noise correlations and that the reduction of noise correlation strongly impacted the detection performance of the animals. We found that learning reduced the slope of the relationship between signal and noise correlations (for neuronal pairs with positive signal correlations, the large majority). This increases the amount of information that could be decoded by downstream neurons^39,50^. The difference between our data, Ni et al.’s^49^ data vs. those of Yan et al.^8^ is unclear. It is, however, noteworthy that the effects of correlated neural activity in a given area do not always affect downstream areas, and by extension, changes in correlated activity in area V1 and V4 with training may have different behavioural consequences. For example, changes in noise correlation in higher visual areas (e.g. V4) can alter noise correlations in V1 through feedback^51^, whereby they do not constrain sensory coding. At the same time, a learning-induced reduction of noise correlations in higher visual areas, such as V4, may improve sensory readout further downstream. While our results are qualitatively similar to those reported by Ni et al.^49^, the effect of noise correlation reduction on decoding ability in our data is more limited. In Ni et al.’s study, the locus of attention was regularly changed within single training sessions. This regular change could potentially ‘imprint’ the attention-induced noise correlation changes on learning-induced changes, thus making the latter more pronounced, and detectable. Moreover, Ni et al. used an orientation discrimination change detection task, while we used a categorical sample–test comparison contrast discrimination task. Finally, noise correlations reported by Ni et al. are larger than those reported here. Noise correlations between different recording channels increase with the number of cells contributing to MUA spiking activity for a given channel. Based on the differences in noise correlations between the two studies, more cells likely contributed to signals from a single electrode in their study. If this was the case, the differences would suggest that effects of noise correlation changes on performance are more readily detectable when larger neuronal samples contribute to the activity of single channels. This is also supported by our single-cell analysis (Supplementary note 9, Supplementary Figures 20), where the effect of learning on noise correlations per se, and how they affect coding abilities, were much more limited.

The reduction in noise correlation with learning is reminiscent to changes in noise correlation with attention^49,52–57^. This may potentially be due to altered levels of acetylcholine, which is known to contribute to attentional modulation in visual cortex^58^, to learning and plasticity^59,60^, as well as alterations in noise correlations^61^ and the relationship between signal and noise correlations^39^.

In summary, the improved perceptual abilities were foremost the result of increases in single neuron contrast coding abilities at the borders of the categorization boundary. These single neuron information increases were accompanied by specific changes in the correlation structure of population activity that further enhanced the information neuronal populations encode.

## Methods

### Data collection

All procedures were approved by the Newcastle University Animal Welfare Ethical Review Board and carried out in accordance with the European Communities Council Directive RL 2010/63/EC, the US National Institutes of Health Guidelines for the Care and Use of Animals for Experimental Procedures and the UK Animals Scientific Procedures Act. Two male macaque monkeys (5 and 14 years of age) were used in this study.

### Head post implantation

An initial surgical operation was performed under sterile conditions, in which a custom-made head post (PEEK, Tecapeek) was embedded into a dental acrylic head stage. Details of surgical procedures and postoperative care have been published elsewhere^62^.

### General training

Initially, monkeys were trained to perform a delayed match-to-sample task, in which they compared the colour of a circle stimulus with that of succeeding circle stimuli, while maintaining fixation on a central target. When a target stimulus appeared (a circle of a matching colour), subjects were required to release a touch bar in order to receive a fluid reward. Fluid control was within levels that do not negatively affect physiological or psychological welfare^63^. Eye position was monitored using an infrared video tracking system (Dalsa CCD camera [model SIM-0002] and an eye-tracking software from Thomas Recording ET-49 [Version 1.2.8]). This initial training allowed subjects to familiarise themselves with the experimental set-up and the timing structure of the task; this task was otherwise unrelated to the contrast discrimination experiment described below.

### Electrode array implantation

For surgical preparation, animals were sedated with ketamine. During surgery, anaesthesia and analgesia were maintained by sevoflurane (gaseous, 1–3%) and alfentanil (intravenous 156 μg/kg/h), respectively. Blood pressure, rectal temperature, blood oxygen saturation and end tidal CO_2_ were measured continuously. After the surgery, analgesic (metacam 0.1/kg) and prophylactic antibiotics (cephorex 0.5 ml/kg) was given for 3–5 days.

During surgery, the animals were placed in a stereotaxic head holder and the skull overlying the occipital and posterior temporal cortices was exposed. A craniotomy was made to remove the bone overlying V1, V2 and dorsal V4, using a pneumatic drill. The bone was kept in sterile 0.9% NaCl for refitting at the end of the surgery. The dura was opened to allow access to V4. Microelectrode chronic Utah arrays, attached to a CerePort™ base (Blackrock® Microsystems, connection dimensions of 16.5 mm [height] × 19 mm [base diameter] × 11 mm [body diameter]), were implanted under sterile conditions in the cortex, using a Blackrock microarray inserter. In monkey 1, two 4 × 5 grids of microelectrodes were implanted in area V4; in monkey 2, a 5 × 5 grid was implanted in V4. Electrodes were 1 mm in length, and their tips reached depths of up to 1 mm. Wire bundles were held in place with biologically compatible glue (histoacrylic), and the connector (CerePort™) was secured to the skull with titanium bone screws. Following array insertion, the Dura was re-sutured over the array, the exposed area was thinly covered with sterile Tisseel Lyo two-component fibrin sealant (Baxter Healthcare), and the bone flap was reinserted into the skull (before the Tisseel had fully set). The bone flap was cross bridged to the surrounding skull using Synthes orbital plate fragments and Synthes titanium bone screws.

The electrode arrays were inserted under visual control into the gyrus between the lunate sulcus and the superior temporal sulcus. The recording locations were confirmed to be in area V4 in both animals via visual inspection immediately postmortem and by analysis of postmortem Nissl-stained brain sections.

### Apparatus

Stimulus presentation was controlled using the CORTEX software (Laboratory of Neuropsychology, NIMH, http://dally.nimh.nih.gov/index.html) on a computer with an Intel® Core™ i3-540 processor. Stimuli were displayed at a viewing distance of 0.54 m, on a 25” Sony Trinitron CRT monitor with a resolution of 1280 by 1024 pixels, yielding a resolution of 31.5 pixels/degree of visual angle (dva). The monitor refresh rate was 85 Hz for monkey 1 and 75 Hz for monkey 2. The output of the red and green guns was combined using a Pelli-Zhang video attenuator, yielding a luminance resolution of 12 bits/pixel, allowing the presentation of contrasts that were well below contrast discrimination thresholds^64^. A gamma correction was used to linearize the monitor output.

### Data acquisition and processing

Raw data were acquired at a sampling frequency of 32,556 Hz with a 24-bit analogue-to-digital converter, with minimum and maximum input ranges of 11 and 136,986 microvolts, respectively (pre-set by Neuralynx, Inc.), a DMA buffer count of 128 and a DMA buffer size of 10 ms, using a 64-channel Digital Lynx 16SX Data Acquisition System (Neuralynx, Inc.). Digital referencing of voltage signals was performed prior to the recording of raw data, using commercially provided Cheetah 5 Data Acquisition Software v. 5.4.0 (Neuralynx, Inc.), to yield good SNRs for each channel.

Following each recording session, the raw data were processed offline using both commercial (Neuralynx, Inc.) and custom-written (Matlab, Mathworks) software. Signals were extracted using the Cheetah 5 Data Acquisition Software. The sampling frequency remained the same (32,556 Hz), while the bandpass filter frequency and the input range settings were individually tailored to each channel. Raw data were bandpass filtered with a low cut frequency of 600 Hz and a high cut frequency of 4000 Hz and saved at 16-bit resolution. This stage of processing generated ‘continuous MUA’ data, which was further processed to yield ‘spiking MUA’.

### Spiking MUA

An iterative procedure was carried out on the continuous MUA signal for each channel, in which the threshold for spike extraction was varied according to a staircase procedure, in order to yield levels of spontaneous spiking MUA (before the onset of the sample stimulus) that were similar (within 1% of a ‘target’ level) across sessions. To set the target level for each channel, the threshold was initially selected manually for all channels and sessions, and a ‘representative’ session was selected for each channel (i.e. a session with an ‘average’ SNR [see below for description] for that channel). Hence, the extraction of spiking MUA was performed such that spontaneous activity levels were standardized across recording sessions. As spontaneous activity levels were deliberately kept uniform across training days, we did (or could) not study whether spontaneous activity levels changed during training. What this method did allow, however, was the rigorous comparison of levels of stimulus-evoked activity across the training period, relative to spontaneous levels. For an example and additional details, see Supplementary Materials and Supplementary Figure 4.

### RF characterization

RFs were mapped using a reverse correlation procedure^65^ for each recording channel prior to training and recording. Additionally, orientation and spatial frequency tuning was determined using a reverse correlation procedure^65^. RF locations and tuning preferences were highly consistent across the training period as determined by regular remapping while learning commenced (every 3–5 days).

### Behavioural task

Each monkey was trained in a contrast discrimination task in which he differentiated between two successively presented stationary Gabor gratings based on their relative contrasts (Supplementary Figure 1).

Monkeys were initially trained on a very basic version of the contrast discrimination task at a location in the upper visual field, i.e. at a substantial distance from the RFs covered by our electrodes, which were located in the lower left visual field (for details, see below). When the animal understood the main concept of the task in the upper visual field, the stimuli were shifted to the left lower visual field. The stimuli (Gabor gratings, *σ* = 4°, spatial frequency = 2 cycle per degree, orientation = 90° vertical) were initially presented at an azimuth of −5° and an elevation of −16° in both monkeys (left and bottom compared to the fixation point). These coordinates covered the V4 RFs (Supplementary Figure 5).

Each trial was initiated when the monkey held a touch bar and fixated on a small fixation spot (diameter = 0.1°, fixation window = 2° × 2°) which was presented on a grey background (52.17 cd/m^2^). After 539 ms of fixation, a vertically oriented Gabor stimulus of 30% contrast, centred at the V4 RF coordinates, was presented for 512 ms. The outer diameter of the Gabor stimulus was truncated at 16° in monkey 1 and at 14° in monkey 2. Thereafter, a 512 ms inter-stimulus interval in monkey 2 or a randomly selected inter-stimulus interval from 512 to 1024 ms in monkey 1 followed, with only the fixation point present. After that, a test stimulus was presented for 512 ms. The test was identical in size and orientation to the sample stimulus but differed in contrast (5% or 90% contrast), which was chosen pseudo-randomly. Following test offset, another blank period of 512 ms with only the fixation point present occurred. Finally, the fixation point disappeared (cueing the monkey to make a saccade) and two target squares (one black, one white, size = 0.5°) appeared to the left and right of the location at which the sample and the test had been presented. The monkeys had to make a saccade to the white square (within a 2° × 2° window) if the test stimulus had a higher contrast than the sample stimulus and to the black square if the test stimulus had a lower contrast than the sample. A correct saccade resulted in a fluid reward, while an incorrect saccade resulted in no reward and a 0.2 s timeout. During the trial, if the monkey broke fixation before saccade cue onset or failed to respond within 1000 ms of the onset of the saccade cue, the trial was terminated immediately and followed by a 0.2 s timeout. We used different inter-stimulus intervals in the two animals for the following reason. We started training and recording in monkey 1, before doing so in monkey 2. We initially reasoned that a variable test onset would increase the animal’s focus and thereby possibly learning. In monkey 2, the variable onset during the very basic training resulted in too many early trial abortions, which quickly vanished when we used a fixed delay. We therefore decided to use a fixed delay in that animal.

After monkeys performed well in this easy version of the task, the number of test contrasts was increased to 8 (5, 10, 20, 25, 35, 40, 60 and 90% contrast, on day 1 of the proper contrast discrimination task), then to 12 (10, 15, 20, 25, 27, 29, 31, 33, 35, 40, 50 and 60% contrast, on day 2 of the proper contrast discrimination task) and to 14 (10, 15, 20, 25, 27, 28, 29, 31, 32, 33, 35, 40, 50 or 60% contrast, from day 3 of the proper contrast discrimination task). In order to motivate subjects to complete each trial and discourage them from guessing on difficult trials, stimulus drumming was carried out using the ‘repetition with delay’ function on CORTEX following error trials, i.e. enforcing the repeated presentation of a stimulus condition, until a minimum number of correct trials is accrued. Recording began simultaneously with the first day of training on the proper contrast discrimination task, but data analysis for the purpose of this paper was only performed from day 3 onwards, as this was the start of presenting the full range of contrasts.

### Data exclusion

The SNR was calculated for each channel on each day. The SNR was calculated as:

1 $${\mathrm{SNR}} = {\frac{\mathrm{Mean}_{{\mathrm{stimulus}}\,{\mathrm{activity}}} - {\mathrm{Mean}_{\mathrm{spontaneous}}}}{{\mathrm{SD}_{{\mathrm{spontaneous}}}}}}$$

whereby the mean stimulus activity was obtained from 150 to 250 ms after test onset, while the mean spontaneous activity was obtained during the 300-ms period before test onset. SD is the standard deviation of the mean response. This was calculated for each test contrast condition, yielding 14 SNR values per recording session for a given channel. Trials were included regardless of whether the subject’s response was correct. The size of the SNR varied depending on the test contrast. The highest of the 14 SNR values was then taken as being representative of the signal quality from a given channel for each session.

Channels were included in the individual channel analyses if they had daily SNR ≥ 1, on at least 80% of the total number of recording days.

### Neuronal data analysis

The results reported in this paper are based on the analysis of spiking MUA. A parallel analysis was carried out using envelope MUA^66^ and single unit analysis (Supplementary note 9, Supplementary Figure 12-20), which yielded qualitatively similar results. The number of trials obtained across the different recording session for the different test contrasts are given in Supplementary Table 1.

### Determination of the analysis time window

The study aimed to determine how well neural activity encoded the stimuli (i.e. to quantify NSD) and to quantify how well neural activity reflected (predicted) choice (i.e. CP). As stated, both sample and test stimuli were presented for 512 ms (each). However, no a priori information justifies the selection of the entire intervals to investigate NSD or CP, as the relationship between neural activity and stimulus or choice may vary during the stimulus presentation period, due to, for example, onset-induced response transients. To assess whether NSD or CP varied during the response periods, we performed an AUROC 'ideal observer' discrimination analysis (equal to that described in later Results sections). For this analysis, we employed sliding time windows over the test period and varied parametrically the window length (window sizes of 50–250 ms, in steps of 5–20 ms). To avoid biasing the assessment of how learning affects the discriminability of single channels, we used the summed activity from all channels for this analysis. Furthermore, to avoid biasing comparisons between early or late sessions, results from these exploratory analyses were considered only after averaging across all experimental sessions, without any distinction between early or late sessions. We found that the NSD and CP varied over the 512-ms interval in both animals, decaying in the late part of the interval. Furthermore, the period of maximal NSD and CP differed between the two animals. In monkey 1, maximal NSD and CP values occurred shortly after stimulus onset, while in monkey 2 it was delayed by ~128 ms relative to monkey 1. Thus, in monkey 1 the response transient contained most of the test stimulus information, while in monkey 2 the sustained response period contained most of the test stimulus information. To account for these differences and for the decay towards the end of the interval, we selected time windows of half the length of the 512 ms stimulus presentation intervals for all quantitative analyses reported in this paper (i.e. an interval of 256 ms, starting at 30 ms after stimulus onset in monkey 1 and at 158 ms after stimulus onset in monkey 2). However, to confirm that the selection criterion used did not bias the results, we additionally performed all reported analyses using the entire response period (30–542 ms after stimulus onset). This control analysis yielded qualitatively identical results, albeit with smaller overall effects due to the inclusion of uninformative response periods. We additionally determined the response window based on ideal observer discrimination analysis for each channel individually and then averaged the AUROCs across channels. This approach yielded the same time windows as the one where activity was pooled before performing the AUROC analysis (Supplementary Materials and Supplementary Figure 2, 3 for additional information).

### Contrast response functions

To investigate the changes in the CRF with training, contrast-dependent firing rates during the selected time window of the test presentation period were calculated for each channel, and a Naka–Rushton function was fitted using the method of least squares, according to the formula:

2 $$R = R_{{\mathrm{max}}}\frac{{C^n}}{{C^n + C_{50}^n}} + M$$

where *R* refers to the observed firing rate in spikes per second; *R*_max_ is the maximum response level; the *C*_50_ is the contrast at which the response elicited was 50% of the maximum; *n* controls the slope of the curve; and *M* is the level of spontaneous activity^24,67^. To identify changes in the properties of the CRF, four parameters (the slope of the function at 30% contrast, the *C*_50_ and the minimum (*M*) and maximum responses (Rmax)) were calculated for each session and channel and a Spearman’s correlation was calculated between the parameter values and session number. The slope at 30% contrast was calculated as:

3 $${\mathrm{Slope}} = \frac{{{\mathrm{d}}R}}{{{\mathrm{d}}C}} = nR_{\mathrm{max}}\frac{{C^{n - 1}C_{50}^n}}{{\left( {C^n + C_{50}^n} \right)^2}}$$

### Accounting for the effect of trial-to-trial activity fluctuations on discriminability and decision-related neuronal measures: a COBE

A common way to quantify neuronal discriminability has been to calculate the performance of an ideal observer who discriminates between stimuli that vary along an ordinal scale (e.g. the contrast or orientation of gratings or frequency of flutters in somatosensation). The underlying assumption is that that neuronal response differences are consistent with the stimulus differences. For example, given two stimuli with features *s*_1_ and *s*_2_ such that *s*_2_ > *s*_1_, which elicit responses *r*_1_ and *r*_2_, the ideal observer associates *s*_2_ with the higher response, and hence its decoding performance is quantified by the probability *p* (*r*_2_ > *r*_1_). A traditional AUROC analysis estimates this probability based on the assumption that *r*_1_ and *r*_2_ are independently sampled from their distributions on every trial. However, in the case of 2-AFC tasks in which the two stimuli are presented consecutively within a short period of time, such as within one trial, within-trial autocorrelations (such as, for example, state-dependent gain fluctuations), lead to response co-variations. Neglecting this within-trial autocorrelation of *r*_1_ and *r*_2_ can lead to underestimates regarding the ability to discriminate *s*_1_ and *s*_2_ (Supplementary Figure 23). Here we use a simple nonparametric alternative to the AUROC estimator we name COBE (see Supplementary Note 11), which takes these co-variations into account.

### Neurometric functions

To generate neurometric functions, the AUROC (or COBE) data from each day were fitted with a four-parameter Weibull function using maximum likelihood estimation, according to the formula:

4 $$y = 1 - \delta - \left( {\gamma e^{ - \left( {\frac{x}{\alpha }} \right)^\beta }} \right)$$

where *y* is the AUROC value; *x* is the contrast of the test stimulus; the scale *α* is the contrast at which the neurometric function is at 63% of its range; the shape exponent *β* modulates the slope at threshold; *γ* is the range; and 1−*δ* is the maximum AUROC value reached by the neurometric function.

We calculated the slope at 30% contrast as:

5 $${\mathrm{Slope}\,{\mathrm{at}\,{30}}}{\% } = \frac{{{\mathrm{d}}\left[1 - \delta - \left( {\gamma {e}^{ - \left( {\frac{x}{\alpha }} \right)^\beta }} \right)\right]}}{{{\mathrm{d}}{x}}} = \left[\frac{{\beta \gamma (\frac{{30}}{\alpha })^\beta {e}^{ - (\frac{{30}}{\alpha })^{\beta}}}}{{30}}\right]$$

We also determined the PNE for each channel and training day, i.e. the point where the channel activity is unable to distinguish between sample and the test contrasts responses (AUROC = 0.5). During a subset of sessions for some channels, the range spanned by the AUROC values did not include the value of 0.5 (i.e. the fitted neurometric curve was located entirely within either the upper or lower half of the range spanned by the *y* axis), thus the PNE could not be calculated for these sessions. Channels were included in the PNE analysis if the PNE could be calculated on at least 80% of sessions, resulting in the inclusion of 21/29 channels from monkey 1 and 15/20 channels from monkey 2 (note that this exclusion was not applied for the other analyses). On days for which PNEs could not be calculated for a specific session, the averages were calculated across those channels for which PNEs could be calculated.

### Calculation of *C*_50_ and PNE changes at the population level

We encountered a few channels (*n* = 3, monkey 1; *n* = 0, monkey 2) where spiking activity decreased with increasing contrast consistently across recording/training days (for an example, see Supplementary Figure 6). These channels received the label ‘reversed tuning’. Channels were defined as such if their average slope of the Naka–Rushton function (averaged across all training days) was negative. These channels should theoretically show a decrease of the slope at 30% of the tuning function with learning (becoming more negative), rather than the increase that was predicted for the other channels. To account for this difference in prediction, we multiplied their slope value (of the Naka–Rushton function and of the neurometric function) by −1. This approach allowed to average their slope (and changes thereof) with the slope values obtained from the more typical channels.

On some channels, the *C*_50_/PNE was >30% at the start of learning, and in these channels *C*_50_/PNE generally decreased towards 30% during learning. On other channels, the *C*_50_/PNE was <30% at the start of learning, and in these channels it generally increased towards 30% during learning. To examine whether parameters such as the *C*_50_ and the PNE changed with learning at the population level, we calculated the absolute value of the difference between the *C*_50_ and 30% contrast, and the absolute value of the difference between PNE and 30% contrast. By using the absolute value of the difference, we were able to combine the two groups of channels (those with *C*_50_/PNE > 30% at the start of learning and those with *C*_50_/PNE < 30% at the start of learning) and investigate whether *C*_50/_PNEs shifted systematically towards the sample contrast with learning, irrespective of their starting position.

### Sample–test discriminability

To analyse how well channels discriminated between sample and test stimuli, we calculated AUROC values for each sample–test contrast pair and determined whether these systematically changed with learning. Specifically we would expect the AUROC values for test contrasts that were higher than the sample stimulus to increase with learning and for those that were lower than the sample contrast to decrease with learning. This expectation holds for channels with typical contrast tuning (i.e. increasing firing rates with increasing contrast) but would be reversed for channels with the label ‘reversed tuning’ (see above). To account for this difference in prediction, the AUROC values for the three channels with reversed tuning were calculated as the probability that the test contrast had lower activity than the sample contrast, rather than the probability that the test contrast had higher contrast than the sample contrast.

### Test–test discriminability

In addition to changes in discriminability between sample and test stimuli, we assessed how test–test discriminability changed with training. This required the pooling of data across trials. Thus we estimate the probability that responses to a certain test stimulus are higher than to a different test stimulus only with the standard AUROC method (the COBE analysis is not applicable here). AUROC values were calculated based on comparisons of responses between 29% and 31%, between 28% and 32% and between 27% and 33% test contrast conditions, i.e. those contrasts that were most difficult to discriminate. AUROC values were then plotted as a function of session number. Data were pooled for the first 5 days of training and the last 5 days of training, and Wilcoxon signed rank test was performed to determine whether discriminability changed significantly with training. As before, predictions of how AUROC values should change with learning differed between channel with normal and those with reversed contrast tuning. We therefore calculated the AUROC values for channel with reversed tuning as 1 − AUROC.

### Choice probability

CPs were monitored over the course of training to assess the degree to which the neuronal activity reflected the identity of their chosen target. Levels of spiking activity for a given test stimulus were categorized according to whether the subject made a saccade to the black or to the white target, i.e. they were conditioned upon the monkey's choice. This yielded two activity distributions for each test stimulus. CPs were calculated between the two resulting groups of activity as the AUROC, which is generally referred to as CP. This was done for the challenging test contrast conditions (27, 28, 29, 31, 32 and 33%). For each channel, the mean CP (for a given test contrast) was calculated for early and late sessions (the first and last 5 days of training, respectively). CP values for channels with ‘reversed tuning’ were calculated as 1 − CP, for reasons outlined previously. A mixed model two-way repeated-measures (RM) ANOVA was performed to determine whether CPs changed significantly with training days (early vs. late sessions, factor 1) and test contrast (factor 2). In addition, for each of the different test contrasts, a post hoc one-sided *t* test was performed to determine whether the means of the two distributions differed significantly. A one-sided test was used as we were interested solely in whether neuronal activity became *more* indicative of the monkeys’ upcoming choice during the final stages of training. However, a two-sided test yielded qualitatively identical results.

To assess whether differences in responses between sample and test stimuli became more indicative of the animal’s behavioural response, we also calculated CPs for activity evoked by the test minus activity evoked by the sample. This approach performs the differentiation for within-trial activity, aiming to remove slow activity fluctuations from the data. The assumption is that the animals potentially base their decisions on activity differences between sample and test within trials, rather than absolute activity levels arising from test stimulus presentation. In that sense, the approach is similar to the COBE approach, while nevertheless calculating AUROC values based on activity distributions.

### Noise correlation analysis

Noise correlations were calculated separately for each recording channel pair, stimulus contrast and recording day. To do this, we calculated the correlation of firing rates given a specific stimulus on each training day. Noise correlation values were Fisher *z*-transformed and then averaged across the first 5 days of training and the last 5 days of training (separately for each channel pair and for each test contrast). To determine whether noise correlations changed with learning, we performed a mixed model two-factor RM ANOVA, with contrast and training period as main factors.

### Fisher information analysis

We used a recently published method and algorithms^68^ to calculate the Fisher information in single channels and in populations of simultaneously recorded channels^32^. We estimated the information present when comparing 29–31% contrast, 28–32% contrast, 27–33% contrast, etc. The derivative to calculate the Fisher information for e.g. 29–31% contrast is thus delta = 2% contrast (see refs. ^32,68^ for details). For 28–32% contrast, the delta = 4% contrast (and so on forth). This is analogous to the methods described by Kanitscheider et al.^32,68^, but it is converted from the orientation domain to the contrast domain. In the orientation domain used by Kanitscheider et al.^32,68^, the Fisher information was scaled by the orientation difference (maxD = pi). We have used an analogous system where we assume that 50% contrast difference is equal to maxD = pi, i.e. a 2% contrast difference would equate to (pi/50) × 2. Note that, even if this conversion is not equivalent as contrast data are not circular (while orientation data are), it does not affect the conclusions from our study. This is because absolute values of information were of little interest here, of interest was whether learning alters the information encoded for a fixed contrast difference. To calculate the information a given channel (or channel population) encoded in the first 5 days (or last 5 days) of training, the trials from a given channel and a given contrast pair of all 5 days were concatenated as if they had been recorded in a single session. We included trials with correct decisions in this analysis. The analysis requires equal trial numbers for the two stimulus comparisons, which were not guaranteed, due to the fact that the animal stopped working on individual days at unpredictable times. We therefore used the lower number of trials available for a given test contrast pair on a given training day and truncated the trials available for the other stimulus contrast at that lower number for that training day. This approach yielded between 215 (minimum) and 469 (maximum) trials for each channel, test contrast comparison and monkey (monkey 1: *n* = 293–469; monkey 2: *n* = 215–385).

The information encoded by differently sized (neuronal) populations was calculated by using the above described approach to concatenate the trials from different recording channel for each channel and then calculate the information in a population of size *x* (i.e. number of channels) with channel and trial identity retained. To identify to what extent correlated activity reduced the information present in a population, we calculated the activity when trials were shuffled, using the algorithms provided by ref. ^32^.

### Significance of noise vs. signal correlation regression slope changes

We performed a permutation test to determine whether the slopes found for the late period were significantly different from the slopes during the early period for our pairs with positive signal correlations. To do so, we joined the early and late distributions of the signal and of the noise correlations for the respective channel samples (separated according to their information content, see Results). We then drew 1000 random samples (with a sample size that equalled the sample size for the late distributions) from that joint distribution and calculated the slope for each of these. If the original slope from the late training period fell outside the 95% range of the slopes from the joint distributions, it was deemed significantly different to the slope from the early distribution.

### Code availability

Data were processed with Neuralynx and custom-written Matlab code, which is stored on secure servers and which can be made available upon reasonable request.

## Electronic supplementary material

Supplementary Information

## Data Availability

Original data stored on Newcastle University servers can be made available upon reasonable request.

## References

[1] Yu Q, Zhang P, Qiu J, Fang F (2016). Perceptual learning of contrast detection in the human lateral geniculate nucleus. Curr. Biol..

[2] Ito M, Westheimer G, Gilbert CD (1998). Attention and perceptual learning modulate contextual influences on visual perception. Neuron.

[3] Schoups A, Vogels R, Qian N, Orban G (2001). Practising orientation identification improves orientation coding in V1 neurons. Nature.

[4] Crist RE, Li W, Gilbert CD (2001). Learning to see: experience and attention in primary visual cortex. Nat. Neurosci..

[5] Ghose GM, Yang T, Maunsell JH (2002). Physiological correlates of perceptual learning in monkey V1 and V2. J. Neurophysiol..

[6] Li W, Piech V, Gilbert CD (2004). Perceptual learning and top-down influences in primary visual cortex. Nat. Neurosci..

[7] Thiele A (2004). Perceptual learning: is V1 up to the task?. Curr. Biol..

[8] Yan Y (2014). Perceptual training continuously refines neuronal population codes in primary visual cortex. Nat. Neurosci..

[9] Yang T, Maunsell JH (2004). The effect of perceptual learning on neuronal responses in monkey visual area V4. J. Neurosci..

[10] Raiguel S, Vogels R, Mysore SG, Orban GA (2006). Learning to see the difference specifically alters the most informative V4 neurons. J. Neurosci..

[11] Adab HZ, Vogels R (2011). Practicing coarse orientation discrimination improves orientation signals in macaque cortical area v4. Curr. Biol..

[12] Adab HZ, Popivanov ID, Vanduffel W, Vogels R (2014). Perceptual learning of simple stimuli modifies stimulus representations in posterior inferior temporal cortex. J. Cogn. Neurosci..

[13] Law CT, Gold JI (2008). Neural correlates of perceptual learning in a sensory-motor, but not a sensory, cortical area. Nat. Neurosci..

[14] Freedman DJ, Assad JA (2006). Experience-dependent representation of visual categories in parietal cortex. Nature.

[15] Uka T, Sasaki R, Kumano H (2012). Change in choice-related response modulation in area MT during learning of a depth-discrimination task is consistent with task learning. J. Neurosci..

[16] Gu Y (2011). Perceptual learning reduces interneuronal correlations in macaque visual cortex. Neuron.

[17] Chen X, Sanayei M, Thiele A (2013). Perceptual learning of contrast discrimination in *Macaca mulatta*. J. Vis..

[18] Chen X, Sanayei M, Thiele A (2014). Stimulus roving and flankers affect perceptual learning of contrast discrimination in *Macaca mulatta*. PLoS ONE.

[19] Brady N, Field DJ (2000). Local contrast in natural images: normalisation and coding efficiency. Perception.

[20] Adini Y, Wilkonsky A, Haspel R, Tsodyks M, Sagi D (2004). Perceptual learning in contrast discrimination: the effect of contrast uncertainty. J. Vis..

[21] Yu C, Klein SA, Levi DM (2004). Perceptual learning in contrast discrimination and the (minimal) role of context. J. Vis..

[22] Ohazawa I, Sclar G, Freeman RD (1985). Contrast gain control in the cat’s visual system. J. Neurophysiol..

[23] Tolhurst DJ, Movshon JA, Dean AF (1983). The statistical reliability of signals in single neurons in cat and monkey visual cortex. Vis. Res..

[24] Sclar G, Maunsell JH, Lennie P (1990). Coding of image contrast in central visual pathways of the macaque monkey. Vis. Res..

[25] Levitt JB, Kiper DC, Movshon JA (1994). Receptive fields and functional architecture of macaque V2. J. Neurophysiol..

[26] Sceniak MP, Ringach DL, Hawken MJ, Shapley R (1999). Contrast’s effect on spatial summation by macaque V1 neurons. Nat. Neurosci..

[27] Thiele A, Dobkins KR, Albright TD (2000). Neural correlates of contrast detection at threshold. Neuron.

[28] Williford T, Maunsell JH (2006). Effects of spatial attention on contrast response functions in macaque area V4. J. Neurophysiol..

[29] Pooresmaeili A, Poort J, Thiele A, Roelfsema PR (2010). Separable codes for attention and luminance contrast in the primary visual cortex. J. Neurosci..

[30] Sani I, Santandrea E, Golzar A, Morrone MC, Chelazzi L (2013). Selective tuning for contrast in macaque area V4. J. Neurosci..

[31] Goris RL, Movshon JA, Simoncelli EP (2014). Partitioning neuronal variability. Nat. Neurosci..

[32] Kanitscheider I, Coen-Cagli R, Kohn A, Pouget A (2015). Measuring Fisher information accurately in correlated neural populations. PLoS Comput. Biol..

[33] Ince RA, Panzeri S, Kayser C (2013). Neural codes formed by small and temporally precise populations in auditory cortex. J. Neurosci..

[34] Panzeri S, Schultz SR, Treves A, Rolls ET (1999). Correlations and the encoding of information in the nervous system. Proc. R. Soc. Lond. B Biol. Sci..

[35] Abbott LF, Dayan P (1999). The effect of correlated variability on the accuracy of a population code. Neural Comput..

[36] Averbeck BB, Latham PE, Pouget A (2006). Neural correlations, population coding and computation. Nat. Rev. Neurosci..

[37] Oram MW, Foldiak P, Perrett DI, Sengpiel F (1998). The ‘Ideal Homunculus’: decoding neural population signals. Trends Neurosci..

[38] Pola G, Thiele A, Hoffmann KP, Panzeri S (2003). An exact method to quantify the information transmitted by different mechanisms of correlational coding. Network.

[39] Minces V, Pinto L, Dan Y, Chiba AA (2017). Cholinergic shaping of neural correlations. Proc. Natl. Acad. Sci. USA.

[40] Hua T (2010). Perceptual learning improves contrast sensitivity of V1 neurons in cats. Curr. Biol..

[41] Li W, Piech V, Gilbert CD (2008). Learning to link visual contours. Neuron.

[42] Panzeri S, Harvey CD, Piasini E, Latham PE, Fellin T (2017). Cracking the neural code for sensory perception by combining statistics, intervention, and behavior. Neuron.

[43] Shadlen MN, Britten KH, Newsome WT, Movshon JA (1996). A computational analysis of the relationship between neuronal and behavioral responses to visual motion. J. Neurosci..

[44] Nienborg H, Cumming BG (2009). Decision-related activity in sensory neurons reflects more than a neuron’s causal effect. Nature.

[45] Roelfsema PR, Spekreijse H (2001). The representation of erroneously perceived stimuli in the primary visual cortex. Neuron.

[46] Nienborg H, Cohen MR, Cumming BG (2012). Decision-related activity in sensory neurons: correlations among neurons and with behavior. Annu. Rev. Neurosci..

[47] Kumano H, Uka T (2013). Neuronal mechanisms of visual perceptual learning. Behav. Brain Res..

[48] Gu, Y., Angelaki, D. E. & DeAngelis, G. C. Contribution of correlated noise and selective decoding to choice probability measurements in extrastriate visual cortex. *eLife* **3**, 10.7554/eLife.02670 (2014). 10.7554/eLife.02670 PMC4109308 24986734

[49] Ni AM, Ruff DA, Alberts JJ, Symmonds J, Cohen MR (2018). Learning and attention reveal a general relationship between population activity and behavior. Science.

[50] van Kempen, J., Panzeri, S. & Thiele, A. Cholinergic control of information coding. *Trends Neurosci*. **40**, 522–524 (2017). 10.1016/j.tins.2017.06.006 28693847

[51] Bondy AG, Haefner RM, Cumming BG (2018). Feedback determines the structure of correlated variability in primary visual cortex. Nat. Neurosci..

[52] Cohen MR, Maunsell JH (2009). Attention improves performance primarily by reducing interneuronal correlations. Nat. Neurosci..

[53] Ruff DA, Cohen MR (2014). Attention can either increase or decrease spike count correlations in visual cortex. Nat. Neurosci..

[54] Rabinowitz, N. C., Goris, R. L., Cohen, M. & Simoncelli, E. Attention stabilizes the shared gain of V4 populations. *eLife* **4**, e08998 (2015). 10.7554/eLife.08998 PMC4758958 26523390

[55] Herrero JL, Gieselmann MA, Sanayei M, Thiele A (2013). Attention-induced variance and noise correlation reduction in macaque V1 is mediated by NMDA receptors. Neuron.

[56] Thiele A (2016). Attention induced gain stabilization in broad and narrow-spiking cells in the frontal eye-field of macaque monkeys. J. Neurosci..

[57] Mitchell JF, Sundberg KA, Reynolds JH (2009). Spatial attention decorrelates intrinsic activity fluctuations in macaque area V4. Neuron.

[58] Herrero JL (2008). Acetylcholine contributes through muscarinic receptors to attentional modulation in V1. Nature.

[59] Bakin JS, Weinberger NM (1996). Induction of a physiological memory in the cerebral cortex by stimulation of the nucleus basalis. Proc. Natl. Acad. Sci. USA.

[60] Dimyan MA, Weinberger NM (1999). Basal forebrain stimulation induces discriminative receptive field plasticity in the auditory cortex. Behav. Neurosci..

[61] Thiele A, Herrero JL, Distler C, Hoffmann KP (2012). Contribution of cholinergic and GABAergic mechanisms to direction tuning, discriminability, response reliability, and neuronal rate correlations in macaque middle temporal area. J. Neurosci..

[62] Thiele A, Delicato LS, Roberts MJ, Gieselmann MA (2006). A novel electrode-pipette design for simultaneous recording of extracellular spikes and iontophoretic drug application in awake behaving monkeys. J. Neurosci. Methods.

[63] Gray, H. et al. Physiological, behavioral, and scientific impact of different fluid control protocols in the rhesus macaque (*Macaca mulatta*). *eNeuro* **3**, ENEURO.0195-16.2016 (2016). 10.1523/ENEURO.0195-16.2016 PMC5032891 27679812

[64] Pelli D, Zhang L (1991). Accurate control of contrast on microcomputer displays. Vis. Res..

[65] Gieselmann MA, Thiele A (2008). Comparison of spatial integration and surround suppression characteristics in spiking activity and the local field potential in macaque V1. Eur. J. Neurosci..

[66] Super H, Roelfsema PR (2005). Chronic multiunit recordings in behaving animals: advantages and limitations. Prog. Brain Res..

[67] Albrecht DG, Hamilton DB (1982). Striate cortex of monkey and cat: contrast response function. J. Neurophysiol..

[68] Kanitscheider, I., Coen-Cagli, R., Kohn, A. & Pouget, A. MatLab tools for estimating linear Fisher information from population data along with synthetic data and recorded spike count responses from neurons in macaque primary visual cortex to grating images with different orientations and white noise. CRCNS.org. https://doi.org/10.6080.K0PK0D3B (2015).

