# Supplementary material for: Perceptual learning of fine contrast discrimination changes neuronal tuning and population coding in macaque V4
Source: Nat Commun. 2018 Oct 12;9:4238. doi: 10.1038/s41467-018-06698-w (PMC6185947; doi:10.1038/s41467-018-06698-w)
Supplement: Supplementary file 1 — Supplementary Information [file 41467_2018_6698_MOESM1_ESM.pdf]

Supplementary Materials: Perceptual learning of fine contrast discrimination changes neuronal tuning and population coding in macaque V4

Mehdi Sanayei[1,5], Xing Chen[1,5], Daniel Chicharro[2,3], Claudia Distler[4], Stefano Panzeri[2,6], Alexander Thiele[1,6,*]

[1] Institute of Neuroscience, Newcastle University, Framlington Place, Newcastle upon Tyne, UK, NE2 4HH; [2] Laboratory of Neural Computation, Istituto Italiano di Tecnologia, 38068 Rovereto, Italy; 3 Department of Neurobiology, Harvard Medical School, Boston, MA, 02115, USA; [4]Allgemeine Zoologie und Neurobiologie, Ruhr-Universität Bochum, 44801 Germany; [5] These authors contributed equally to this work; [6] Senior authors;

*Corresponding author: Alexander Thiele, alex.thiele@ncl.ac.uk

Acknowledgements: The research was supported by the Medical Research Council, UK, G0700976, the Wellcome Trust, and by the Autonomous Province of Trento ("Grandi Progetti 2012," "ATTEND"). DC was supported by the Fondation Bertarelli. The authors would like to thank the Comparative Biology Centre staff at Newcastle University for their excellent technical support.

Author contribution: study conception and planning: AT., data acquisition: M.S, X.C., A.T., histology and recording site verification: C.D., data analysis: A.T., M.S., X.C., D.C., theory and computational modelling: D.C., S.P., manuscript writing: A.T., S.P., D.C., M.S., X.C., and C.D.

The authors declare no competing interests.

Supplementary Methods

Task overview

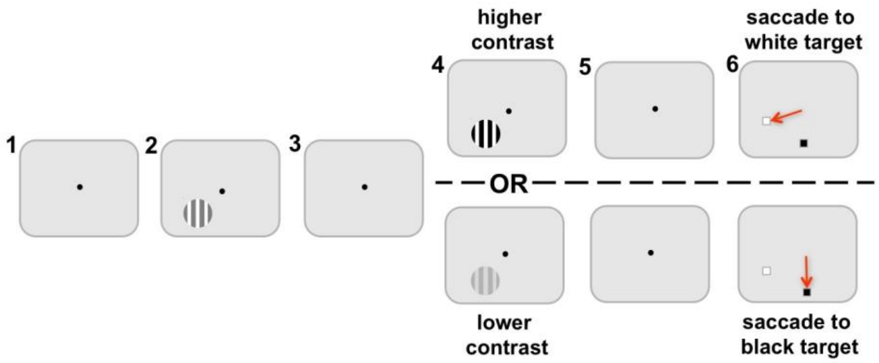

Supplementary Figure 1. *Illustration of the contrast discrimination task*. 1) The monkeys were required to fixate upon a central spot, to initiate the trial. 2) While maintaining fixation, a sample stimulus of 30% contrast (either a Gabor patch or a sinusoidal grating) was presented for 512 ms in the lower left visual field. 3) Presentation of the sample stimulus was followed by an interval lasting 512 ms (except during training at the peripheral location for monkey 1, where the interval lasted for a random duration of 512 to 1024 ms). 4) Next, the test stimulus (another Gabor patch or sinusoidal grating which could be of higher or lower contrast than the sample), was presented for 512 ms. 5) This was followed by a second interval of 400 ms. 6) Two target stimuli appeared to the left and right of the location at which the sample and test had previously been presented; the fixation spot changed colour from black to grey, signalling that the animals were allowed to make a saccade to their chosen target. If the test was of a higher contrast (e.g. 32%) than the sample (always 30%), the monkeys had to saccade to the white target; otherwise, if the test stimulus was of a lower contrast (e.g. 28%), they had to saccade to the black target. The red arrows in the figure indicate the direction of saccadic motion for illustrative purposes only; they did not appear onscreen. The figure has been published previously [1].

Determination of the analysis time window

As described in the main text, we examined how neuronal stimulus discriminability (NSD) and choice probability (CP) varied depending on the selection of the time window of the responses used to calculate them. For that purpose, we calculated the AUROC measures using sliding time windows of different length, spanning the test stimulus interval. The window size was explored in the range 50-250ms. The same analysis was carried out for each channel separately, to obtain an averaged AUROC across channels, or alternatively pooling the responses of all channels to calculate a single AUROC value from the pooled activity. In Supplementary Figure 2 we show the results obtained with a sliding window of 256ms, half the interval length, as a function of the window onset with respect to the start of the test stimulus interval. Results are consistent for the averaged AUROC across channels and the AUROC of the pooled channels. For monkey 1, both NSD and CP are higher after the stimulus onset and decay in the late part of the interval. For monkey 2, NSD and CP do not depend on the window location in early days and increase during the sustained response for late days. Given these differences between the two monkeys, we selected a window starting at 30ms after stimulus onset in monkey 1, and at 158 ms after stimulus onset in monkey 2 (see vertical lines in Supplementary Figure 2). The comparison of NSD and CP for early and late days indicates that the selection criterion did not bias the conclusions about the effect of learning. Furthermore, our results are robust to the selection of the window. This is further supported by Supplementary Figure 3, which shows how NSD and CP depend on the window length, starting from the test stimulus onset. The NSD and CP calculated from the whole interval are comparable to the ones obtained from the local 256ms specifically chosen for each monkey. Accordingly, qualitatively identical conclusions are obtained for all our analyses when using the whole interval. Moreover, the fact that NSD and CP from the whole interval are not higher than for the selected 256ms window further indicates that the neural responses of each monkey differ in the time scale for the discrimination of test stimuli and choices.

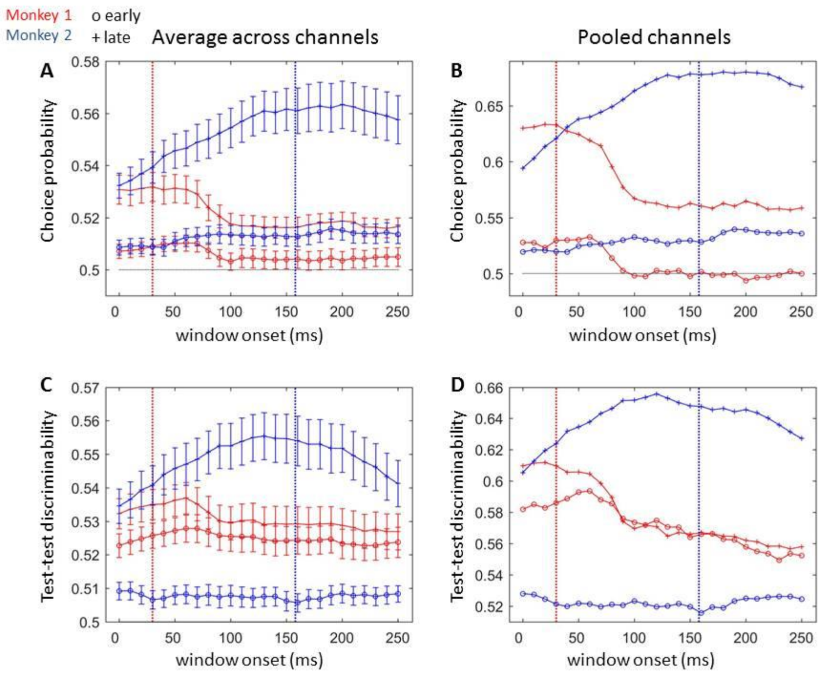

Supplementary Figure 2. ***Determination of the analysis time window.*** Choice probability and test-test discriminability calculated with responses during the test interval within a sliding window of 256 ms. Results are shown for the 5 early and late sessions separately, averaged across contrasts 27-33%. A) Choice probability averaged across channels. For channels with inverse tuning, 1-CP was used for the average. B) Choice probability calculated for the pooled activity combining all channels. C) Test-test discriminability averaged across channels. For channels with inverse tuning, 1-AUROC was used for the average. To average across contrasts, 1-AUROC was used for contrasts lower than 30%. Responses to contrast 29% were taken as reference for each test-test discriminability pair. D) Test-test discriminability for the pooled activity combining all channels. Vertical lines indicate the onset of the time window selected in the Results. Error bars indicate the standard deviation of the mean (n=29 and n=20 per data point for monkey 1 and 2 respectively).

.

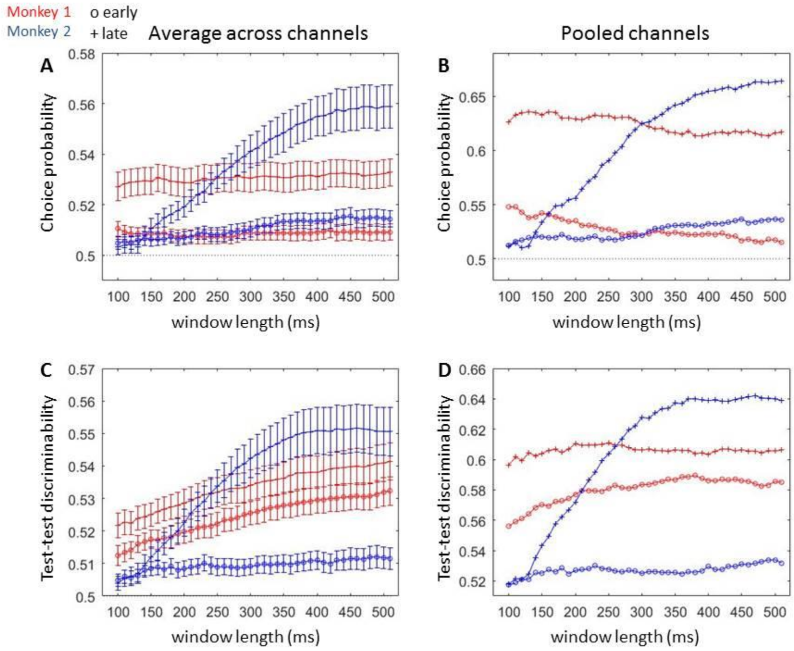

Supplementary Figure 3. ***Choice probability and neural discriminability as a function of window length.*** The measures are calculated with responses during the test interval within a window starting on the onset of the stimulus and with an increasing window length. Like in Supplementary Figure 2: Results are shown for the 5 early and late sessions separately, averaged across contrasts 27-33%. A) Choice probability averaged across channels. For channels with inverse tuning, 1-CP was used for the average. B) Choice probability calculated for the pooled activity combining all channels. C) Test-test discriminability averaged across channels. For channels with inverse tuning, 1-AUROC was used for the average. To average across contrasts, 1-AUROC was used for contrasts lower than 30%. Responses to contrast 29% were taken as reference for each test-test discriminability pair. D) Test-test discriminability for the pooled activity combining all channels. Error bars indicate the standard deviation of the mean. Error bars denote S.E.M. (n=29 and n=20 per data point for monkey 1 and 2 respectively).

Trial numbers for different conditions and sessions

	Session	10%	15%	20%	25%	27%	28%	29%	31%	32%	33%	35%	40%	50%	60%
Monkey 1	1	106	111	114	122	149	152	159	184	172	150	132	113	106	105
	2	24	24	23	31	38	40	44	30	32	33	30	26	23	22
	3	63	63	73	81	92	91	114	100	85	78	70	61	60	59
	4	36	34	34	40	55	49	60	75	44	42	42	32	32	33
	5	77	81	88	99	104	116	135	134	108	88	89	74	73	76
	6	61	64	67	70	93	91	95	117	90	80	69	63	66	65
	7	26	27	27	31	45	36	44	41	36	33	31	27	26	26
	8	22	20	21	28	35	38	39	24	25	31	24	20	20	19
	9	20	22	22	27	29	29	42	50	39	35	24	24	23	21
	10	72	70	72	75	91	92	101	144	109	97	76	73	65	72
	11	19	19	17	17	19	21	22	28	26	27	18	22	18	18
	12	81	77	78	103	114	122	139	122	121	97	82	77	79	77
	13	50	50	50	50	50	50	50	50	50	50	50	50	50	50
	14	29	32	28	32	34	46	41	60	49	43	33	31	30	26
	15	68	65	69	79	97	89	96	92	102	86	75	68	67	69
	16	110	112	117	134	160	159	191	168	150	128	122	113	113	112
	17	50	50	52	60	61	73	77	83	70	72	61	49	49	49
	18	70	68	68	82	98	106	113	109	90	80	76	69	67	71
	19	84	88	85	91	98	123	138	156	129	114	99	86	85	86
	20	72	73	68	88	90	107	97	113	103	108	82	74	72	71
	21	31	30	33	32	45	45	62	47	49	35	31	34	31	32
Monkey 2	1	60	59	51	64	66	62	54	63	69	75	101	65	49	43
	2	63	55	52	59	75	65	74	81	91	65	87	87	56	34
	3	56	71	73	47	81	74	83	73	67	87	104	79	60	51
	4	81	76	102	103	91	74	92	96	110	89	125	89	59	52
	5	77	77	71	64	77	75	84	90	82	96	79	76	47	46
	6	52	49	54	61	66	61	49	75	80	71	59	56	38	37
	7	39	33	43	30	53	47	52	40	58	61	48	42	33	31
	8	62	47	57	60	67	77	72	62	79	75	83	56	37	31
	9	48	51	58	74	60	76	75	56	71	63	60	50	44	45
	10	47	38	46	54	49	48	66	74	58	58	38	38	37	37
	11	48	48	56	57	79	90	86	101	78	57	57	54	45	47
	12	29	41	46	41	57	54	64	74	48	69	59	43	43	33
	13	44	48	58	53	53	66	77	83	78	70	64	54	48	47
	14	32	27	32	40	40	31	42	64	36	31	43	32	31	25
	15	31	35	31	42	43	41	44	52	52	48	36	37	38	36
	16	41	43	42	41	57	71	61	84	45	59	59	41	41	40
	17	30	40	33	39	58	46	68	70	56	54	44	39	40	33
	18	22	19	27	27	36	34	46	38	34	43	33	26	19	27
	19	44	39	47	50	55	60	80	83	61	73	50	44	37	37
	20	36	32	36	37	44	59	60	87	58	51	35	39	37	32
	21	32	45	32	47	31	49	55	48	43	55	42	30	29	29
	22	49	46	48	56	61	56	73	88	90	54	60	49	38	47
	23	62	70	64	72	74	78	99	117	96	95	75	59	62	56
	24	60	61	56	62	82	79	85	100	88	88	69	67	53	63
	25	59	53	47	71	63	79	82	97	73	79	64	58	61	40

Supplementary table 1. ***Number of trials per session for the two monkeys performed for a given test contrast***. Sum of correct and error trials are listed (excluding fixation errors or missed responses). Second row from indicates the test contrast (%), left column indicates session number. Center entries indicate number of trials.

Automated threshold setting to obtain uniform spontaneous activity levels across sessions

Although the initial stages of threshold setting and spike extraction were conducted manually (using CSC Spike Extractor software), this method did not yield closely matched levels of spiking activity during the spontaneous period, across sessions. As such, our next step was to ensure that spontaneous activity levels remained consistent across sessions.

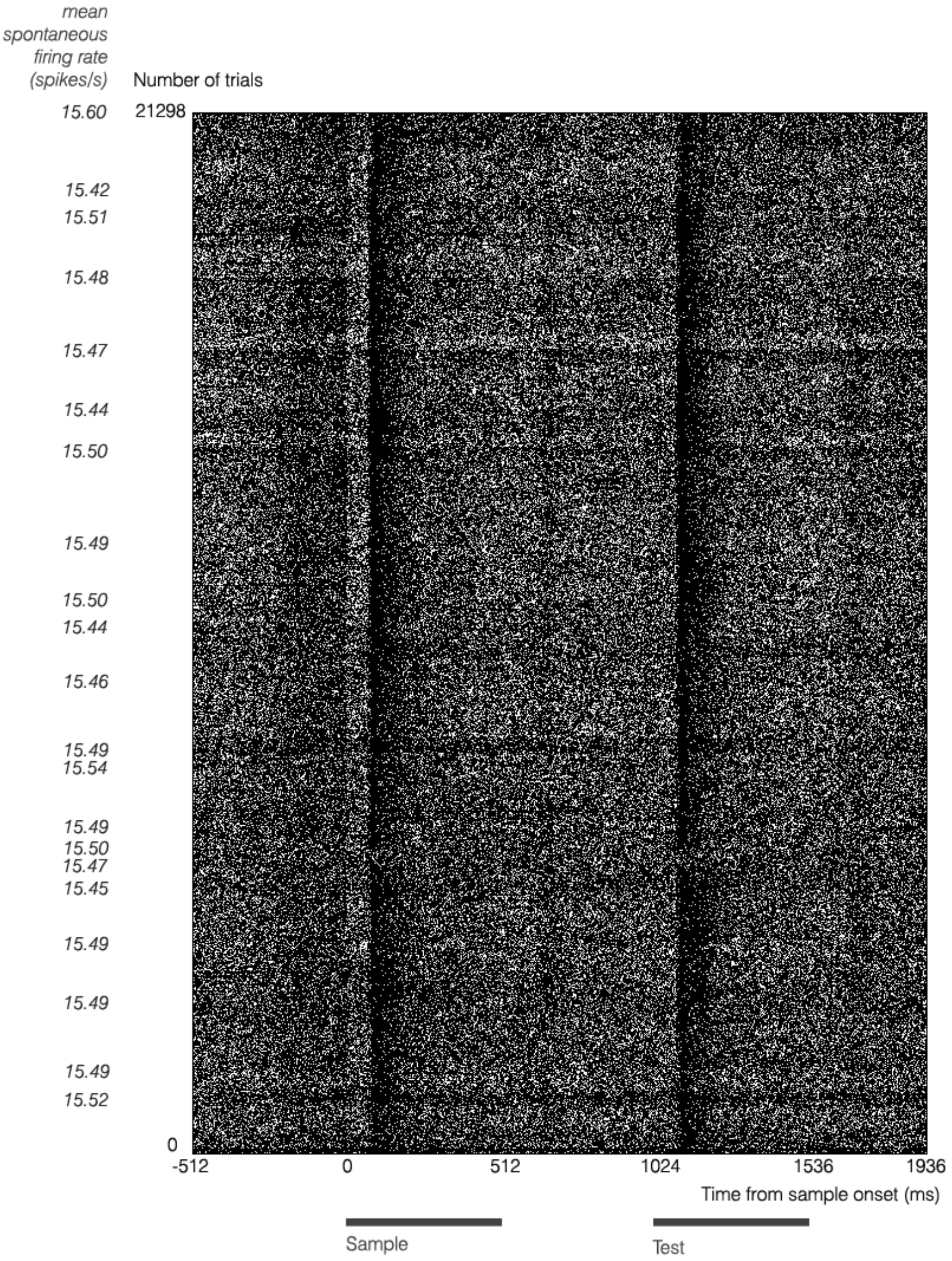

Supplementary Figure 4. ***Example Raster plot across sessions after baseline activity matching.*** Rasters plotted across multiple sessions, over a total of 21,298 trials for channel 4 in monkey 1. To the left of the plot, the mean spontaneous firing rate is displayed for each session (sp/s). The spike extraction threshold was derived using an automated staircase procedure, and the threshold for each session was selected such that the mean spontaneous rate differed by less than 1% across sessions. Levels of channel activity became uniform across sessions, and the SD in spontaneous activity levels between sessions was markedly reduced.

For each trial, one would expect that the period during which activity levels remained minimally affected by training should be that which occurred prior to sample onset (i.e. during the spontaneous period). We were aware that it was not possible to be certain that training did not affect the pre-sample spontaneous activity; however, in comparison to other periods within the trial (the stimulus-induced response and the inter-stimulus interval), the pre-sample spontaneous period appeared to be the most suitable candidate for an across-session reference. Thus, an additional step of data processing was implemented, in which the selection of thresholds for spike extraction was automated using a Matlab routine, based on levels of spontaneous activity.

First, it was necessary to select a target level of spontaneous activity, which would be used as a reference across sessions. For each channel, raster plots and PSTHs were examined by eye. A session which had ‘medium’ signal quality (i.e. with an ‘average’ SNR (see above) compared to other sessions, and with satisfactory stimulus-induced responses, was selected as the reference. The level of spontaneous activity obtained during this session was taken as the ‘target’ level across all sessions (r_t), for that particular channel. We were aware that this was an arbitrary choice; however, any choice would have been arbitrary.

Once the value of r_t, was selected, a suitable threshold had to be determined for each session such that levels of spontaneous activity (r_s), lay within 1% of the target value. Spontaneous activity levels depended on the threshold value, thus an iterative procedure was implemented in which spike extraction thresholds for the non-reference sessions were adjusted using a staircase procedure until the spontaneous firing rate of a given session deviated by no more than 1% from the target rate.

An examination of PSTHs that were generated via this procedure confirmed that the standardization of spontaneous activity levels across sessions was carried out successfully (Supplementary Figure 4). For the channel depicted in Supplementary Figure 4, the SD in firing rate across sessions prior to spontaneous activity matching was 4.57 spikes/s (mean = 13.74 spikes/s).

Supplementary Notes

Supplementary note 1: Receptive field locations and stimulus position

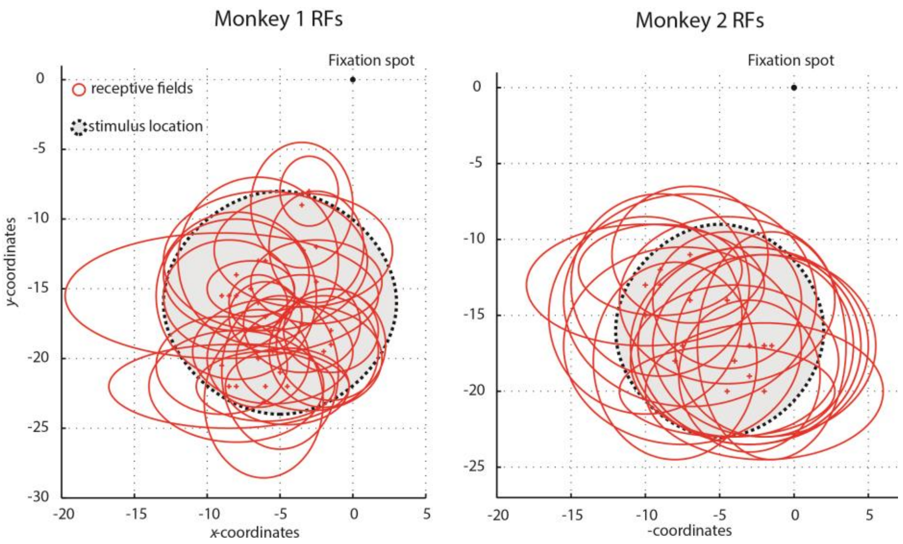

Supplementary Figure 5. ***Receptive field locations of the V4 channels in relation to stimulus location***. Dashed circle with grey area indicates stimulus location (test and sample location) in the 2 monkeys. Red circles/ellipses indicate receptive field outlines, red crosses indicate receptive field centres.

Supplementary note 2: Example of cell with inverted contrast tuning

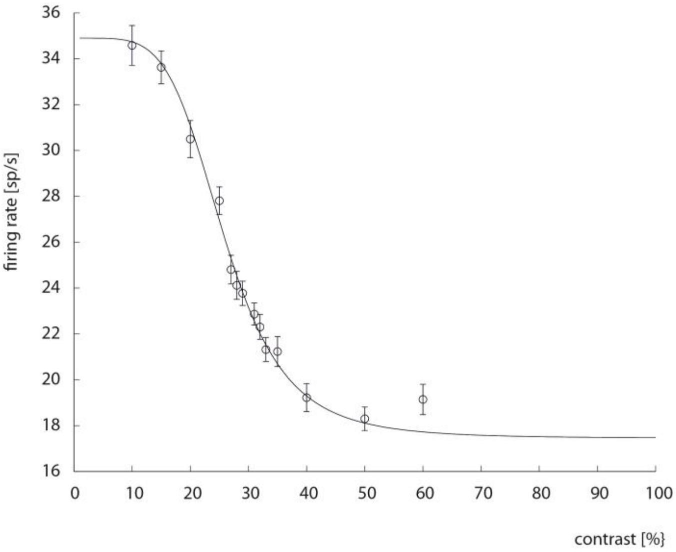

Supplementary Figure 6. ***Example of a V4 channel that had inverse contrast tuning.*** Error bars denote S.E.M. (n= 72, 70, 72, 75, 91, 101, 104, 109, 97, 76, 73, 65, 72 trials for the contrasts from 10 to 60%). Solid line indicates the best-fitted Naka-Rushton fit.

Supplementary note 3: Effects of adaptation on test responses and its possible relation to changes in PNE

A test contrast stimulus following a sample contrast stimulus in short succession is likely inducing short-term adaptation. Training might have affected this adaptation, which in turn might be the reason why changes in PNE were found.

To examine the degree of contrast adaptation in our paradigm, firing rates were compared between sample and test stimulus presentations for the population of channels. This was done for conditions where the test contrast was just above 30% and just below 30% (i.e. just above and below the sample contrast). The resulting data are shown in Supplementary Figure 7. Note that adaptation is only really defined where the test contrast is equal or higher than the sample contrast. For conditions where the test contrast is lower than the sample contrast, it would be expected that responses are lower on average even without adaptation, due to the lower stimulus contrast.

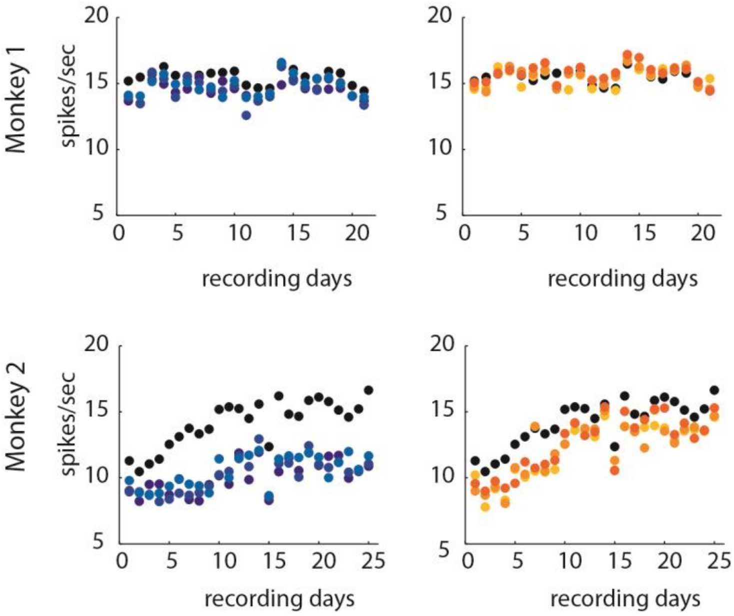

Supplementary Figure 7. ***Mean firing rates across channels against session number for sample stimuli and test stimuli with contrasts close to sample contrast.*** Sample stimulus induced firing rates are indicated by black dots. Test stimuli of lower contrast (27, 28, 29%) are indicated by blue coloured dots, whereby lower contrasts are shown by darker blue hues. Test stimuli of higher contrast (31, 32, 33%) are indicated by yellow/red coloured dots, whereby higher contrasts are shown by more yellow hues. Adaptation was more visible in monkey 2, indicated by the fact that responses to sample contrast (black dots) were larger than responses to higher test contrasts (red/yellow coloured dots).

To investigate whether the effects of adaptation changed over the course of training, an adaptation index ($AI= (sample_{activity} - test_{activity})/ (sample_{activity} + test_{activity})$) was calculated, and values of *AI* were plotted against session number (Supplementary Figure 8). Positive values of *AI* indicated stronger responses to the sample than to the test, while negative values indicated the opposite. Overall adaptation indices were lower in monkey 1 than in monkey 2 (Supplementary Figure 8).

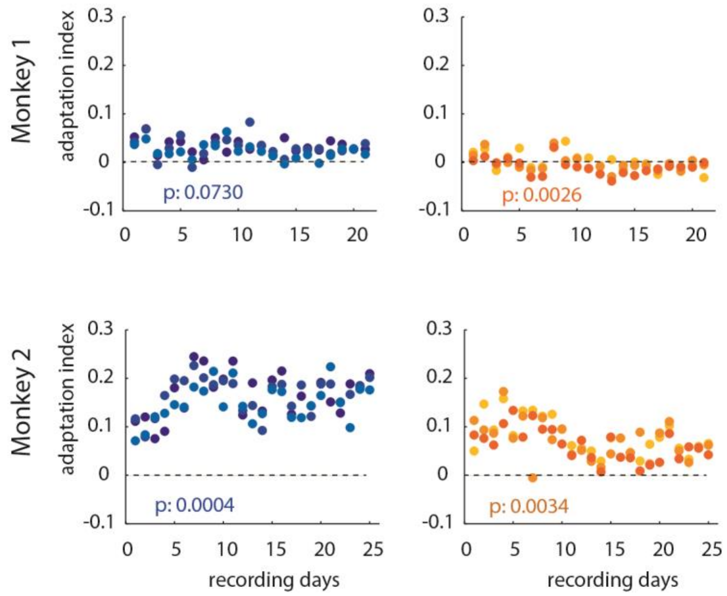

Supplementary Figure 8 ***Adaptation indices.*** Adaptation indices for stimuli of lower contrast (27, 28, 29%) are indicated by blue coloured dots, whereby lower contrasts are shown by darker blue hues. Adaptation indices for test stimuli of higher contrast (31,32,33%) are indicated by yellow/red coloured dots, whereby higher contrasts are shown by more yellow hues. Adaptation was more visible in monkey 2. P-value insets indicate whether adaptation indices were significantly different for the first 5 vs. the last 5 days (two-sided Wilcoxon sign rank test).

To determine whether adaptation indices were different for early than for late training sessions, we calculated a Wilcoxon sign rank test using the AIs from the first 5 training days and the AIs from the last 5 training days. In monkey 1 there was a trend for AIs to become less positive with training when test contrasts were lower than the sample ($p=0.073$, two-sided Wilcoxon sign rank test), and there was a significant change from positive to negative in AI when test contrast were higher than the sample contrast ($p=0.003$, two-sided Wilcoxon sign rank test). These changes are in principle compatible with an overall training induced change in adaptation. Conversely, in monkey 2 there was a significant increase in AIs when test contrasts were lower than the sample ($p<0.001$, two-sided Wilcoxon sign rank test), but there was a significant reduction in AI when test contrast were higher than the sample contrast ($p=0.003$, two-sided Wilcoxon sign rank test). Thus, there was no consistent effect of training on AIs across monkeys or the test contrasts affected, while there was a consistent increase of the slope of the contrast tuning function at the categorization boundary, and a consistent shift of the point of neuronal equality towards the categorization boundary (see main text).

Supplementary note 4: Effect of learning on the easier contrast differences

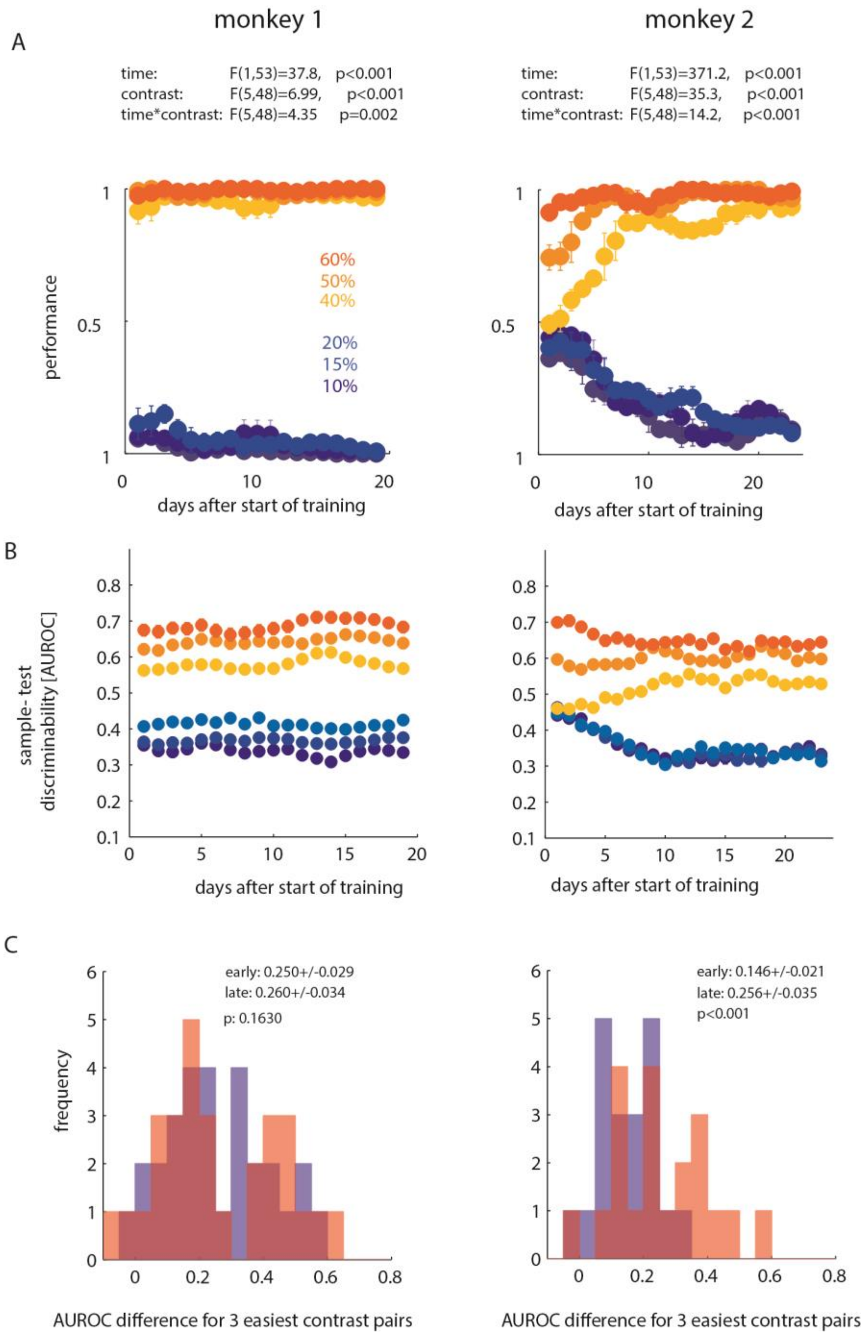

Supplementary Figure 9. ***Changes in behavioural performance and neuronal discriminability with learning for easy contrasts.*** A) Behavioural performance for the 6 easiest contrast discriminations. Performance for test contrasts lower than sample contrast (blueish colours) is plotted from 0.5 to 1 downwards. Performance for test contrasts higher than sample contrast (reddish colours) from 0.5 to 1 is plotted upwards. Test contrast colour assignment is given by coloured number insets. P-value insets are based on an ANOVA comparing the behavioural data between the first 5 and the last 5 days of training. B) Neuronal discriminability (AUROC) for sample-test contrast in the two monkeys with learning. Sample-test contrast colour assignment is given by coloured number insets in panel A. C) Distribution of discriminability difference for the 3 easiest sample test-contrast comparison pairs (e.g. 10% AUROC -60% AUROC, 15% AUROC -50% AUROC, 20% AUROC -40% AUROC) averaged across the first 5 days of learning (blue) and the last 5 days of learning (red) and across the 3 possible AUROC differences for each channel recorded. Darker red shades show overlap of the two distributions. Insets display the mean and S.E.M of the two distributions. P-values indicate whether distributions differed significantly (two-sided Wilcoxon signed rank test). Performance and discriminability for each data point is the average over 3 consecutive days, i.e. error bars denote SEM of a 3 day performance (AUROC) average (thus the number of data points are total number of recording days minus 2).

For the easiest contrast differences, behaviour changed significantly with training in both monkeys. This was particularly pronounced for monkey 2 (Supplementary Figure 9, see insets for statistics, 2 factor ANOVA). In monkey 1, no significant changes in AUROC occurred with training, whereas in monkey 2, the AUROC changed significantly (Supplementary Figure 9C).

Supplementary note 5: Control for spatial attention

Contrast tuning functions, learning and attention: Attention exerts modulatory effects on the CRF, which are represented by the response gain, contrast gain, and additive models of

attention[2-4]. One could argue that the shifts in PNE that were observed in V4 over the course of learning might not have been due specifically to improvements at the perceptual level on the contrast discrimination task, but rather to a general effect of attention. If, for example, top-down attention triggered a shift in the PNE towards the sample contrast, and this effect was strengthened as a result of training, then one might see such a shift due to the tuning of mechanisms at higher levels of the cognitive hierarchy, without the direct involvement of area V4.

To address this issue, a control task was performed with monkey 2 to investigate whether the presence of spatial attention affected contrast-dependent responses to the stimuli used during training; specifically, we wanted to determine whether it was able to induce a shift in the location of the PNE of the AUROC function.

During this stage, two sets of visual stimuli were shown on screen simultaneously- one set was located in the lower left visual field (within the V4 neuronal RFs), while the other was located in the upper visual field (outside the RFs). Stimuli in the RFs always consisted of vertically oriented sinusoidal gratings of varying contrast, whereas stimuli outside the RFs always consisted of pairs of sinusoidal gratings at 96% contrast (one vertically oriented, the other horizontally oriented). For one-half of each recording session, the animal had to attend to stimuli within the RFs and perform a contrast discrimination task, in which he discriminated between two sequentially presented stimuli of different contrasts (the same as the main task). During the other half of the session, he had to attend to stimuli outside the RFs and perform an orientation discrimination task.

As with the previous sets of AUROC analysis, cumulative AUROC values were calculated across channels. This was done separately for stimuli presented during the contrast discrimination task (when attention was directed to the RFs) and during the orientation discrimination task (when attention was diverted away from the RFs). A three-way ANOVA was performed, with the locus of spatial attention (within or outside the RFs), session number, and test contrast as factors. A significant main effect of attention was observed ($F(1,202) = 8.5$, $p = .0039$). Post-hoc tests revealed that AUROC values were somewhat smaller for low contrasts, when attention was within the RFs (corresponding to a downward shift in the range of the AUROC function). However, the presence of spatial and task-dependent attention had no systematic effect on the PNE. Hence, the shifts in PNE were not merely an attention-induced artefact.

Noise correlations, learning, and attention: To control for attention related effects on training induced changes of noise correlations, we analysed noise correlations for the attention control task in monkey 2 (6 recording sessions, attention was switched within sessions between the PL task at RF location and an orientation discrimination task in the opposite hemifield). We found that, once learning has taken place, switching spatial attention from the RFs of the neurons to a location in the opposite hemifield (and switching from a contrast discrimination to an orientation discrimination task) did not affect noise correlations in V4 (2 Factor ANOVA, factor attention: $F(5292,1)=0.9$, $p=0.3428$).

Supplementary note 6: Information gain across contrast pairs with training

To investigate how different channels encoded information across different contrast pairs, we calculated the correlation coefficient of the Fisher information between early and late stages of learning. This was done within contrast pairs (e.g. Fisher information for the 29-31% contrast pair for early and late stages respectively), as well as between contrast pairs (e.g. Fisher information for the 29-31% contrast pair for early vs e.g. Fisher information for the 20-40% contrast pair for late stages). The correlation coefficients thus obtained are shown colour coded in Supplementary Figure 10. The figure equally shows how Fisher information gain is

correlated with the amount of Fisher information present in early training periods for the different contrast pairs.

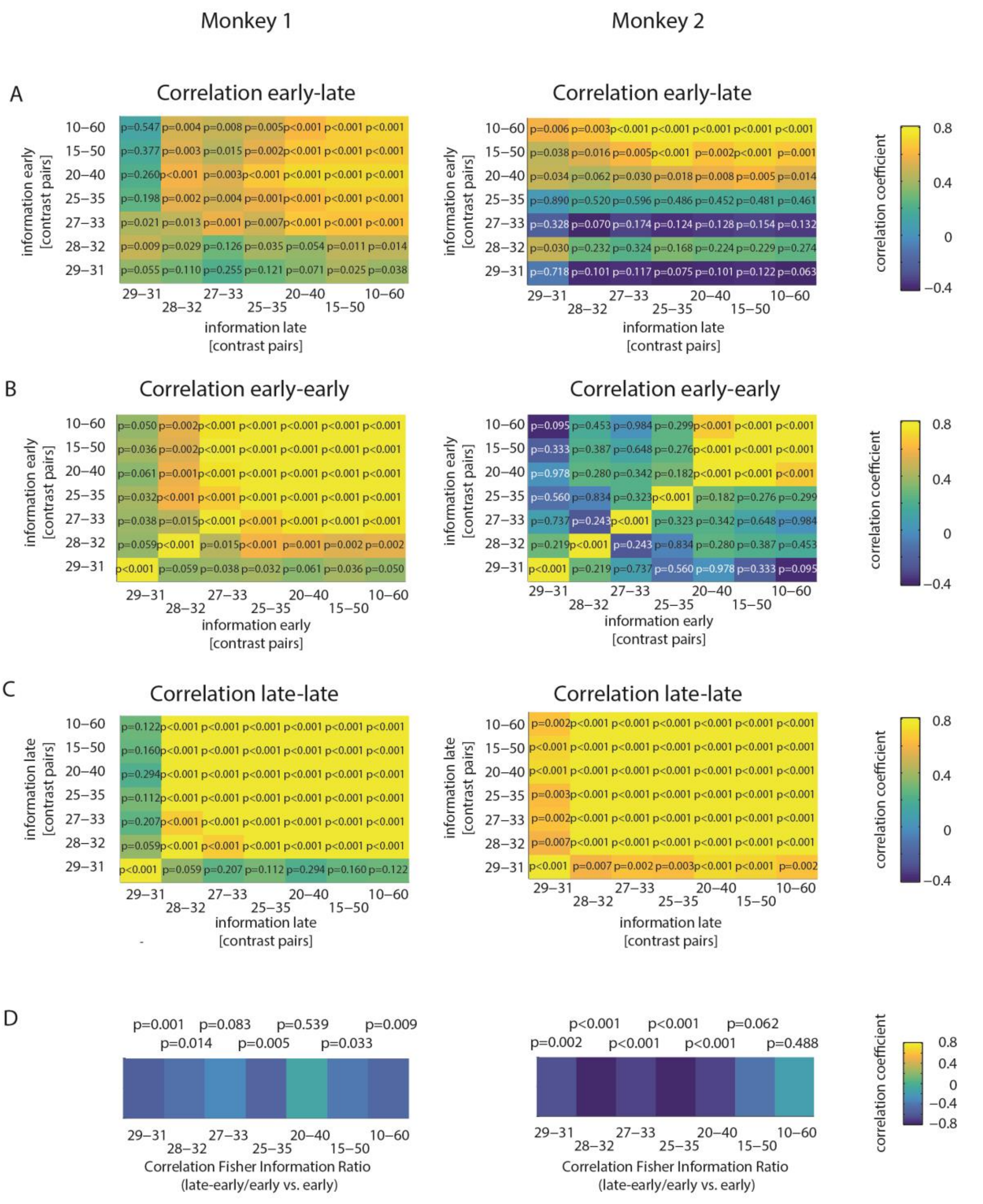

Supplementary Figure 10. ***Correlation of Fisher information across different contrast pairs for the different training periods.*** A) Correlation between early and late Fisher Information values for different contrast pairs. In both monkeys, correlation between Fisher information values for easy contrast pairs (10-60% contrast) during early training stages and Fisher information values for difficult contrast pairs (29-31%, 28-32% contrast) during late training stages were relatively large and positive. This indicates that Fisher information for difficult contrast pairs during late training periods is mostly encoded by neurons, which show differential activity for large contrast differences during early stages of training. B) Correlation of Fisher information for different contrast pairs during

early training periods. C) Correlation of Fisher information for different contrast pairs during late training periods. D) Correlation of Fisher information values during early stages of training (for different contrast pairs) with the ratio of Fisher information difference relative to early sessions (Ratio= (Fisher Information late- Fisher Information early)/ Fisher Information early). Correlations are colour coded; p-values of correlations are shown above the respective contrast pairs. Negative correlations indicate that neurons which initially coded little information about specific contrast pairs gained the most information. Correlations and p-values are calculated after averaging across recording days, so test statistics are based on n=number of recording channels (n=29 monkey 1, n=20 monkey 2).

Supplementary note 7: Training effects on working memory, decision related or motor preparation encoding in neuronal responses

In the binary decision making task, the test stimulus was always followed by a cue change and appearance of two target squares to which a left or right (relative to the stimulus) saccade had to be directed (motor response). The cue and saccade target appeared 512ms after test stimulus offset. Given the binary structure of the task, it might be that some aspects of the discriminative response (the increased information coding abilities reported in the main manuscript) were attributable to memorizing, decision making or and preparing the upcoming motor response. This would suggest that during the post stimulus period (after test offset), information about upcoming decisions would increase with learning. To investigate this, we calculated the Fisher information encoded during the post stimulus (test) period and compared it to the Fisher information encoded during the test presentation period. We found that in most (but not all) channels the information during the post-test period is close to 0. Moreover, we found no systematic learning induced increase of the Fisher information across monkeys. It suggests that in the large majority of channels the learning induced changes were related to stimulus discriminability and not related to improved working memory about upcoming decisions or motor preparation. The data for the information related to easy discriminations (where decision certainty and motor planning responses would be expected to be most clear-cut) are shown in Supplementary Figure 11.

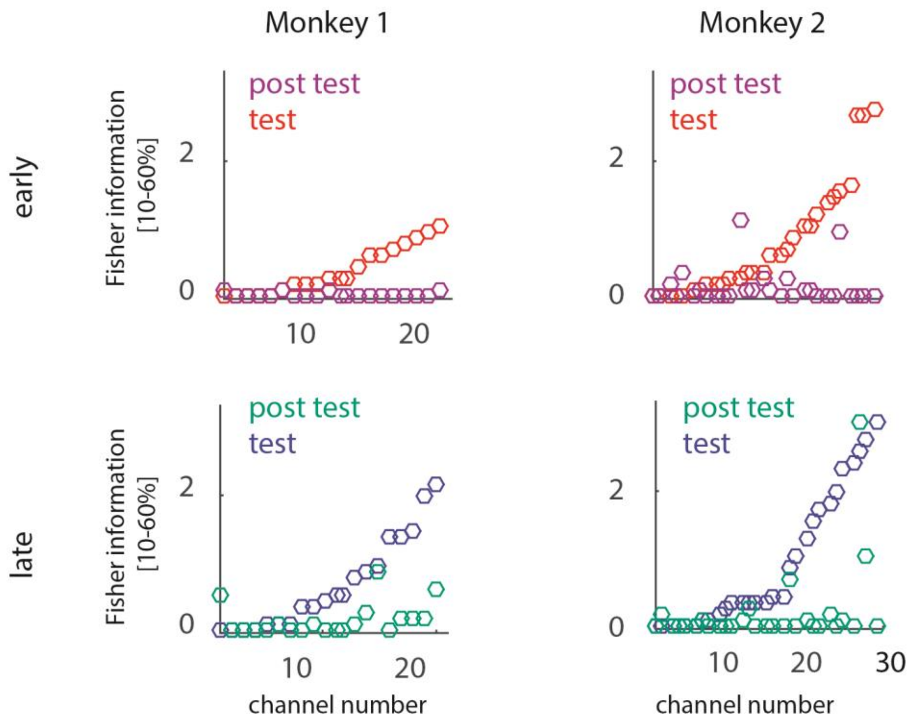

Supplementary Figure 11. ***Fisher information for the easiest contrast pair (10-60%contrast) for different channels for the test stimulus and the post-test stimulus period.*** Red and blue show Fisher information for the periods when the test stimulus was presented. Purple and turquoise show Fisher information after the test stimulus was extinguished, and before the saccade target were presented. Fisher information was sorted separately for early and late test sessions, based on test period information content. In most channels the Fisher information was close to zero after test offset.

Supplementary note 8: Untrained control conditions: Orientation and spatial frequency tuning across recording days

For most days, we recorded the orientation and spatial frequency (SF) selectivity of all recording channels that were covered by the stimulus location. Here we presented gratings of different orientation and SF at the location where we also presented the perceptual learning stimuli. In monkey 1, we presented 144 stimulus conditions for the orientation and SF mapping (12 orientations, from 0° to 165° in 15° steps; 6 SFs: 0.125, 0.25, 0.5, 1, 2 and 4 cycles per degree (cpd); 2 phases: 0.5 and 1.5). In monkey 2, 72 stimulus conditions were presented for the orientation and SF mapping (12 orientations, from 0 to 165° in 15° steps; 3 SFs: 0.125, 0.25 and 0.5 cpd; 2 phases: 0.5 and 1.5). The stimuli were presented pseudo-randomly (reverse correlation) for 141 ms, without an inter stimulus interval for at least 10 times each.

To calculate the orientation and spatial frequency selectivity for each channel on each day, the response from 50 to 150 ms after each stimulus was calculated. On each day, the preferred SF was determined as the SF at which that channel had the largest activity. For that SF, responses to different orientation were considered for computing orientation-tuning function. Responses to different orientation were scaled to have a range from 0 to 1 on each day. A wrapped Gaussian function was then fitted to these normalized responses of different orientation as follows (least square fitting):

$$Y(\theta) = \mathrm{B} + A \sum_{n=-5}^{n=5} exp\left(\frac{-(\theta - P + 180n)^2}{2\sigma^2}\right) \quad (Supplementary\ equation\ 1)$$

where Y(θ) is the predicted response for the given stimulus orientation θ, B is the baseline, A is the amplitude of the tuning curve, P is the preferred orientation and σ is the bandwidth of the tuning curve. The parameters A and B were constrained to be within ±20% of the difference between the maximum and minimum response and minimum response, respectively. P was constrained to be between 0° and 180°.

The goodness-of-fit on each day was quantified by calculating the accounted variance for as:

$$SS_{err} = \sum_{i=1}^{n} (y_i - r_i)^2 \quad (Supplementary\ equation\ 2)$$

$$SS_{tot} = \sum_{i=1}^{n} \left(r_i - mean(r)\right)^2 \quad (Supplementary\ equation\ 3)$$

$$accounted\ variance = 100 \times \left(1 - \frac{SS_{err}}{SS_{tot}}\right) \quad (Supplementary\ equation\ 4)$$

where r_i is the mean response to the ith orientation, y_i is the predicted response to the ith orientation and SS_{tot} is the total sum of squares and SS_{err} is the residual sum of squares and n is the total number of orientation (i.e. 12).

We used two different inclusion criteria to determine whether preferred orientation changed across the population with training. (1) We included all channels and days, irrespective of the amount of variance the orientation tuning fit accounted for, and (2) for each channel, we included only days where the orientation tuning fit accounted for ≥ 50% of the variance. For

each channel, we subtracted the orientation of the presented stimuli (90°) from the preferred orientations to obtain ΔP to determine the changes in the preferred orientation with training relative to the orientation of the presented stimuli:

$$\Delta P_i = |\,P_i - 90| \quad (Supplementary\ equation\ 5)$$

Where i is the day and P_i is the preferred orientation on i^{th} day.

We calculated the correlation (Spearman rank correlation) between preferred orientation and recording day and between the values of ΔP and the recording days. We did this in 2 different ways. Firstly, we averaged the ΔP values across channels for each recording days, and used these average values for the calculation of the correlation. Secondly, we calculated for each channel the correlation between preferred orientation changes and recording days, to determine in how many channels such a correlation existed. If preferred orientation systematically shifted away or towards the orientation of the contrast discrimination stimuli (which had a fixed orientation of 90°), a significant correlation would be expected (as seen for e.g. the PNE of the neurometric fits to the contrast tuning curves).

We found no correlation between changes of orientation tuning with training in either animal. Using all data (irrespective of the orientation tuning fit quality (variance accounted for)), we obtained the following correlations between the preferred orientation and recording day: monkey1: r = -0.01, p = 0.67; monkey 2: r = 0.01, p = 0.81). Calculating the correlation between |preferred orientation – 90| and recording day we obtained: Monkey 1: r = 0.09, p = 0.03; monkey 2: r = -0.01, p = 0.74.

Including instead only preferred orientations where for a given channel the orientation tuning fit accounted >50% of the variance we obtained for the correlation between preferred orientation and day: Monkey 1: r = -0.03, p = 0.60); monkey 2: r = 0.01, p = 0.87. Calculating the correlation between |preferred orientation – 90| and recording day we obtained: Monkey1: r = 0.02, p = 0.63; monkey1: r = -0.05, p = 0.41.

Correlations of preferred orientation changes values with recording days for individual channels equally did not result in consistent changes. For this analysis, we included days when the orientation tuning fitted accounted > 50% of the variance. In Monkey 1 four channels showed a significant correlation between preferred orientation and recording day: in one channel the preferred orientation changed from ~90° to ~70° (r = -0.56, p < 0.01); in the second one it changed from ~100° to ~160° (r = 0.67, p < 0.005); in the third one it changed from ~50° to ~90° (r = 0.66, p < 0.03) and in the fourth one it changed from 150° to 155° (r =0.49, p < 0.03). In Monkey 2 only one channel showed significant changes in the preferred orientation as a function of recording day (from ~30° to ~5°, r = -0.75, p < 0.001). Thus, in the majority of channels (~90%) there was no systematic shift with training, and in the few where this did occur, there was no systematic relationship between shift direction and trained orientation.

Finally we analysed whether tuning width changed with training. We included tuning bandwidth where for a given channel the orientation tuning fit accounted >50% of the variance. We then correlated average tuning width against recording/training days. We found no correlation between tuning bandwidth and day for Monkey 1: r = -0.01, p = 0.76; and a small, but significant change in tuning width in monkey 2: r = -0.13, p = 0.04, whereby tuning became slightly more narrow with training. We also analysed whether tuning width changed for individual channels. Neither in Monkey 1, nor in Monkey 2 did any channel significantly change tuning bandwidth over the course of the recording/training period.

Supplementary note 9: Single unit data

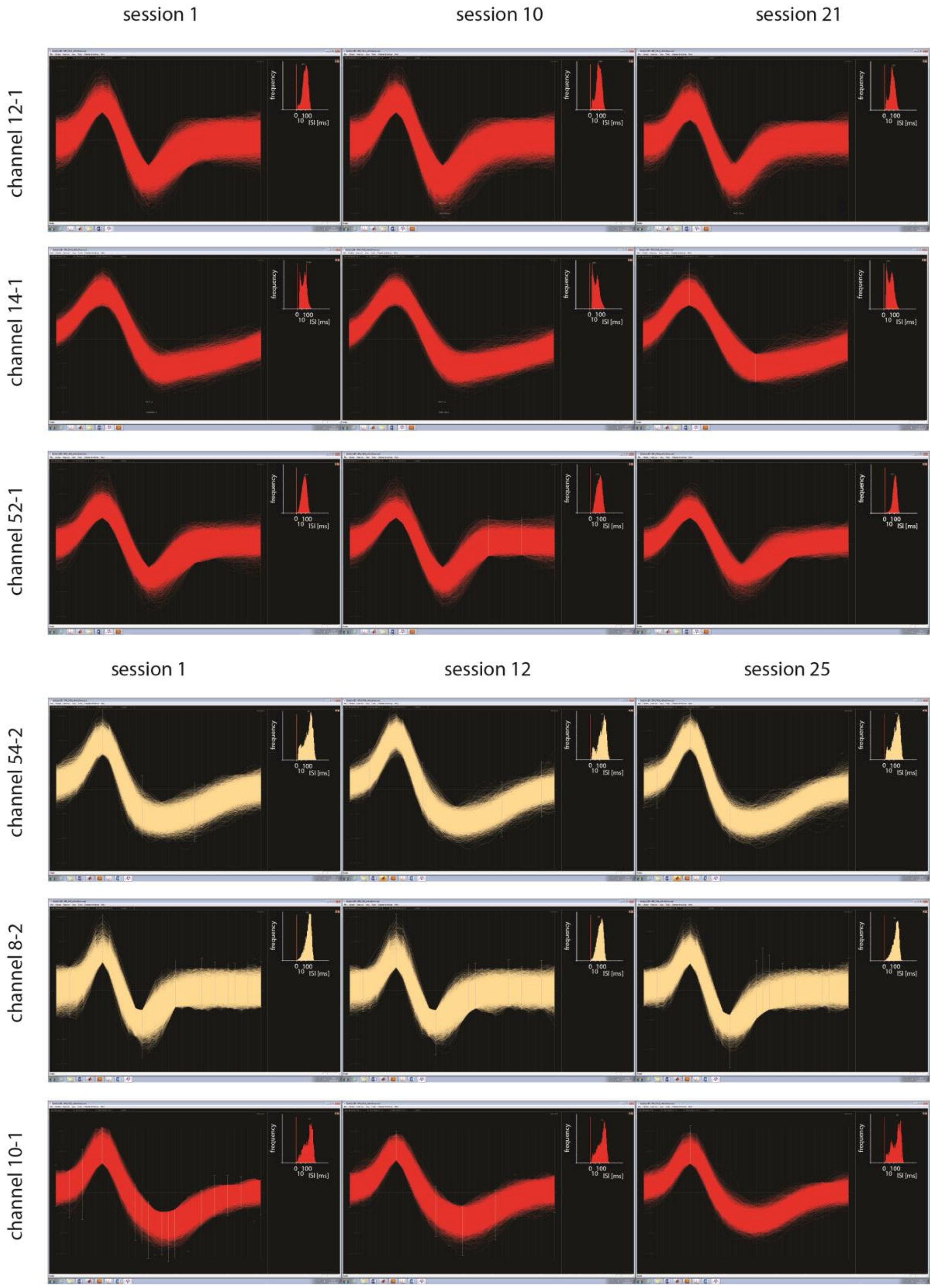

Supplementary Figure 12. *Example waveforms from the spike sorting, where single unit stability was good across all recording sessions.* The top 3 examples are from monkey 1, bottom 3 examples are from monkey 2. Waveforms and Interspike interval histograms (ISI, insets to the right) are shown for each cell and session. Color coding (red vs. yellow) is based on whether the cells was arbitrarily assigned the label '1' or '2' by Spikesort3D

(Neuralynx), but has no relevance otherwise. ISIs show frequency of ISI during the recording session. The red vertical bar is time 0, the next x-axis tic indicates an ISI of 10ms, followed by 100ms (i.e. logarithmic scale).

Spike sorting: We first manually spike sorted each channel (n=29 for monkey 1, n=20 for monkey 2) for each session (n=21 recording days/session for monkey 1 and n=25 recording days/session for monkey 2; manual spike sorting; SpikeSort3D 2.5.4, Neuralynx). Here we used the spike sorting template obtained from manual spike sorting from a recording session that was half way through the training period as an initial guidance for sorting of all the other session. Session/recording day #10 was used for monkey 1 (out of 21 sessions total), and session/recording day #12 was used for monkey 2 (out of 25 sessions total). We then used waveform similarity, interspike interval similarity and waveform feature cluster information as a guidance for the manual spike sorting of the remaining sessions. Quality of spike sorting was assessed by visual inspection of waveforms and of the interspike interval histograms (ISI). We aimed to generate ISI histograms where the bin representing <=1ms delays was empty. Where that was not possible, we aimed to minimize (A) the number of ISI with 1-4 ms, and (B) maximize the number ISI bins ranging from 5-200 ms. This spike sorting resulted in a total of 34 units (from 29 recording channels) for monkey 1 and 27 units (from 20 recording channels) for monkey 2, i.e. from some of the channels more than 1 unit was sorted (this was fixed across all recording days, i.e. where it was possible to obtain 2 units from a channel on day #10 (#12 respectively in monkey 2), 2 units were sorted from that channel on all recording days.

This spike sorting resulted in isolating some single units, and some of these appeared to be consistently present across all recording sessions. Specifically, from the 34 units sorted on day #10, we obtained 9 single units for monkey 1, and from the 27 units sorted on day #12, we obtained 5 single units for monkey 2, that we considered to be stable across recording session. Supplementary Figure 12 gives 6 examples of single units that could be extracted across all recording sessions in the two monkeys.

Contrast tuning functions and neurometric functions for single units

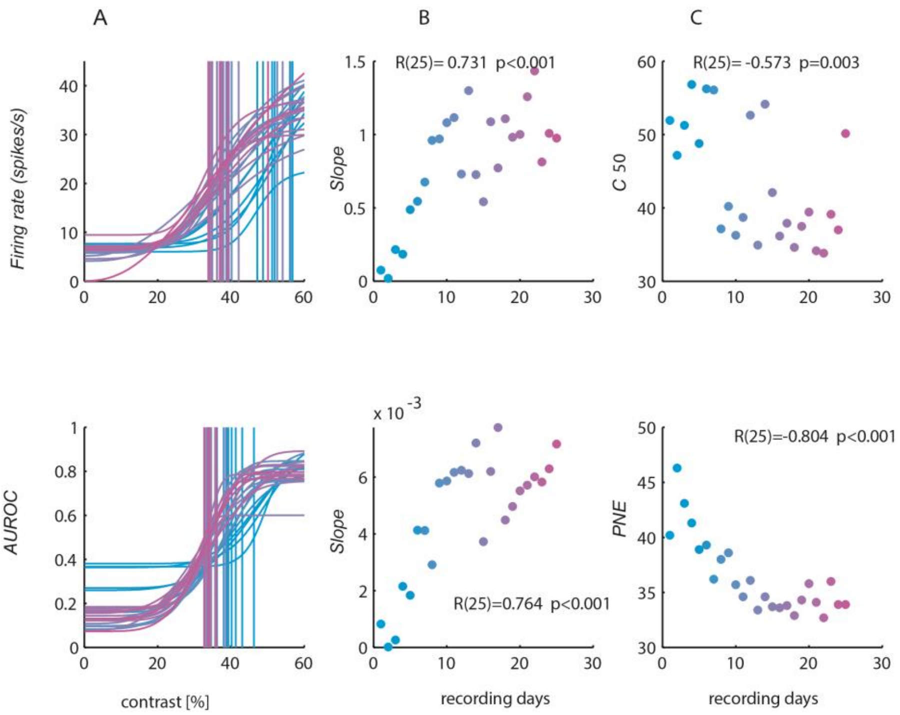

Supplementary Figure 13. ***Example of learning effects on a single unit's contrast response and neurometric functions.*** A) Contrast response functions and neurometric function as a function of learning (color coded blue to purple as learning progressed over recording days). Vertical lines show location of C_{50} for each recording day. B) Slope of the contrast response function and neurometric function at 30% (the sample contrast). C) Change of the C_{50} and the PNE with learning.

Analysis of single units yielded qualitatively and quantitatively similar data to the approach where baseline activity was equated across sessions. Example data from a single unit are shown in Supplementary Figure 13. For this example cell, the CRF and neurometric function

became steeper (at 30% contrast) over the course of training (Supplementary Figure 13B). Moreover, the C_{50} and the PNE shifted towards the value of 30% with training (Supplementary Figure 13C). The example shown reflects the pattern seen across the population. Averaging parameters from single units for a given recording day, and determining their changes across recording days yielded results qualitatively identical to those presented for the multi-unit data in the main manuscript (Supplementary Figure 14). For the population of single units, the slope at 30% increased significantly with training in both animals irrespective of whether assessed for neurometric or Naka Rushton functions. Additionally the PNE moved significantly closer to 30% in both animals.

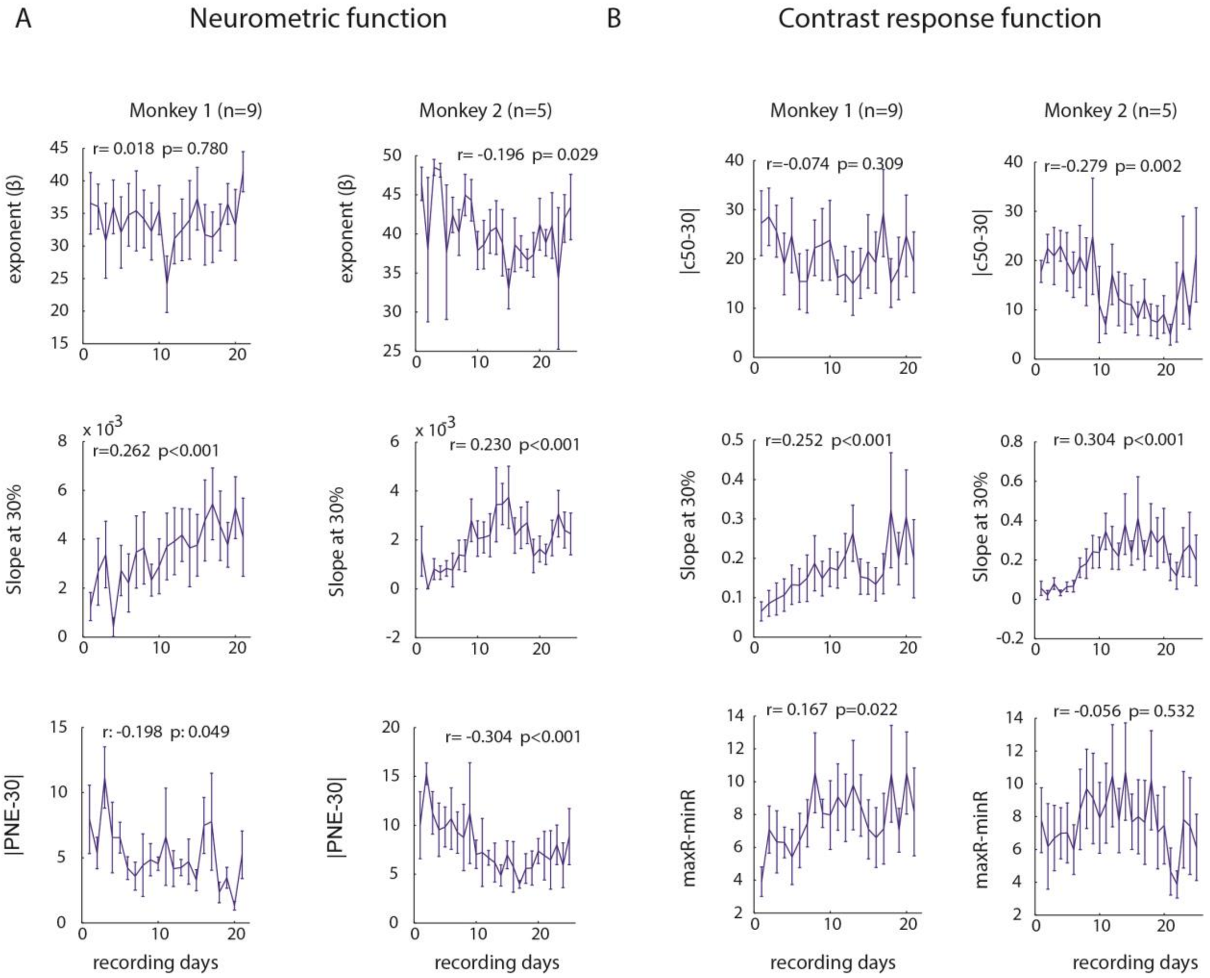

Supplementary Figure 14. ***Parameters of neurometric and Naka-Rushton functions as learning progressed for averaged single unit data.*** Learning induced changes in selected parameters of the neurometric function (fitted with a Weibull function) and of the contrast response function (fitted with a Naka-Rushton function). A) Changes in location where the neurometric function reaches 63% of its range, the slope at 30% contrast, and point of neuronal equality (|PNE-30|) of the neurometric function. B) Changes in $|C_{50}\text{-}30|$of the Naka Rushton function, its slope at 30%, and rate ranges (difference between minimum and maximum measured activity). Insets show the Spearman rank correlation coefficients (r) and the p-value (p) of the parameter of interest (dependent variable) vs. recording days (independent variable). Error bars denote S.E.M. (n=9 and n=5 per data point for monkey 1 and 2 respectively).

Changes in test-sample neuronal discriminability with learning

Significant increases in sample-test discriminability occur in both animals with training when analysis was based on stable single unit recordings. These increases occurred for difficult test contrasts (Supplementary Figure 15; 27-33%, Wilcoxon signed rank test; p=0.046 [monkey 1], p<0.001 [monkey 2]), and for easier test contrasts (data not shown; 5-30% test contrast difference to the sample, Wilcoxon signed rank test; p=0.046 [monkey 1], p=0.037 [monkey 2]).

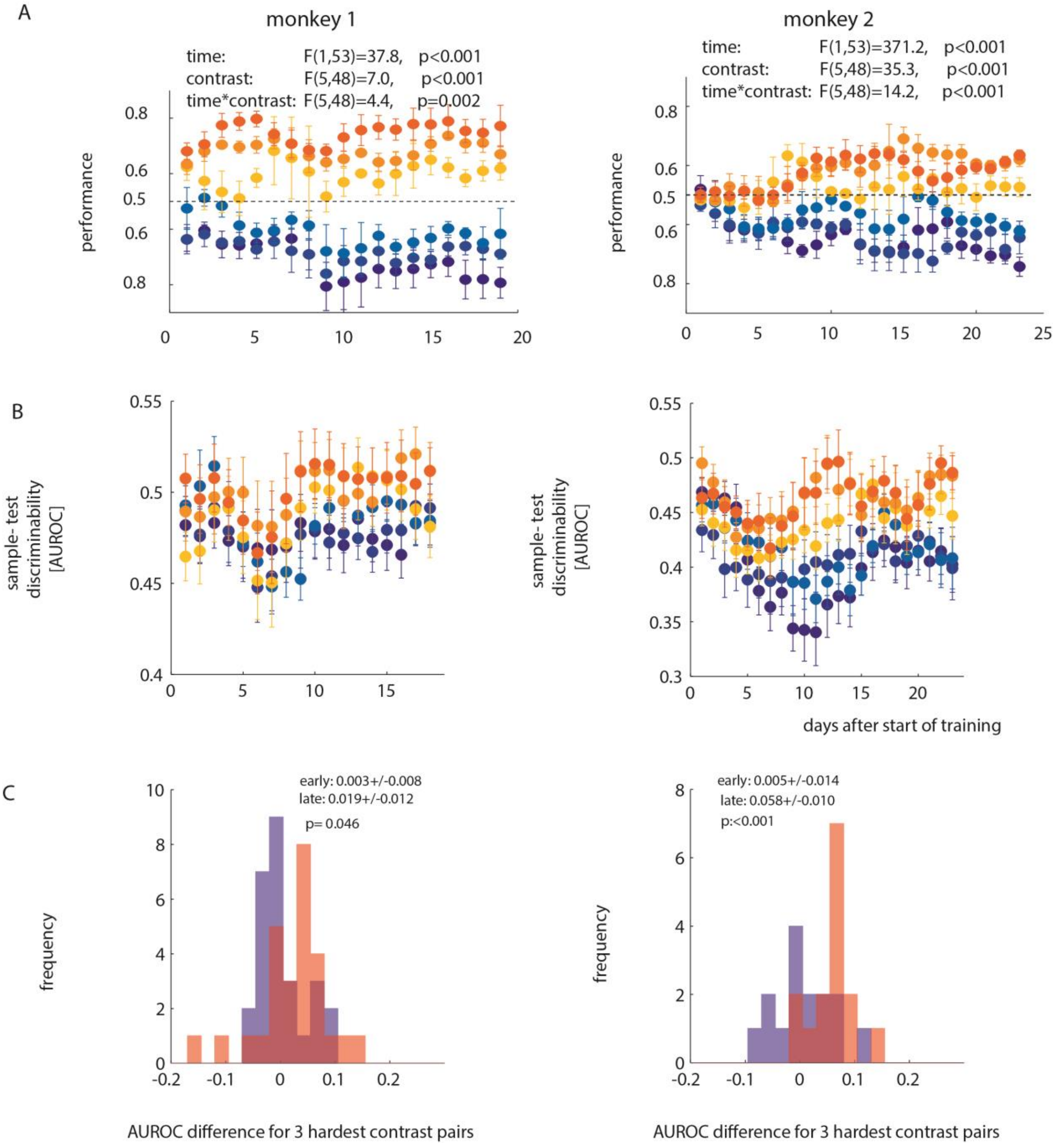

Supplementary Figure 15. ***Changes in behavioural performance and neuronal discriminability assessed for single unit recordings (n=9 for monkey 1 and n=5 for monkey 2) with learning.*** A) Behavioural performance for the 6 most difficult contrast discriminations. Performance for test contrasts lower than sample contrast (blueish colours) is plotted from 0.5 to 1 downwards. Performance for test contrasts higher than sample contrast (reddish colours) from 0.5 to 1 is plotted upwards. Test contrast colour assignment is given by coloured number insets. B) Neuronal discriminability (AUROC) for sample-test contrast in the two monkeys with learning. Sample-test contrast

colour assignment is given by coloured number insets in panel A. C) Distribution of discriminability difference for the 3 most difficult sample test-contrast comparison pairs (e.g. 31% AUROC -29% AUROC, 32% AUROC -28% AUROC, 33% AUROC -27% AUROC) for the first 5 days of learning (blue) and for the last 5 days of learning (red) across all channels recorded. Darker red shades show overlap of the two distributions. Insets display the mean and S.E.M of the two distributions. P-values indicate whether distributions differed significantly. Performance and discriminability for each data point is the average over 3 consecutive days, i.e. error bars in A and B denote SEM of a 3 day performance (AUROC) average (thus the number of data points are the total number of recording days minus 2).

Choice probability analysis

For monkey 1 we found a significant difference between CP values for late vs. early days (F(1,96)=5.28, p=0.02, 2 factor ANOVA), no effect of contrast ((F(5,96)=1.4, p=0.231, 2 factor ANOVA), and no interaction (F(5, 96)=1.03, p=0.407, 2 factor ANOVA). Post-hoc testing revealed that the effect was present in some, but not all of the comparisons (Supplementary Figure 16), but this is likely due to the small sample size given the single unit analysis (n=9 neurons). In monkey 2, we equally found a significant difference between CP values for late vs. early days (F(1,48)=4.6, p=0.037, 2 factor ANOVA), no effect of contrast (F(5,48)=1.27, p=0.291, 2 factor ANOVA), and no interaction (F(5,48)=0.30, p=0.908, 2 factor ANOVA). Post-hoc testing revealed that the effect was present in some but not all of the comparisons, also likely due to the small sample size given the single unit analysis (n=5 neurons).

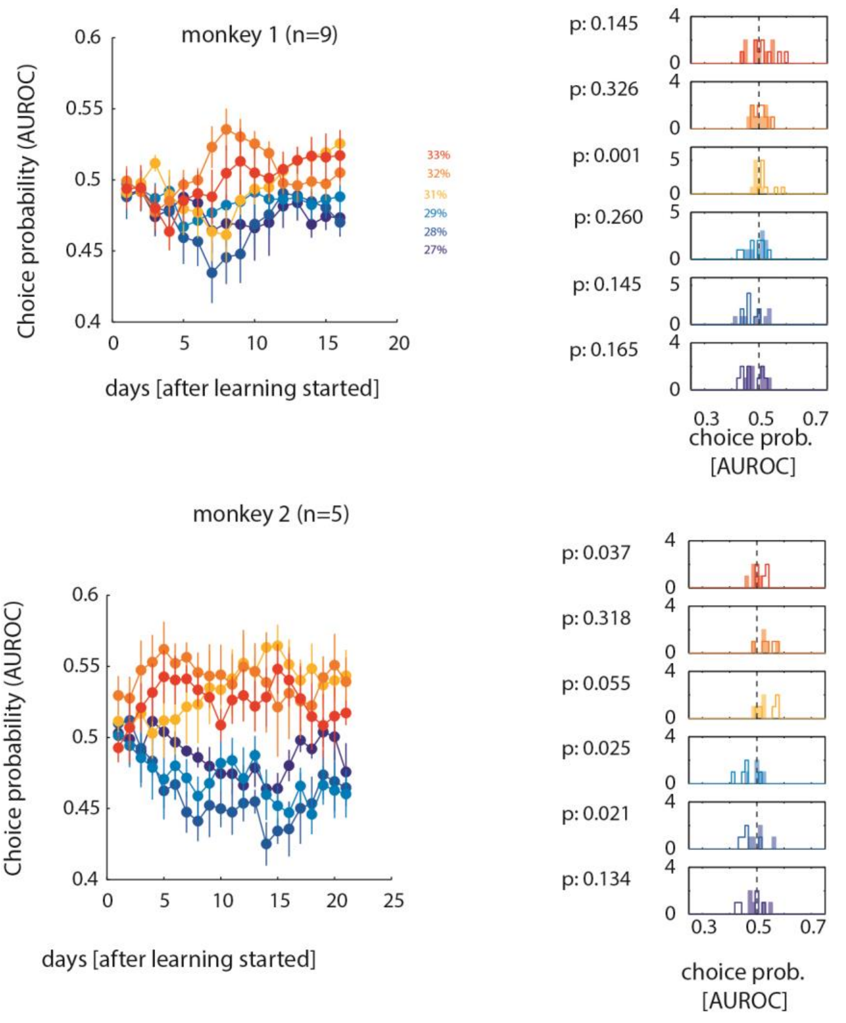

Supplementary Figure 16. *Choice probability in single units as a function of learning for both monkeys.* Choice probability for test activity levels (separately for the 3 hardest contrast levels below and above sample contrast, respectively). Histograms in the right column show distributions of choice probabilities for the first 5 days of learning (filled histograms) and the last 5 days of learning (outlined histograms). P-values for distribution differences are indicated next to the individual histogram plots. Data are averaged over 5 consecutive days, i.e. number of data points = recording days minus 4. Error bars denote S.E.M. (n=9 and n=5 per data point for monkey 1 and 2 respectively).

Effect of learning on information coding in different channels

Fisher information across cells before and after learning: In both monkeys, the ability to encode contrast differences varied substantially between cells. We ranked the cells based on the amount of information encoded during the first 5 days of training, and then performed a separate ranking based on the last 5 days of training. Supplementary Figure 17 shows that the information content varied substantially between channels. The differences in information content with training were more pronounced in monkey 2 for difficult contrasts (small test-test contrast differences), but they were more pronounced in monkey 1 for easy contrasts (large test-test contrast differences).

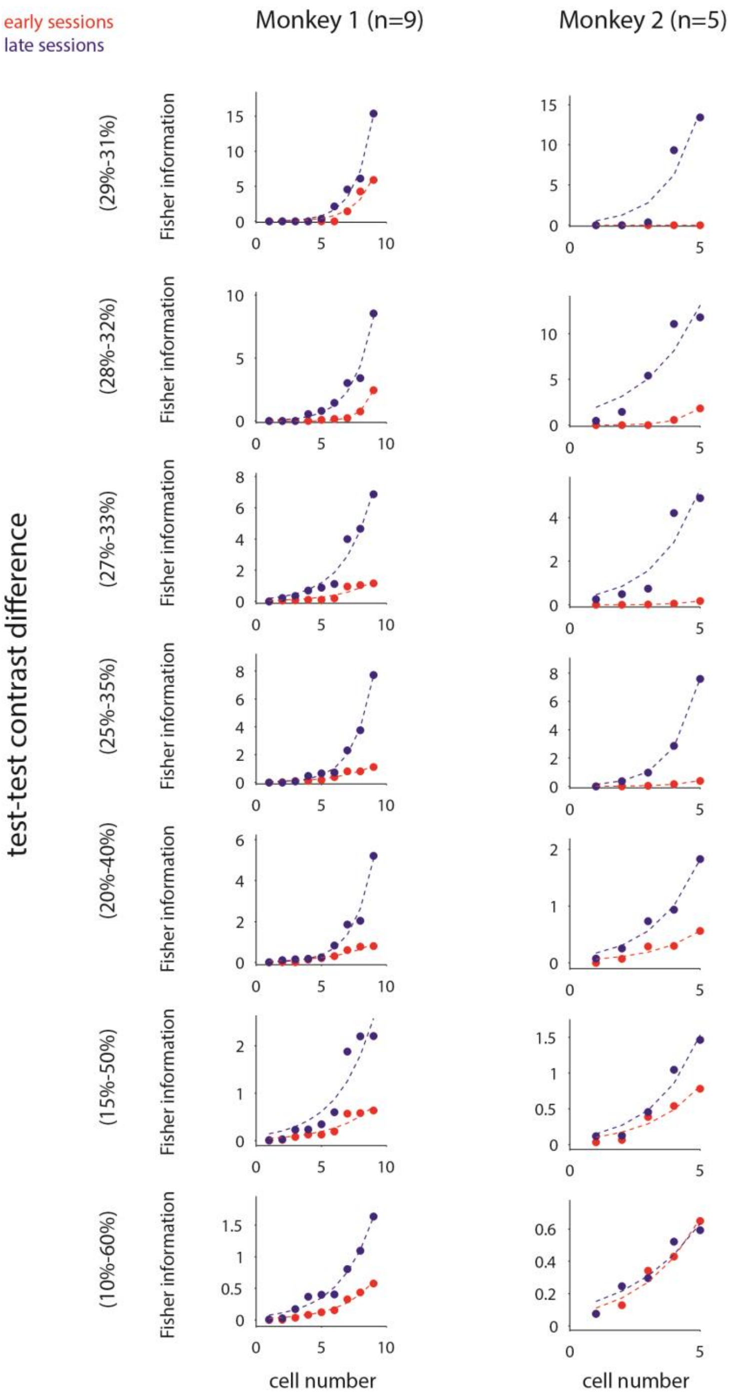

Supplementary Figure 17. ***Distribution of information content across single units.*** Rank ordered information content in single cells for different contrast pairs during early (red) and late (blue) periods of learning. In both monkeys, a sizeable number of cells encoded relatively little information during early or late training days. Differences between training days are most obvious for cells with comparatively high information content. (Note that the Information bias correction could result in negative Fisher information values, for cases where uncorrected information values were very small. In that case Fisher information was set to 0.000001, which occurred e.g. for all cells from monkey 1 for the contrast difference of 29-31% during early training periods, but it also occurred for other cell/contrast pair data points).

Information gain as a function of learning in single units: The analysis of how information was correlated between early and late sessions for single units shows the same trends as the analysis performed on the baseline matched multi-unit data. The results are shown in Supplementary Figure 18**.**

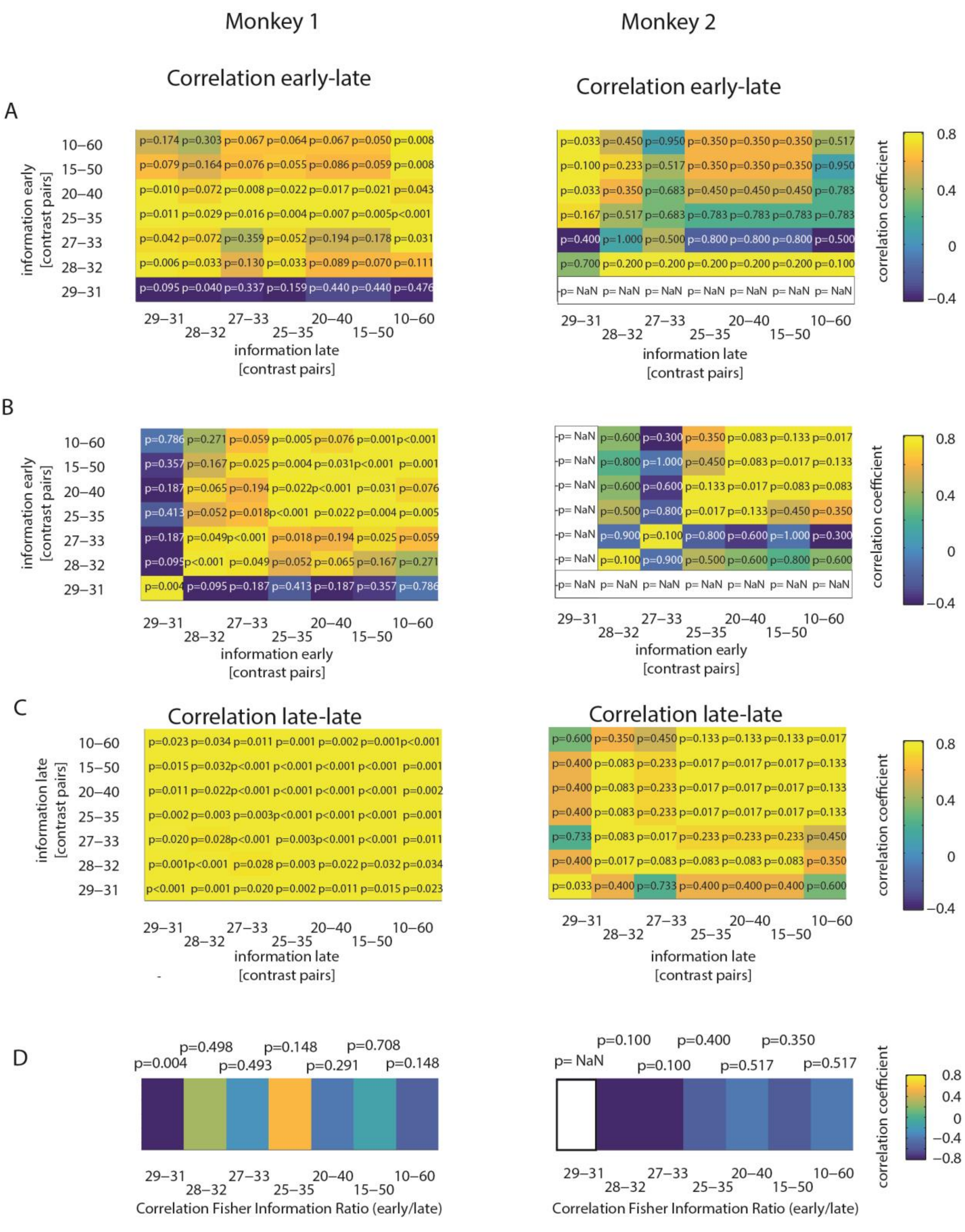

Supplementary Figure 18. ***Correlation of Fisher information across different contrast pairs for the different training periods in single units.*** A) Correlation early vs. late Fisher Information values for different contrast pairs. In both monkeys, correlation between Fisher information values for easy contrast pairs (10-60% contrast) during early training stages and Fisher information values for difficult contrast pairs (29-31%, 28-32% contrast) during late

training stages were relatively large and positive. This indicates that Fisher information for difficult contrast pairs is mostly encoded by neurons, which show differential activity for large contrast differences during early stages of training. B) Correlation of Fisher information for different contrast pairs during early training periods. C) Correlation of Fisher information for different contrast pairs during late training periods. D) D) Correlation of Fisher information values during early stages of training (for different contrast pairs) with the ratio of Fisher information difference relative to early sessions (Ratio= (Fisher Information late- Fisher Information early)/ Fisher Information early). Correlations are colour coded; p-values of correlations are shown above the respective contrast pairs. Correlations are colour coded; p-values of correlations are shown in the colour-coded sections or above the respective contrast pairs. Where p-values of correlations are shown as NaNs (not a number), the information across all comparisons of one of the sets was identical, due to the bias correction of the Fisher information analysis. The latter could result in negative Fisher information values, which was adjusted to a value of 0.00001. Correlations and p-values are calculated after averaging across recording days, so test statistics are based on n=number of single units (n=9 monkey 1, n=5 monkey 2).

Changes of noise correlation with learning in single unit neuronal populations.

Across the small sample of single units, noise correlations were not changed by training in either monkey. (Supplementary Figure 19; 2 Factor RM ANOVA, factor learning period: monkey 1: F(5012,1)=2.48, p=0.1156, monkey 2: F(1327,1)=0.36, p=5464). There was an effect of contrast in monkey 1, but not in monkey 2 (2 Factor RM ANOVA, factor contrast: monkey 1: F(5012,13)=1.85, p=0.031, monkey 2: F(1327,13)=1.22, p=0.259). There was no significant interaction between contrast and the period of training (2 Factor RM ANOVA, factor learning period*contrast F(5012,13)=0.69, p=0.779, monkey 2: F(1327,13)=1.47 p=0.119).

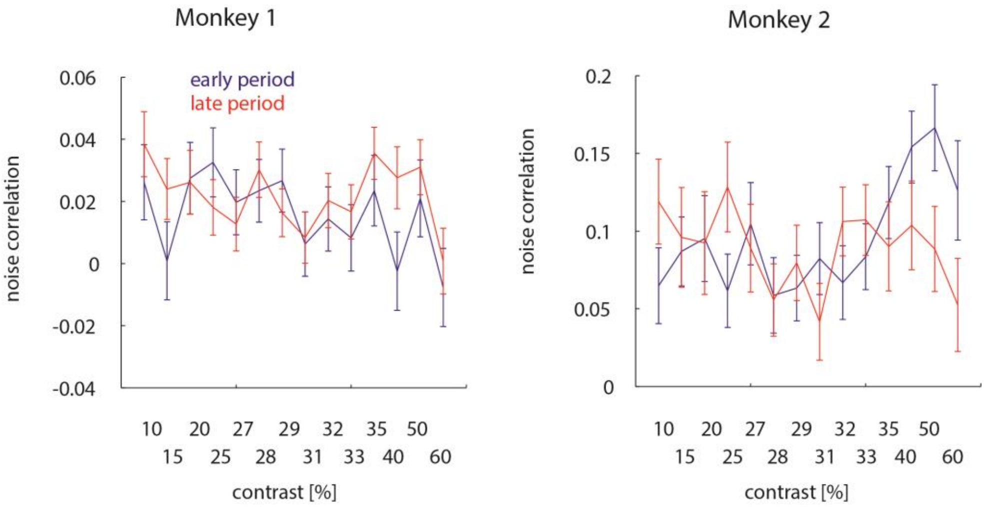

Supplementary Figure 19. ***Average noise correlations for single units as a function of training period and test contrast for the two monkeys.*** Error bars denote S.E.M. (n=9 and n=5 per data point for monkey 1 and 2 respectively).

We did not calculate the change in the slope of signal vs. noise correlations for the single units due to the small data set (n=9 and 5 respectively), which prohibited the split into the two classes cells that coded for top 33% vs. bottom 33% of information.

Changes of information content with learning in neuronal populations

We calculated the Fisher information about test contrast encoded in population activity as a function of population size for our single units. The results are plotted in Supplementary Figure 20. It shows that the information content in the population was higher during late training days than during early training days in both monkeys. Overall, the results of the stable *single unit inclusion matching* are very similar to those obtained with baseline activity matching. Differences seem to occur regarding the effect of shuffling for monkey 1. Specifically, shuffling

reduces the amount of information in this animal when ~3-4 cells are added to the pool. This is likely because in monkey 1 noise correlations are close to 0, and thus shuffling cannot destroy absent noise correlations. However, a direct comparison of how comparable noise correlations affect information in different samples (multi-units vs. single units in this case) is not straightforward because the same amount of correlations may have a substantially different impact depending on the overall magnitude of the information. We commented on this effect of the impact of noise correlations in relation to the magnitude of information when discussing Figure 7B in the main manuscript. Indeed, the magnitude of information levels is much higher, even an order of magnitude, for channels than for single units, which means that noise correlations are expected to have a bigger impact. Moreover, and as expected by theoretical considerations suggesting that the impact of noise correlations tends to grow with population size e.g. [5, 6], the effect of shuffling in MUA data became most pronounced for larger pool sizes (>10 channels). We were unable to explore this larger population size for the single unit data, given the limited sample size. This difference in the magnitude of the information, and in the number of cells accumulated contribute to explain why the effect of shuffling is much smaller for both monkeys for single units. We do not have a clear explanation of why information is decreased with shuffling for some contrast pairs of Monkey 1, although this decrease is much smaller in comparison to the increase observed at the channel level, and it is difficult to assess its significance. We would need more single units to explore if the impact of noise correlations is similar for single units and for channels when comparable amounts of information are obtained. Nonetheless, the main conclusion drawn from the shuffling analysis, namely the fact that eliminating noise correlations by shuffling is not sufficient to fully explain by itself the increase in information with learning not only is confirmed by the single unit analysis but even more it holds for also for easier contrasts pairs of Monkey 1, while for channels this conclusion was drawn only from the difficult contrast pairs.

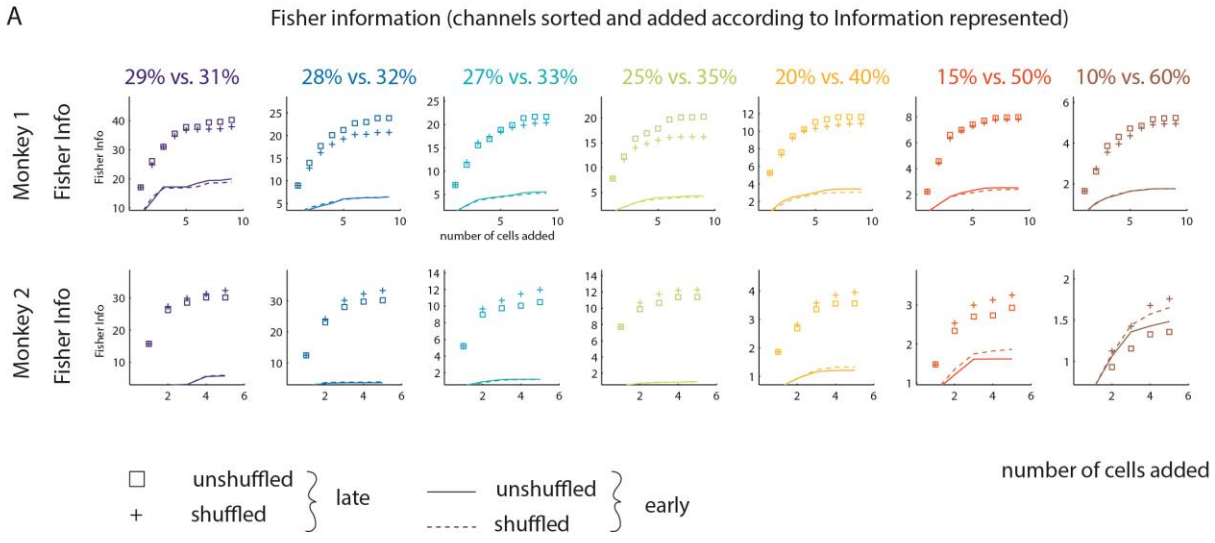

Supplementary Figure 20. ***Information provided by single units to populations of different sizes before and after learning.*** A) Fisher information in a population of consecutively added channels for early (solid and dashed lines in each subplot) and late recording days (+ and squares in each subplot). Colour coding shows the Fisher information for the different test contrast pairs investigated/decoded. 'Unshuffled' indicates that the channel identity and the correlation structure was retained during calculation of Fisher information (squares and solid lines). 'Shuffled' indicates that trials between channels were shuffled to remove the rate correlations, while retaining channel identity (+ and dashed lines). Abscissa: number of channels that were added to the population. Ordinate: Fisher information present. Channels were added based on their rank order single channel information content, starting with the channel encoding the most information.

Supplementary note 10: *Modeling learning induced information changes for single unit vs. multi unit activity*

To further examine the degree to which our conclusions regarding the changes in information with learning may be affected by the use of multi-unit activity, we generated simulated population responses according to the main properties of the estimated contrast response functions (CRFs). We then simulated the effect of the aggregation of single-unit activity (SUA) into the multi-unit activity (MUA) signal, as well as the effect of adapting the threshold for spike detection to maintain a constant baseline activity level across sessions (baseline activity matching).

To simulate the population responses we considered that in each channel a number N of neurons contributed to the MUA signal. To generate the population responses of all neurons across channels we modeled them using the estimated CRFs and noise correlations from the recorded MUA signals. Our conclusions on the Fisher information analysis for the simulated data were robust to the exact properties of the CRFs and noise correlations preserved in the simulations. We now describe the setup used for the results shown in Supplementary Figure 21 and Supplementary Figure 22 and we will later discuss how robust these results are to relaxations of the properties preserved.

We described above the extraction methods to obtain our MUA, where we performed the baseline activity matching between recording sessions. This resulted in baseline activity of about ~9-15 sp/sec for the individual channels. The average spontaneous activity in V4 for single units in a memory guided search match to sample task (before sample onset) is 3.9 sp/sec [7]. We assume that our task would generate similar single cell baseline activity. Based on this, we assume that an average ~3 cells contributed to our MUA activity. For this reasons we show all the modelling analysis for cell sizes of n<=3. However, we have also performed the simulations to up to 6 units, using identical methods, and find no qualitative differences to the modelling where <=3 cells were used.

For each day and each channel, the CRFs of N neurons were built by randomly sampling their parameter values from a Gaussian distribution with mean equal to the parameters of the observed CRF from the recorded MUA of the corresponding channel, and with standard deviation equal to a 10% of the mean. To model the noise correlations between the responses of neurons from two different channels, we randomly sampled pairwise correlation coefficients from a Gaussian distribution with mean equal to the estimated noise correlation between MUA signals from those two channels for each given day. The standard deviation was selected as a 10% of the mean. To model noise correlations between neurons contributing to the same MUA signal we randomly sampled from a Gaussian distribution with mean 0.15 and standard deviation 0.025, to reflect the fact that close neurons tend to have stronger positive noise correlations. Population responses were generated with the method introduced by Macke et al.[8], which generates Poisson responses for a specified covariance matrix. Because of the computational cost of generating highly dimensional Poisson responses, and since our aim was not to model the original responses but to simulate qualitatively analogous responses to compare information for single and multi unit activity, we used CRFs fron only one of the monkeys (i.e. monkey 2).

We first reproduced the Fisher information analysis for single channels, as shown in Figure 5 (in main text). We compared the results for SUA signals obtained from the single simulated neurons with MUA signals obtained by the aggregation of the responses of several neurons generated in the same channel. Furthermore, we examined the effect of baseline activity matching across sessions by constructing MUA signals from a different number of neurons, and rescaling the responses so that baseline rate remained constant.

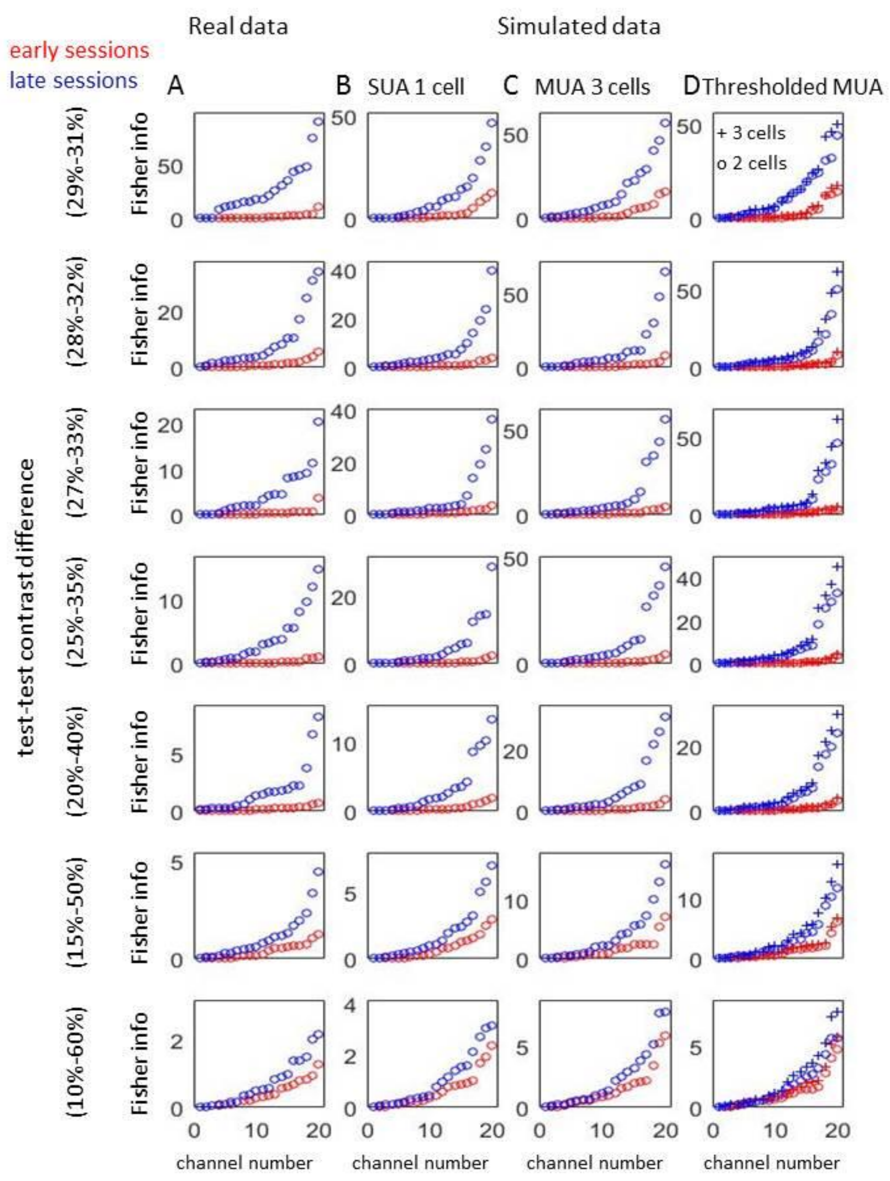

Supplementary Figure 21. ***Rank ordered information content in simulated single channels for different contrast pairs during early (red) and late (blue) periods of learning.*** A) Information from the recorded MUA signals for monkey 2, as shown in Figure 5. B) Information from simulated single-unit activity. C) Information from simulated multi-unit activity generated by the aggregation of activity from 3 cells within each channel. D) Information from multi-unit activity generated by the aggregation of 2 (o) or 3 (+) cells and the application of an adaptive spike threshold to rescale the baseline activity.

As can be seen in Supplementary Figure 21, in all cases the simulated responses reproduce the main results obtained with the real data, namely an increase in information from early to late sessions. This increase is obtained for both the SUA (Supplementary Figure 21B) and MUA signals (Supplementary Figure 21C). The main difference between the information obtained for SUA and MUA signals can be explained by the fact that for Poisson responses the variance is equal to the mean, which means that the aggregation of the responses of several cells tends to reduce the variability of the responses and thus increase information.

The increase in information from early to late sessions also occurs independently of the number of neurons contributing to the thresholded MUA signal (Supplementary Figure 21D). Also here there is a difference in the magnitude on the information depending on the number of cells. Again this can be explained by the reduced variability for higher rates for Poisson responses. In particular, although the mean rates are rescaled by the threshold adaptation (baseline activity matching), the variance of the responses still reflects the original underlying mean. However, the changes in information depending on the number of neurons contributing to the MUA signal is substantially lower than the changes in information from early to late sessions. This suggests that changes in the number of neurons cannot explain the observed increase in information with learning.

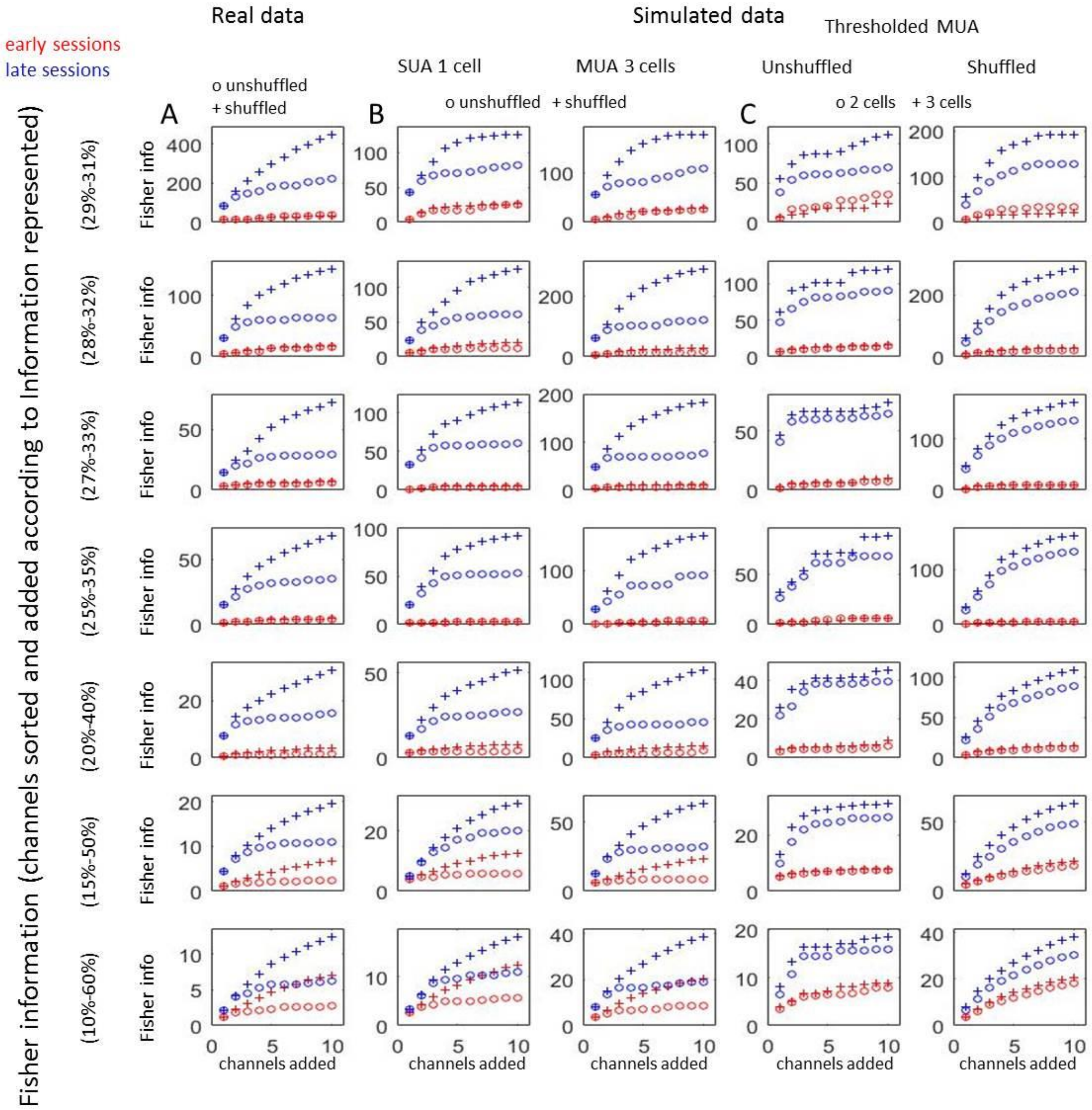

Supplementary Figure 22. ***Information in simulated populations of different sizes before and after learning.*** A) Information obtained from population responses of monkey 2, like in Figure 7A. B) Information obtained from simulated population responses, from single-unit activity and multi-unit activity constructed by the aggregation of the responses of 3 cells. C) Information obtained from the simulated population responses constructed by applying an adaptive threshold to spike detection that rescales the baseline activity independently of the number of neurons contributing to the MUA signal (baseline activity matching).

Similar results were obtained when relaxing the properties of the estimated CRFs preserved in the simulation responses. In particular, the same conclusions about information increases

with learning were also reproduced when the parameters M and R_{max} of the CRFs -which determine the baseline and range of rate values- were selected randomly for each cell and for each day, without preserving the values from the recorded responses (results not shown). Correlation information analysis from the simulated responses also qualitatively reproduced the results found with the recordings, resulting in high correlations between information values for early easy discriminations and for late difficult discriminations, as well as high correlations between information values for early days and the information gain from earlt to late days (results not shown).

We also performed the information analysis for the population responses on the simulated data, analogously to Figure 7 of the main text. Because the generation of simultaneous responses from all channels and several neurons per channel implied the construction of large covariance matrices and the estimation of high-dimensional underlying multivariate Gaussian variables (see Macke et al.[8] for details), we selected only the first 10 most informative channels added in the accumulation of channels in Figure 7. We used the estimated CRFs and noise correlations from these channels to generate simulated population responses as described above.

The simulated responses also reproduced well the observed changes in information with learning at the population level, as shown in Supplementary Figure 22. Consistently with the conclusions from the recorded data, the increase in information for late sessions is higher than what could be obtained from a complete cancelation of noise correlation from the early responses, which indicates that the changes in information are predominantly explained by the increase in information at the single channel level. Again the results are qualitatively equivalent for SUA and MUA signals and differences in the magnitude of information are explained by the reduced variability of Poisson responses for higher rates (Supplementary Figure 22B). Regarding the effect of threshold adaptation (Supplementary Figure 22C), the effect of the number of neurons on the information is substantially lower than the changes observed from early to late days for both the unshuffled and shuffled responses, which again suggests that our conclusions cannot be explained only by changes in the number of cells contributing to the MUA signal.

Overall, the information analysis from simulated Poisson population responses constructed with the estimated CRFs and noise correlations provides further support that the conclusions obtained in the main text from the recorded MUA activity reflect changes with learning that occur at the single cell level. The correspondence of the effects observed with learning on the simulated single unit and multi unit activity is in agreement with the consistency of the results from the recorded MUA activity and the results for single units presented above in the Supplementary Materials.

Supplementary note 11: Accounting for the effect of trial-to-trial activity fluctuations on discriminability and decision related neuronal measures: a COunt Based Estimator (COBE)

A common way to quantify neuronal discriminability has been to calculate the performance of an ideal observer who discriminates between stimuli that vary along an ordinal scale (e.g. the contrast or orientation of gratings, or frequency of flutters in somatosensation). The underlying assumption is that that neuronal response differences are consistent with the stimulus differences. For example, given two stimuli with features s_1 and s_2 such that $s_2 > s_1$, which elicit responses r_1 and r_2, the ideal observer associates s_2 with the higher response, and hence

its decoding performance is quantified by the probability p ($r_2>r_1$). A traditional AUROC analysis estimates this probability based on the assumption that r_1 and r_2 are independently sampled from their distributions on every trial. However, in the case of 2-AFC tasks in which the two stimuli are presented consecutively within a short period of time, such as within one trial, within trial autocorrelations (such as state-dependent gain fluctuations), lead to response co-variations. Neglecting this within-trial autocorrelation of r_1 and r_2 can lead to underestimates regarding the ability to discriminate s_1 and s_2. Here we use a simple nonparametric alternative to the AUROC estimator, which takes these co-variations into account:

$$p(r_2 > r_1) = \int_{-\infty}^{\infty} dr_2 p(r_2) \int_{-\infty}^{r_2} dr_1 p(r_1|r_2) = \frac{n_{r_2>r_1} + 0.5 n_{r_2=r_1}}{N}, (Supplementary\ equation\ \ 6)$$

where $n_{r_2>r_1}$ and $n_{r_2=r_1}$ are the number of trials, out of N, in which r_2 is higher than or equal to r_1, respectively. Since this is based on spike count differences within a single trial, we termed it COunt-Based Estimator (COBE). It is based on a direct measure of the joint probability and provides a hypothesis-free measure of neural discriminability that automatically includes the effects of correlation between the responses. Note that, only if r_1 and r_2 are independent, the integral expression of p(r_2> r_1) is equal to the definition of the AUROC. That is, when p(r_1, r_2)= p(r_1) p(r_2), then AUROC = COBE. Hence, AUROC is a special case of COBE, which would provide an exact description of the test-sample single-trial discriminability ***only*** when there is no within-trial correlation between test and sample response.

As a particular example to understand how the COBE and AUROC measures deal with within-trial correlations between the neural responses we consider the case in which these correlations originated from internal state fluctuations, e.g. leading to co-fluctuations in the response gain. We can then express the performance of the ideal observer in terms of the state value θ:

$$p(r_2 > r_1) = \int d\theta\, p(r_2 > r_1|\,\theta) p(\theta) = \int d\theta [\int_{-\infty}^{\infty} dr_2 p(r_2|\theta) \int_{-\infty}^{r_2} dr_1 p(r_1|r_2,\theta)] p(\theta)$$

$$(Supplementary\ equation\ \ 7)$$

that is, the performance is just the average of the performance for each fixed state value, given the distribution $p(\theta)$ of the states. If it is further assumed that the state fluctuations are responsible for all the correlations between the responses, then for a fixed state the responses are independent, i.e. $p(r_1|r_2,\theta)] = p(r_1|\theta)$, and the AUROC (θ) estimator is a good estimator of the probabilities $p(r_2 > r_1|\theta)$ for a fixed state. However, the performance of the AUROC over multiple states underestimates the discriminability.

To illustrate the differences in the AUROC estimator and COBE estimator, in the following we consider, purely as an illustrative example, neuronal responses jointly modulated by a fluctuating state θ. To obtain simple closed form expressions of the AUROC and COBE values we considered the case in which the responses and the state have a multivariate Gaussian distribution. We stress that both the COBE definition in Supplementary equation 7 and its calculation from the real data does not rely in any way on these assumptions of Gaussianity and of dependence on a fluctuating state, which are used only in the following example and purely for illustrative purposes.

For Gaussian variables the form of the AUROC is well-known[9]:

$$AUROC = p_{ind}\,(r_2 > r_1) = \frac{1}{2} erfc\left(-\frac{1}{2}\frac{\Delta\mu_r}{\sigma_r}\right), \qquad (Supplementary\ Equation\ 8)$$

where refers to the probability for r_1 and r_2 being independent, erfc is the complementary error function, $\Delta\mu_r = \mu_{r_2} - \mu_{r_1}$ is the difference between the mean responses to the test and sample stimuli and σ_r is the responses variance, which we consider to be equal for both responses, for simplicity. Since the responses and the internal state have a multivariate Gaussian distribution, the conditional distribution of the responses for a fixed state is also Gaussian, and thus the same expression holds for AUROC(θ), with $\Delta\mu_r$ and the variance substituted by their conditional counterparts. Given Gaussianity, the conditional mean and conditional variance of the neural responses are:

$$\mu_{r_i|\theta} = \mu_{r_i} + \frac{\sigma_r}{\sigma_\theta}\rho_{r\theta}^2(\theta - \mu_\theta), \qquad \sigma_{r|\theta}^2 = \left(1 - \rho_{r\theta}^2\right)\sigma_r^2,\ Supplementary\ Equation\ 9$$

For simplicity, we also considered that the state has the same influence on within trial sample and test stimulus responses, whereby the influence of the state on the response is determined by the correlation coefficient $\rho_{r\theta}$. Accordingly, $\Delta\mu_{r|\theta} = \mu_{r_2|\theta} - \mu_{r_1|\theta}$ is equal to $\Delta\mu_r$ and it is thus independent of θ. Given Gaussianity, the conditional variance is independent of θ. Therefore, AUROC (θ) does not depend on θ (even if AUROC over many states depends on the state variance). Given this, for this particular example, the general expression of COBE (Supplementary equation 6) leads to:

$$COBE = p(r_2 > r_1) = \frac{1}{2} erfc\left(-\frac{1}{2}\frac{\Delta\mu_r}{\sigma_{r|\theta}}\right) = \frac{1}{2} erfc\left(-\frac{1}{2}\frac{1}{\sqrt{1-\rho_{12}}}\frac{\Delta\mu_r}{\sigma_r}\right),\ Supplementary\ equation\ 10$$

where ρ_{12} is the correlation coefficient between the responses. We assume that within-trial correlations are fully explained by the state fluctuations ($\rho_{12} = \rho_{r\theta}^2$). Comparing the expressions of AUROC and COBE it is clear that the degree of correlation between the responses completely determines their difference. Supplementary Figure 23 illustrates this difference. Supplementary Figure 23A illustrates the relationship between $\Delta\mu_r$ and discriminability performance. The AUROC estimator underestimates the performance in comparison to COBE. This is because it does not take into account the increase in the variability of the responses due to state fluctuations, σ_r in comparison to $\sigma_{r|\theta}$, which in turn increases the overlap of response distributions of r_2 and r_1. Conversely this does not occur when the relative ordering $r_2 > r_1$ within each trial is taken into account (COBE). Supplementary Figure 23b shows the difference $\Delta_{C\text{-}A}$ =COBE – AUROC as a function of the within-trial correlation ρ_{12}. As expected, the difference cancels when there are no correlations, and increases monotonically with the level of correlations. Supplementary Figure 23c shows how both the difference between COBE and AUROC as well as within-trial correlations are determined by the strength of state fluctuations, which, as stated previously, directly affect neuronal responses.

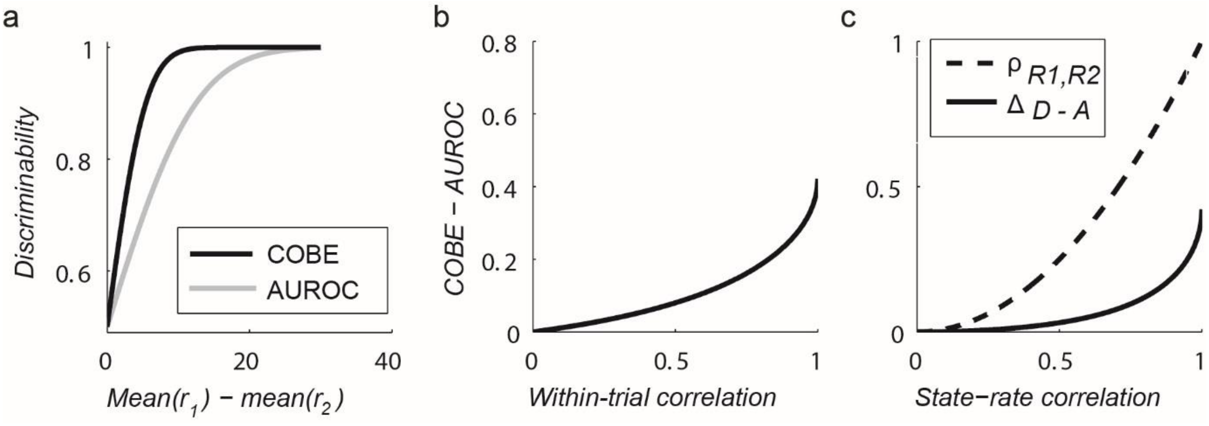

Supplementary Figure 23. Effect of slow correlated activity fluctuations on different measures of ideal observer decoding ability. The difference between the performance's estimator COBE and the traditional estimator AUROC depends on the degree of correlation between the responses, for Gaussian-distributed responses. The parameter value $\sigma_{r|\theta}$ = 3 was fixed in all simulations. (**a**) Plot of COBE and AUROC values as a function of the degree of separation between the two responses distributions (represented by the difference in means, $\Delta\mu_r$). For illustration purpose, $\rho_{r\theta}$=0.9 is fixed. (**b**) Plot of the difference between the estimators ($\Delta_{C\text{-}A}$) as a function of the within-trial correlation coefficient of the firing rates (ρ_{12}). The mean response difference is fixed to $\Delta\mu_r$=6. (**c**) Plot of the difference between the measures ($\Delta_{C\text{-}A}$, solid line), and the correlation coefficient of the firing rates (ρ_{12}, dotted line) as a function of the state-rate correlation ($\rho_{r\theta}$). Again $\Delta\mu_r$=6.

Supplementary note 12: COBE based results

For comparative reasons we now present the figures from the main manuscript that were based on AUROC measures, but using COBE measures instead.

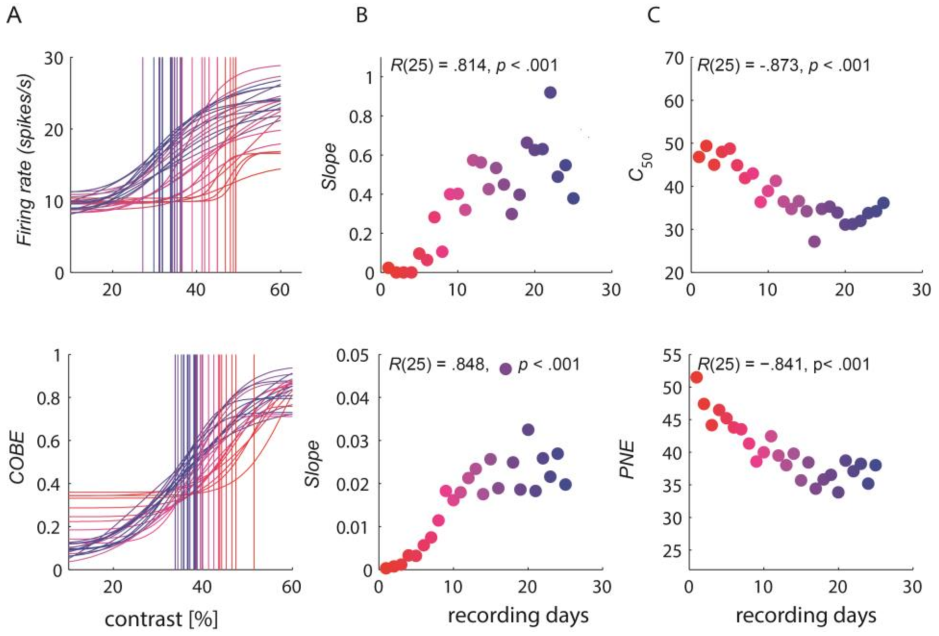

Supplementary Figure 24. *Related to Figure 1. Example channel showing COBE based neurometric data instead of AUROC based data.* A) Single channel contrast response functions and neurometric function as a function of learning (color coded red to blue). Vertical lines show location of C_{50} for each recording day. B) Slope of the contrast response function and neurometric function at 30% (the sample contrast). C) Change of the C_{50} and the PNE with learning.

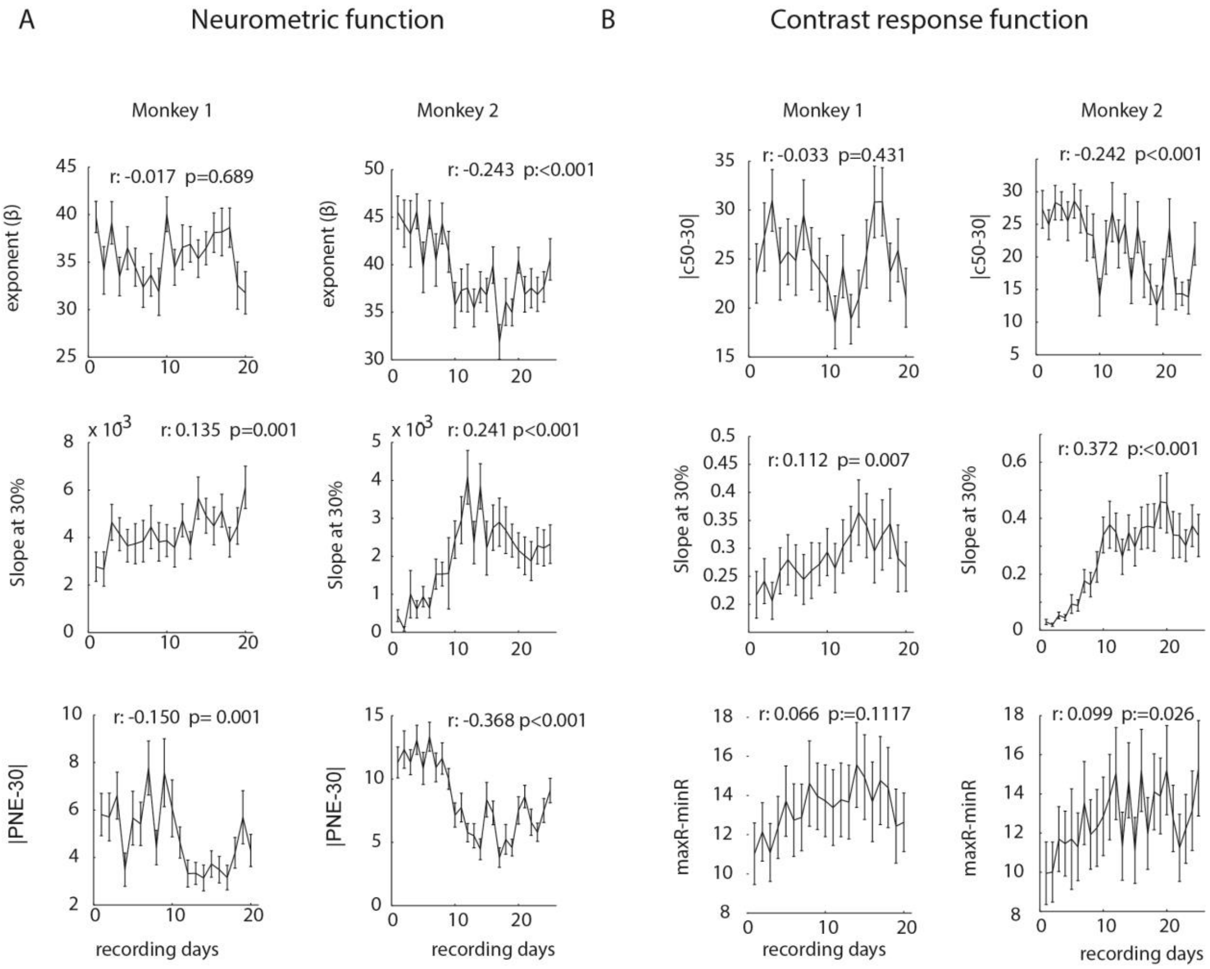

Supplementary Figure 25. ***Related to Figure 2. Learning induced changes in selected parameters of the COBE based neurometric function (fitted with a Weibull function) and of the contrast response function (fitted with a Naka-Rushton function).*** A) Changes in location where the neurometric function reaches 63% of its range, the slope at 30% contrast, and point of neuronal equality of the neurometric function. B) Changes in C_{50} of the Naka Rushton function, its slope at 30%, and rate ranges (difference between minimum and maximum measured activity). Insets show the Spearman rank correlation coefficients (r) and the p-value (p) of the parameter of interest (dependent variable) vs. recording days (independent variable). Error bars denote S.E.M. (n=29 and n=20 per data point for monkey 1 and 2 respectively).

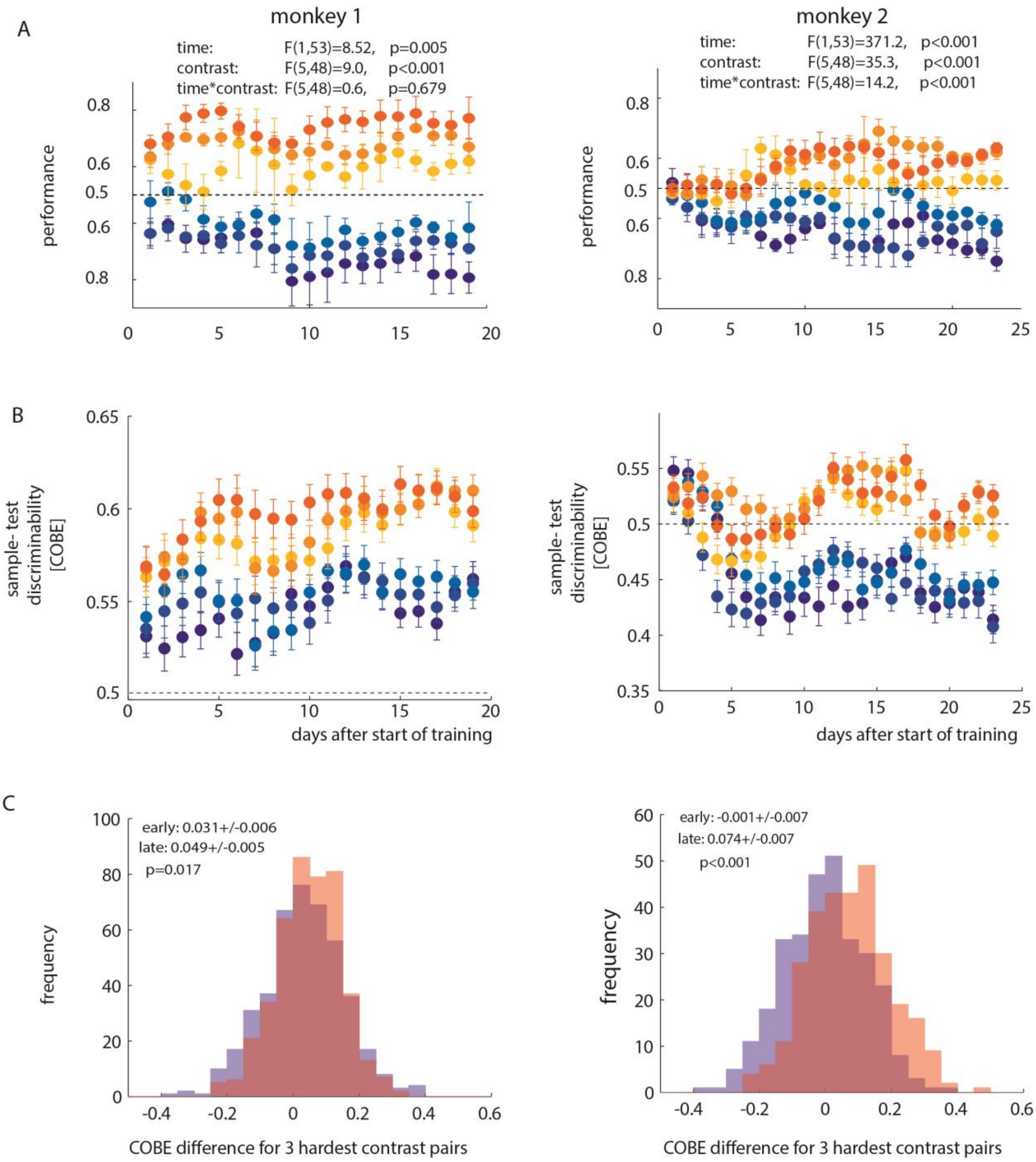

Supplementary Figure 26. ***Related to Figure 3. Changes in behavioural performance and COBE based neuronal discriminability with learning.*** A) Behavioural performance for the 6 most difficult contrast discriminations. Performance for test contrasts lower than sample contrast (blueish colours) is plotted from 0.5 to 1 downwards. Performance for test contrasts higher than sample contrast (reddish colours) from 0.5 to 1 is plotted upwards. Test contrast colour assignment is given by coloured number insets. B) Neuronal discriminability (COBE) for sample-test contrast in the two monkeys with learning. Sample-test contrast colour assignment is given by coloured number insets in panel A. C) Distribution of discriminability difference for the 3 most difficult sample test-contrast comparison pairs (e.g. 31% COBE -29% COBE, 32% COBE -28% COBE, 33% COBE -27% COBE) for the first 5 days of learning (blue) and for the last 5 days of learning (red) across all channels recorded. Darker red shades show overlap of the two distributions. Insets display the mean and S.E.M of the two distributions. P-values indicate whether distributions differed significantly. Performance and discriminability for each data point is the average over 3 consecutive days, i.e. error bars denote SEM of a 3 day performance (COBE) average (thus the number of data points are total number of recording days minus 2).

Supplementary references:

1. Chen, X., Sanayei, M. & Thiele, A. Perceptual learning of contrast discrimination in macaca mulatta. *J Vis* **13**, 22 (2013).

2. Thiele, A. Optimizing brain processing. *Nat Neurosci* **12**, 1359-1360 (2009).

3. Buracas, G.T. & Boynton, G.M. The effect of spatial attention on contrast response functions in human visual cortex. *J Neurosci* **27**, 93-97 (2007).

4. Williford, T., John H. R. Maunsell Effects of Spatial Attention on Contrast Response Functions in Macaque Area V4. *Journal of Neurophysiology* **96**, 40-54 (2006).

5. Zohary, E., Shadlen, M.N. & Newsome, W.T. Correlated neuronal discharge and its implication for psychophysical performance. *Nature* **370**, 140-143 (1994).

6. Panzeri, S., Schultz, S.R., Treves, A. & Rolls, E.T. Correlations and the encoding of information in the nervous system. *Proc R Soc Lond B Biol Sci* **266**, 1001-1012. (1999).

7. Chelazzi, L., Miller, E.K., Duncan, J. & Desimone, R. Responses of neurons in macaque area V4 during memory-guided visual search. *Cereb Cortex* **11**, 761-772. (2001).

8. Macke, J.H., Berens, P., Ecker, A.S., Tolias, A.S. & Bethge, M. Generating spike trains with specified correlation coefficients. *Neural Comput* **21**, 397-423 (2009).

9. Abbott, L.F. & Dayan, P. *Theoretical Neuroscience Computational and Mathematical Modeling of Neural Systems* (MIT Press, 2001).
